# Cross-ancestry GWAS meta-analysis identifies six breast cancer loci in African and European ancestry women

**DOI:** 10.1038/s41467-021-24327-x

**Published:** 2021-07-07

**Authors:** Babatunde Adedokun, Zhaohui Du, Guimin Gao, Thomas U. Ahearn, Kathryn L. Lunetta, Gary Zirpoli, Jonine Figueroa, Esther M. John, Leslie Bernstein, Wei Zheng, Jennifer J. Hu, Regina G. Ziegler, Sarah Nyante, Elisa V. Bandera, Sue A. Ingles, Michael F. Press, Sandra L. Deming-Halverson, Jorge L. Rodriguez-Gil, Song Yao, Temidayo O. Ogundiran, Oladosu Ojengbede, William Blot, Melissa A. Troester, Katherine L. Nathanson, Anselm Hennis, Barbara Nemesure, Stefan Ambs, Peter N. Fiorica, Lara E. Sucheston-Campbell, Jeannette T. Bensen, Lawrence H. Kushi, Gabriela Torres-Mejia, Donglei Hu, Laura Fejerman, Manjeet K. Bolla, Joe Dennis, Alison M. Dunning, Douglas F. Easton, Kyriaki Michailidou, Paul D. P. Pharoah, Qin Wang, Dale P. Sandler, Jack A. Taylor, Katie M. O’Brien, Cari M. Kitahara, Adeyinka G. Falusi, Chinedum Babalola, Joel Yarney, Baffour Awuah, Beatrice Addai-Wiafe, The GBHS Study Team, Stephen J. Chanock, Andrew F. Olshan, Christine B. Ambrosone, David V. Conti, Elad Ziv, Olufunmilayo I. Olopade, Montserrat Garcia-Closas, Julie R. Palmer, Christopher A. Haiman, Dezheng Huo

**Affiliations:** 1 Center for Clinical Cancer Genetics and Global Health, Department of Medicine, University of Chicago, Chicago, IL USA; 2 Department of Preventative Medicine, Keck School of Medicine, University of Southern California, Los Angeles, CA USA; 3 Department of Public Health Sciences, University of Chicago, Chicago, IL USA; 4 Division of Cancer Epidemiology and Genetics, National Cancer Institute, Bethesda, MD USA; 5 Department of Biostatistics, Boston University School of Public Health, Boston, MA USA; 6 Slone Epidemiology Center, Boston University, Boston, MA USA; 7 Usher Institute and CRUK Edinburgh Centre, University of Edinburgh, Edinburgh, UK; 8 Departments of Epidemiology & Population Health and of Medicine (Oncology) and Stanford Cancer Institute, Stanford University School of Medicine, Stanford, CA USA; 9 Biomarkers of Early Detection and Prevention, Department of Population Sciences, Beckman Research Institute, City of Hope Comprehensive Cancer Center, Duarte, CA USA; 10 Division of Epidemiology, Department of Medicine, Vanderbilt Epidemiology Center, Vanderbilt-Ingram Cancer Center, Vanderbilt University, Nashville, TN USA; 11 Department of Public Health Sciences, University of Miami, Miami, FL USA; 12 Department of Radiology, University of North Carolina, Chapel Hill, NC USA; 13 Cancer Prevention and Control Program, Rutgers Cancer Institute of New Jersey, New Brunswick, NJ USA; 14 Department of Pathology, Keck School of Medicine, University of Southern California, Los Angeles, CA USA; 15 Genomics, Development and Disease Section, Genetic Disease Research Branch, National Human Genome Research Institute, NIH, Bethesda, MD USA; 16 Department of Cancer Prevention and Control, Roswell Park Comprehensive Cancer Center, Buffalo, NY USA; 17 Department of Surgery, College of Medicine, University of Ibadan, Ibadan, Nigeria; 18 Center for Population and Reproductive Health, College of Medicine, University of Ibadan, Ibadan, Nigeria; 19 Department of Epidemiology, Gillings School of Global Public Health, University of North Carolina, Chapel Hill, NC USA; 20 Department of Medicine, Perelman School of Medicine, University of Pennsylvania, Philadelphia, PA USA; 21 University of the West Indies, Bridgetown, Barbados; 22 Department of Family, Population and Preventive Medicine, Stony Brook University, Stony Brook, NY USA; 23 Laboratory of Human Carcinogenesis, National Cancer Institute, Bethesda, MD USA; 24 Department of Veterinary Biosciences, College of Veterinary Medicine, The Ohio State University, Columbus, OH USA; 25 Division of Research, Kaiser Permanente Northern California, Oakland, CA USA; 26 Center for Population Health Research, Instituto Nacional de Salud Publica, Cuernavaca, Mexico; 27 Department of Medicine, University of California San Francisco, San Francisco, CA USA; 28 Centre for Cancer Genetic Epidemiology, Department of Public Health and Primary Care, University of Cambridge, Cambridge, UK; 29 Centre for Cancer Genetic Epidemiology, Department of Oncology, University of Cambridge, Cambridge, UK; 30 Biostatistics Unit, The Cyprus Institute of Neurology & Genetics, Nicosia, Cyprus; 31 Epidemiology Branch, National Institute of Environmental Health Sciences, NIH, Research Triangle Park, NC USA; 32 Radiation Epidemiology Branch, Division of Cancer Epidemiology and Genetics, National Cancer Institute, Bethesda, MD USA; 33 Institute for Advanced Medical Research and Training, College of Medicine, University of Ibadan, Ibadan, Oyo Nigeria; 34 Department of Pharmaceutical Chemistry, University of Ibadan, Ibadan, Oyo Nigeria; 35 Korle Bu Teaching Hospital, Accra, Ghana; 36 Komfo Anokye Teaching Hospital, Kumasi, Ghana; 37 Peace and Love Hospital, Kumasi, Ghana

**Keywords:** Breast cancer, Cancer epidemiology

## Abstract

Our study describes breast cancer risk loci using a cross-ancestry GWAS approach. We first identify variants that are associated with breast cancer at P < 0.05 from African ancestry GWAS meta-analysis (9241 cases and 10193 controls), then meta-analyze with European ancestry GWAS data (122977 cases and 105974 controls) from the Breast Cancer Association Consortium. The approach identifies four loci for overall breast cancer risk [1p13.3, 5q31.1, 15q24 (two independent signals), and 15q26.3] and two loci for estrogen receptor-negative disease (1q41 and 7q11.23) at genome-wide significance. Four of the index single nucleotide polymorphisms (SNPs) lie within introns of genes (*KCNK2*, *C5orf56*, *SCAMP2*, and *SIN3A*) and the other index SNPs are located close to *GSTM4*, *AMPD2*, *CASTOR2*, and *RP11-168G16.2*. Here we present risk loci with consistent direction of associations in African and European descendants. The study suggests that replication across multiple ancestry populations can help improve the understanding of breast cancer genetics and identify causal variants.

## Abstract (web summary)

GWAS have enhanced our understanding for the genetic basis of breast cancer, but the majority of them were performed for European ancestry populations. Here, the authors use a cross-ancestry approach and report seven new variants associated with breast cancer risk among women of African ancestry.

## Introduction

Breast cancer is the most common cancer in women worldwide and accounted for 2.1 million new cases and 627,000 deaths in 2018^1^. Studies have shown a significant contribution of genetic factors to breast cancer risk^2,3^, yet the landscape of this contribution has not been fully elucidated. Mutations in high- and moderate-penetrance genes confer relatively high risks of breast cancer but are rare in the population and account for <5–10% of cases^4^. Genome-wide association studies (GWAS) have been successful in identifying common low-penetrance genetic variation and approximately 200 risk loci have now been identified^5–7^. The risk loci so far identified have provided clues to elucidating breast cancer tumorigenesis through previously unknown mechanisms. Additionally, when combined into risk scores, these polymorphisms can be used for breast cancer risk prediction^8^.

Despite the usefulness of GWAS, the majority of the GWAS studies have been performed among European ancestry populations^9–13^, it is unclear whether the same genetic risk factors are also important in other populations, which may limit the applicability of the findings to other groups^14^. The earliest GWAS conducted in African ancestry populations identified genetic variants at 5p15.33 (TERT-CLPTM1L) associated with estrogen receptor (ER) negative breast cancer^15^. A larger analysis of African ancestry individuals which included several consortia identified a SNP at 3q26.21 also associated with ER-negative breast cancer^16^. Some common susceptibility loci are shared across populations, and the shared disease-associated variants are more likely to be causal^6,9,14^.

Here we present, using a cross-ancestry GWAS approach in 248,000 women, genetic risk variants at 1p13.3, 5q31.1, 15q24, and 15q26.3 for overall breast cancer, and at 1q41 and 7q11.23 for ER-negative disease. The consistency of the directions of the risk for these loci in African and European samples increases the likelihood of their being causal variants.

## Results

We discovered six loci containing seven SNPs significantly associated with breast cancer at *P* < 5 × 10^−8^ on cross-ancestry meta-analysis, with odds ratios (OR) ranging from 0.95 to 1.05 (Tables 1, 2; Supplementary Figs. 1, 2). Five SNPs were associated with overall breast cancer risk (rs17024628 at 1p13.3, rs2522057 at 5q31.1, rs1869959 at 15q24.1, rs60381548 at 15q24.2, rs181337095 at 15q26.3) and two were associated with ER-negative breast cancer (rs67931591 at 1q14 and rs1637365 at 7q11.2). The two SNPs at the 15q24 region were about 582 kb apart and independently associated with breast cancer risk. Four SNPs were within genes (rs67931591 in *KCNK2*, rs2522057 in *C5orf56*, rs1869959 in *SCAMP2*, and rs60381548 in *SIN3A*) and the others were in intergenic regions. The direction of the associations was consistent for the pooled African and European estimates. The estimates for overall and ER-negative breast cancer were generally consistent across the five contributing studies of African ancestry participants (Supplementary Table 2) and the BCAC European datasets (Supplementary Table 3).

**Table 1:** Novel breast cancer risk loci identified by cross-ancestry meta-analysis of African and European populations.

							African-specific meta-analysis			European-specific meta-analysis			Combined African and European meta-analysis^a^		
SNP	Chr	Position	Test	Other	Locus	Within gene	TAF	OR (95% CI)	*P* value	TAF	OR (95% CI)	*P* value	TAF	OR (95% CI)	*P* value
*Overall*															
rs17024629	1	110,179,756	T	C	1p13.3	No	0.13	0.88 (0.83–0.95)	5.2E−04	0.16	0.96 (0.94–0.98)	1.2E−06	0.16	0.95 (0.94–0.97)	3.0E−08
rs67931591	1	215,330,292	G	GCTGAGG- CAGGAGA	1q41	KCNK2	0.28	0.95 (0.90–1.00)	0.034	0.68	0.98 (0.96–0.99)	3.9E−04	0.66	0.97 (0.96–0.99)	7.4E−05
rs2522057	5	131,801,947	C	G	5q31.1	C5orf56	0.86	0.92 (0.86–0.98)	0.0084	0.59	0.97 (0.96–0.98)	9.3E−08	0.60	0.97 (0.95–0.98)	1.1E−08
rs1637365	7	74,359,358	T	C	7q11.23	No	0.62	1.06 (1.01–1.12)	0.024	0.28	1.04 (1.02–1.05)	3.3E−06	0.31	1.04 (1.02–1.05)	3.6E−07
rs1869959	15	75,147,332	A	C	15q24.1	SCAMP2	0.40	0.95 (0.91–1.00)	0.043	0.30	0.97 (0.95–0.98)	3.6E−07	0.30	0.96 (0.95–0.98)	4.6E−08
rs60381548	15	75,728,474	CA	C	15q24.2	SIN3A	0.50	0.93 (0.89–0.97)	0.0016	0.25	0.96 (0.95–0.98)	4.0E−07	0.27	0.96 (0.95–0.97)	6.6E−09
rs181337095	15	100,907,094	A	G	15q26.3	No	0.69	1.06 (1.01–1.12)	0.017	0.87	1.05 (1.03–1.07)	3.4E−07	0.84	1.05 (1.04–1.07)	1.8E−08
*ER negative*															
rs17024629	1	110,179,756	T	C	1p13.3	No	0.13	0.83 (0.74–0.92)	0.00064	0.16	0.96 (0.93–0.99)	0.020	0.16	0.95 (0.93–0.98)	0.0014
rs67931591	1	215,330,292	G	GCTGAGG- CAGGAGA	1q41	KCNK2	0.29	0.92 (0.85–0.99)	0.024	0.68	0.94 (0.92–0.96)	4.6E−07	0.65	0.94 (0.92–0.96)	4.3E−08
rs2522057	5	131,801,947	C	G	5q31.1	C5orf56	0.86	0.93 (0.85–1.03)	0.18	0.59	0.99 (0.96–1.01)	0.22	0.60	0.98 (0.96–1.01)	0.14
rs1637365	7	74,359,358	T	C	7q11.23	No	0.61	1.15 (1.06–1.25)	0.00069	0.28	1.07 (1.04–1.10)	9.0E−07	0.32	1.08 (1.05–1.11)	1.0E−08
rs1869959	15	75,147,332	A	C	15q24.1	SCAMP2	0.41	0.96 (0.89–1.03)	0.22	0.30	0.97 (0.94–0.99)	0.0050	0.31	0.96 (0.94–0.99)	0.0022
rs60381548	15	75,728,474	CA	C	15q24.2	SIN3A	0.51	0.99 (0.92–1.06)	0.78	0.25	0.95 (0.92–0.97)	8.6E−05	0.28	0.95 (0.93–0.98)	1.7E−04
rs181337095	15	100,907,094	A	G	15q26.3	No	0.69	1.07 (0.99–1.16)	0.067	0.87	1.06 (1.02–1.10)	0.0016	0.83	1.06 (1.03–1.10)	2.9E−04

*TAF* Test allele frequency, *SNP* single nucleotide polymorphism, *OR* odds ratio, *CI* confidence intervals.

^a^Test for heterogeneity across studies was statistically significant only for rs1637365 and ER-negative breast cancer (*P*-for-heterogeneity = 0.025).

**Table 2:** Association analysis of novel SNPs in cross-ancestry combined meta-analysis by estrogen receptor status.

							ER positive			ER negative			*P* for heterogeneity^a^
SNP	Chr	Position	Test	Other	Locus	Nearest genes	TAF	OR (95% CI)	*P* value	TAF	OR (95% CI)	*P* value	
rs17024629	1	110,179,756	T	C	1p13.3	GSTM4, AMPD2, GSTM2, GSTM1, GNAT2, MIR197, GNAI3	0.16	0.95 (0.93–0.97)	1.1E−06	0.16	0.95 (0.93–0.98)	0.0014	0.96
rs67931591	1	215,330,292	G	GCTGAGG- CAGGAGA	1q41	KCNK2, KCTD3, CENPF	0.66	0.98 (0.96–0.99)	0.0020	0.65	0.94 (0.92–0.96)	4.3E−08	0.003
rs2522057	5	131,801,947	C	G	5q31.1	C5orf56, IRF1, SLC22A5, IL5, RAD50	0.60	0.96 (0.95–0.98)	2.9E−07	0.60	0.98 (0.96–1.01)	0.14	0.011
rs1637365	7	74,359,358	T	C	7q11.23	GTF2IRD2, STAG3L2, PMS2P5, WBSCR16	0.30	1.02 (1.00–1.04)	0.026	0.32	1.08 (1.05–1.11)	1.0E−08	3.9E−04
rs1869959	15	75,147,332	A	C	15q24.1	CSK, CYP1A2, ULK3, MPI, SCAMP2, CPLX3, ARID3B, PTPN9	0.30	0.96 (0.94–0.97)	6.0E−08	0.31	0.96 (0.94–0.99)	0.0022	0.64
rs60381548	15	75,728,474	CA	C	15q24.2	PTPN9, SCAMP5, C15orf39, COMMD4, SIN3A, SNUPN	0.27	0.96 (0.95–0.98)	1.1E−05	0.28	0.95 (0.93–0.98)	1.7E−04	0.47
rs181337095	15	100,907,094	A	G	15q26.3	ADAMTS17, PCSK6	0.85	1.05 (1.03–1.08)	1.1E−05	0.83	1.06 (1.03–1.10)	2.9E−04	0.60

*TAF* Test allele frequency, *SNP* single nucleotide polymorphism, *OR* odds ratio, *CI* confidence intervals.

^a^*P* for heterogeneity between ER-positive and ER-negative tumors.

Conditional analysis revealed three additional independent signals significant at *p* < 10^−4^ at the 1p13.3 locus (rs116363925, rs114351980, and 1:109969874:C:T), two independent signals at 15q24 (rs113939578, rs12917507), and one each at 5q31.1 (5:132149322:G:GGCCGCCGCC) and 15q26.3 (rs117793215) for overall breast cancer risk. Another independent SNP at 1q41 that was associated with ER-negative breast cancer was rs5780828 (Table 3).

**Table 3:** Conditional regression analysis of top SNPs and others in the loci.

				Marginal analysis		Conditional on lead SNP		Conditional on all other independent SNPs at locus^a^	
Locus	Variants	Position	Test/other alleles	OR (95% CI)	*P* value	OR (95% CI)	*P* value	OR (95% CI)	*P* value
Overall									
1p13.3	**rs17024629**	110,179,756	T/C	0.95 (0.94–0.97)	3.0E−08			0.96 (0.94–0.97)	1.07E−07
1p13.3	rs116363925	109,926,599	T/G	0.94 (0.91–0.97)	1.3E−04	0.94 (0.91–0.97)	1.4E−04	0.93 (0.90–0.96)	1.46E−06
1p13.3	rs114351980	110,219,028	C/T	1.08 (1.04–1.12)	7.3E−05	1.08 (1.03–1.11)	8.2E−05	1.08 (1.04–1.12)	1.80E−05
1p13.3	1:109969874:C:T	109,969,874	T/C	1.04 (1.02–1.06)	2.7E−04	1.04 (1.02–1.06)	1.8E−04	1.05 (1.02–1.07)	3.41E−05
15q24.2	**rs60381548**	75,728,474	CA/C	0.96 (0.95–0.97)	6.6E−09			0.97 (0.96–0.99)	8.62E−09
15q24.1	**rs1869959**	75,147,332	A/C	0.96 (0.95–0.98)	4.6E−08			0.96 (0.95–0.97)	1.57E−09
15q24.2	rs113939578^b^	75,479,704	T/C	0.96 (0.94–0.98)	8.0E−05	0.96 (0.94–0.98)	1.9E−05	0.96 (0.94–0.98)	9.13E−06
15q24.2	rs12917507^b^	75,953,903	T/G	1.02 (1.01–1.03)	0.0030	1.02 (1.01–1.03)	1.3E−04	1.02 (1.01–1.04)	6.51E−05
15q26.3	**rs181337095**	100,907,094	A/G	1.05 (1.04–1.07)	1.8E−08			1.05 (1.04–1.08)	1.26E−08
15q26.3	rs117793215	100,535,681	T/C	0.93 (0.89–0.97)	1.6E−04	0.93 (0.89–0.96)	7.7E−05	0.93 (0.89–0.96)	7.71E−05
5q31.1	**rs2522057**	131,801,947	C/G	0.97 (0.95–0.98)	1.1E−08			0.96 (0.95–0.97)	2.39E−10
5q31.1	5:132149322:G:GGCCGCCGCC	132,149,322	GGCCGCCGCC/G	1.03 (1.01–1.04)	7.4E−04	1.03 (1.02–1.05)	2.1E−05	1.04 (1.02–1.05)	2.13E−05
ER negative									
1q41	**rs67931591**	215,330,292	G/GCTGAGGCAGGAGA	0.94 (0.92–0.96)	4.3E−08			0.94 (0.93–0.96)	1.59E−12
1q41	rs5780828	215,416,434	TA/T	0.96 (0.93–0.98)	9.7E−05	0.94 (0.92–0.96)	1.5E−06	0.94 (0.92–0.96)	1.46E−06

*SNP* Single nucleotide polymorphism, *OR* odds ratio, *CI* confidence intervals.

^a^All independent SNPs (Joint ORs) at each locus were in the same model.

^b^The conditional ORs are conditioned on rs60381548 and rs1869959.

Concerning pleiotropy, none of the SNPs identified above have been reported in previous GWAS associations at genome-wide significance with cancers. Associations with mosquito bite size and asthma had been reported for rs2522057 and SNPs in LD with this lead SNP. For the 15q24 region, associations with cardiovascular phenotypes have been previously reported for rs1869959 while body height, glomerular filtration rate, and type 2 diabetes have been associated with rs60381548 and SNPs highly correlated with this lead SNP (Supplementary Table 4).

The eQTL analysis of breast tumors revealed significant associations in four loci: 1p13.3, 5q31, 15q24.1, and 15q24.2 (Supplementary Table 5A). There were significant associations (*P* < 10^−6^) between the protective allele of rs17024629 (T allele) at 1q13.3 and increased expression of *GSTM1, GSTM2*, and *GSTM4*, which are located 19 kb, 31 kb, and 51 kb downstream of the SNP. At 5q31, the top SNP rs2522057, located 15 kb downstream of *IRF1*, was most significantly associated with the gene’s expression levels. At 15q24.1, rs1869959, located 35 kb upstream of *MPI* and 12 kb upstream of *ULK3*, was significantly associated with the expression of these two genes. The other top SNP at the 15q24 locus, rs60381548, located intron of *SIN3A*, 30 kb downstream of *PTPN9*, 162 kb downstream of *SNUPN*, and 212 kb upstream of *SNX33* was correlated with all four genes. The 1q41 locus revealed a significant association between rs67931591 and *PTPN14*. The SNP at 7q11.23 was significantly correlated with *STAG3L2*, a pseudogene. Previous published report on normal breast tissues from the GTEx revealed associations between rs2522057 and the *SLC22A5* gene, and between rs1869959 and the *ULK3* gene (Supplementary Table 5B).

Functional annotation analyses pointed out relationships with genomic functional biofeatures for rs2522057, rs17024629, rs1869959, and rs60381548 or SNPs in strong LD with these top SNPs in breast tissue-originated cell lines (Supplementary Tables 6, 7A, 7B). Active enhancer and promoter states were found for SNPs in strong LD with rs2522057 (rs2188962, rs4705950, rs4705950, rs72797306, rs11741255) using the 25-state chromatin model. Additional associations were found with histone modifications. These included: H3K4me1 and H3K27ac enhancer peaks for: rs2522057 and other SNPs in strong LD (rs2188962, rs17622378, rs12521868, rs146604341, rs11951091, rs6866614, rs4705950, rs72797303, rs2706396, rs2522052, rs2706403, rs2706336, rs72797306, rs2248116, rs11741255); those in strong LD with rs17024629 (rs538388, rs560674, rs568686, rs669426, rs3850616, rs17024628); a SNP in strong LD with rs1869959 (rs7180432); and for the top SNP rs60381548. H3K4me3 and H3K9ac promoter peaks were found for: rs2522057 and other SNPs in strong LD (rs12515180, rs11951091, rs72797306); SNPs in LD with rs17024629 (rs538388, rs669426, rs3850616); rs1869959 and other SNPs in strong LD (rs4886613, rs936230).

We evaluated the consistency of the association of the identified loci in Latinos, and found the effect and direction of the association were consistent in 8 out of 11 evaluated variants (Supplementary Table 8). However, none of these consistent variants was statistically significant at *p* < 0.05 in the Latino study of 2385 cases and 6416 controls.

## Discussion

We found seven variants associated with breast cancer risk among women of African ancestry that may contribute to better prediction of breast cancer risk and provide further insights into mechanisms of breast cancer carcinogenesis. Although the discovery of the loci is largely driven by effects in European ancestry populations, observation of risk loci in multiple ancestral populations lends credence to the chances of those variants being causal. We designed our current approach of cross-ancestry meta-analysis to uncover genetic variants shared across ancestry.

The SNPs identified in this study lie in regions that are close to genes that have been previously implicated in cancer. Interestingly we found three variants located within the introns of genes. One of the variants, rs67931591 was found in *KCNK2* (also known as *TREK1)*, which encodes the protein potassium channel subfamily K member 2, a member of the two-pore-domain background potassium channel family. Potassium channels are known to play a role in cancer and studies using TCGA data have shown associations with DNA methylation in the *KCNK* genes and triple negative breast cancer. Additionally, overexpression of *KCNK5, KCNK9*, and *KCNK12* and under-expression of *KCNK6* and *KCNK15* were associated with triple negative breast cancer^17^. Other studies investigated expression of *KCNK2* gene as potential prognostic markers. For example, Innamaa et al.^18^ found increased *KCNK2* expression in human ovaries and a role in cell proliferation and apoptosis for *KCNK2* modulators in ovarian cancer cell lines. Li et al.^19^ found differential expression of *KCNK2, KCNK15 and KCNK17* in liver cancer cells compared to healthy tissue. KCNK2 has also been reported in amplified regions in a genome-wide scan of chromosomal alterations in esophageal squamous cell carcinoma^20^.

We found two independent SNPs at the 15q24 locus at about 582 kb apart (rs1869959 at 15q24.1 in the *SCAMP2* intron and rs60381548 at 15q24.2 in the *SIN3A* gene). The *SIN3A* gene was associated with rs60381548 in the eQTL analysis of breast tumor in the present study. Switch-independent 3 family A (*SIN3A)* is a transcriptional regulator, that along with its paralog and corepressor play important roles in normal breast development, cancer and metastasis^21–23^. Furthermore, *SIN3A* mediates STAT3 transcriptional repressor activity^24^ and along with genes involved in histone modification such as *HDAC* and Lysine specific demethylase (*LSD)*, inhibits several cancer genes including *CASP7, TGFB2, CDKN1A, HIF1A, TERT and MDM2*^25^. Studies have shown key roles for SIN3A in breast cancer including sensitivity to chemotherapy^25^ and breast cancer progression^26,27^.

The other SNP at 15q24, rs1869959, is located in the intron of the *SCAMP2* gene that codes for secretory carrier associated membrane protein 2 that functions as carriers to the cell surface in post-golgi recycling pathways^28^. The recent GTEx project pilot study found significant associations between the SNP and SCAMP2 in esophageal mucosa, *ULK3* in breast mammary tissue, adipose, whole blood, and lung tissue^29,30^. We also found that rs1869959 was associated the expression of *ULK3* in breast tumor. *ULK3* is a serine threonine kinase that activates GLI2, a key component of the Hedgehog signaling pathway, and implicated in many cancers^31,32^.

Similarly, the *C5orf56* gene harboring the rs2522057 SNP returned no interesting associations with cancer. However, nearby genes in the 5q31 locus included *RAD50*, that codes for a DNA repair protein, a part of the MRE11-RAD50-NBS1 complex^33^. Other nearby genes include *SLC22A5* solute carrier family 22 member 5 encoding the OCTN2 (organic cation transporter protein), and *IRF1* that encodes interferon regulatory factor 1. *SLC22A5* is an estrogen-dependent gene whose expression is associated with ER status in breast cancer cell lines and tissue specimens^34^. Significantly decreased levels of SLC22A5 have been reported in colorectal cancer tissues compared to normal tissues in eQTL studies^35^. Moreover, eQTL studies report associations between rs2522057 and gene expression in several tissues including breast mammary tissue, lymphocytes, esophageal mucosa, lung, skeletal muscle, skin, thyroid and whole blood^29,30^. We found an association between rs2522057 and *IRF1* expression levels in the eQTL analysis of breast cancer in this study lending support to the likelihood of involvement of the *IRF1* gene in the mechanism of the SNP on breast cancer carcinogenesis. Additionally, IRF1 has been shown to have tumor suppressor functions in breast cancer through its inhibition of NF-kB^36^ and *CASP8* activation and induction of apoptosis^37^.

The majority of GWAS-identified SNPs were located in non-coding regions of the genome, and three loci in the present study were found in intergenic regions. The closest gene to rs17024629 is *AMPD2* (high adenosine monophosphate deaminase 2) and has recently been shown to predict worse outcomes in undifferentiated pleomorphic sarcoma^38^. Earlier studies^39^ found high expression levels of AMPD2 in hepatocellular carcinoma, though the levels did not differ substantially from those in the non-tumorous organ. It is noteworthy that our eQTL analysis did not find a significant association with AMPD2 expression. The carcinogen metabolism genes, *GSTM1, GSTM2*, and *GSTM4* are also located in this region and our eQTL analysis of breast tumor revealed highly significant associations between rs17024629 and these genes. The *GSTM1* null genotype has been associated with several cancers including cancers of the colorectum, oral cavity, lung, cervix, and stomach^40–47^. In eQTL studies, GSTM4 was significantly associated with gene expression in several tissues including the aorta, lungs, tibia nerve and whole blood^29,30^.

The rs1637365 SNP at the 7q11.23 locus is near the *CASTOR2* gene (cytosolic arginine sensor for MTORC1 protein, also known as *GATSL1*, GATS-like protein 1). The CASTOR proteins are arginine sensors that function as negative regulators of the TORC1 signaling pathway, an often dysregulated pathway in human cancer, through the GATOR complex, inhibiting mTORC1^48,49^. The rs181337095 SNP is located 6 kb 5′ of RP11-168G16.2, an antisense DNA.

A potential limitation of this study is the different genotyping platforms used by the different consortia. However, stringent QC measures pre- and post-imputation were carried out. Additionally, the meta-analysis did not reveal significant heterogeneity across studies. Secondly, the sample size for ER-negative breast cancer cases was relatively small, thus reducing the precision of the estimates and providing less power for detecting risk loci. The third limitation is related to the additional SNPs identified at the same loci with the index SNPs from the conditional regression analysis. The regression procedures were based on a liberal *p* value cutoff of 10^−4^, and the chance that some of the identified SNPs could be spurious findings cannot be ruled out. Another noteworthy point is that identification of genetic variants in GWAS is just the first step of the discovery of true causal variants and genes associated with breast cancer. Further studies are needed, including in vitro and in vivo functional studies to elucidate the mechanisms by which identified putative causal variants are acting and identify the targeted genes, Finally, although the direction and strength of the associations were consistent between African and European populations, and mostly consistent with Latino populations, we could not find statistically significant replication of the identified variants, which are likely due to the modest sample sizes of the Latino study.

Our study found six loci that could provide further insights into pathways for breast cancer carcinogenesis. The genetic variants that shared across ancestry populations makes them possible causal variants. Functional studies on these loci are desirable to identify causal variants and elucidate the mechanisms of breast cancer carcinogenesis. In addition, future studies can evaluate these variants for breast cancer risk prediction, particularly in African ancestry populations.

## Methods

### Study population

Data for this study were obtained from four consortia of African ancestry populations (ROOT, AMBER, AABC, and BCAC-African ancestry)^16^ and the Ghana Breast Health Study (GBHS)^50,51^, with a combined sample size of 19434 participants including 9241 cases and 10193 controls (Supplementary Table 1). Estimates from these studies were meta-analysed to generate pooled African ancestry estimates of breast cancer risk. Additionally, we used summary estimates (odds ratios, ORs) of breast cancer from European ancestry BCAC datasets (GWAS, iCOGs and OncoArray) with a combined sample size of 228,951 (122,977 cases and 105,974 controls)^6^.

### Genotyping and quality control

Genotyping and quality control (QC) procedures have been described in detail for the three consortia^16^ and the BCAC European ancestry data^6^. The AABC was genotyped using the Illumina Human 1M-Duo BeadChip. After QC, a total of 3007 cases (1518 ER-positive, 987 ER-negative) and 2720 controls remained in the analysis^52^. Genotyping in the ROOT consortium was done using Illumina HumanOmni 2.5-8v1 array and 1657 cases (374 ER-positive, 403 ER-negative) and 2029 controls passed QC. In the BCAC-African ancestry consortium, genotyping was done using the Illumina OncoArray (260K GWAS backbone) and after removing overlapped samples between OncoArray with AABC, AMBER and ROOT and samples failed in QC, a total of 2271 cases (1130 ER-positive, 613 ER-negative) and 1406 controls remained for analysis. The Illumina MEGA array was used for genotyping in the AMBER consortium, and 1407 cases (952 ER-positive, 385 ER-negative) and 2408 controls remained in analysis passed QC. In the GBHS, Illumina Global Screening Array was used for genotyping, and 899 cases (296 ER-positive, 277 ER-negative) and 1630 controls were included in analysis. Imputation for all studies was done using the cosmopolitan reference panel in the 1000 Genomes Project (Phase 3 release).

In addition, we examined the association between the identified SNPs of interest and breast cancer risk in a GWAS of Latinos (2385 cases and 6416 controls). Details of the genotyping, QC and data analysis have been published^53^.

### Data analysis

#### GWAS

In the ROOT and AABC GWAS studies, genotyped SNPs were analyzed and imputed with imputation score >0.3 and minor allele frequency >0.01 to account for uncertainty in imputation. Unconditional logistic regression was used to examine the association of each SNP and breast cancer risk adjusting for age, study site and eigenvectors from Principal Components Analysis (PCA). In the ROOT GWAS, the first four eigenvectors were used to control for population stratification as only the first 4 eigenvectors were associated with case status. The AABC GWAS adjusted for the first 10 eigenvectors from the PCA. OR and 95% confidence intervals (CI) were calculated from the multivariable logistic regressions. All tests of statistical significance were two sided. Using similar methods, separate analyses were conducted to compare ER-positive and ER-negative breast cancers with controls. The AMBER consortium estimated ORs and *P* values using unconditional logistic regression, adjusting for 10-year age group, sample type (saliva, blood, other), study (Black Women’s Health Study (BWHS) versus others) and PCs that associated with breast cancer at *P* < 0.1. The GBHS estimated per-allele ORs and 95% CI for each SNP on allele counts (dosages) using unconditional logistic regression adjusting for the first ten principal components, self-reported ethnicity and age. In the Oncoarray African ancestry samples, a total of 27 million SNPs with MAF ≥ 0.1% and imputation quality score ≥0.3 were included in the analysis. PCs were estimated using EIGENSTRAT. ORs and *P* value of each SNP were estimated using unconditional logistic regression, adjusting for age, study (Women of African Ancestry Breast Cancer Study—WAABCS versus other) and the first ten PCs.

The BCAC European study used a two-stage imputation approach, using SHAPEIT2 for phasing and IMPUTE version 2 for imputation. The first ten principal components and country were adjusted for in the logistic regression, and per-allele ORs and standard errors were computed^6^.

#### Meta-analysis

Regression coefficient estimates from the five contributing African ancestry studies were combined in a fixed effects meta-analysis using METAL^54^. Variants associated with breast cancer at *P* < 0.05 from the African ancestry meta-analysis were then combined in another fixed effects meta-analysis with the coefficients from the BCAC European ancestry data. Heterogeneity in both meta-analyses was assessed using the *I*^2^ statistic. SNPs that were significant genome-wide (*P* < 5 × 10^−8^) in the cross-ancestry meta-analysis, and >500 kb away from the 180 loci known to be associated with breast cancer risk were identified^5,6^. Conditional analysis below confirmed the identified loci. All analyses were done separately for ER-positive, ER-negative, and overall breast cancer risk.

#### Regression analysis conditional on index SNPs

In order to identify independent SNPs in the identified loci, conditional analysis was done in each of the regions, including all variants in the flanking ±500 kb region of the lead SNP. The 15q24 region had two SNPs about 582 kb apart that were both genome-wide significant (see results for details). Hence, all variants in the region extending from 500 kb upstream of the proximal SNP and 500 kb downstream of the other SNP were included in the conditional analysis for this region. We used the GCTA software with the –COJO option^55^, that utilizes summary statistics and population-specific linkage disequilibrium (LD) from 1000 Genomes Project, for the computation of conditional beta coefficients. SNPs significant at *p* < 10^−4^ after adjusting for lead SNP were considered as independent signals. The *p* < 10^−4^ cutoff was derived by applying a factor of 3000 (the ratio of the 3 billion base pairs genome-wide to the 1 million base pairs in each region in the conditional analysis) to the GWAS significance of 5 × 10^−8^. This procedure was repeated until no additional independent signals were significant. In addition to the conditional analysis involving the lead SNP and one other candidate SNP, we also determined joint ORs including all independent loci in the same model. Separate analyses were done for African and European ancestry data, and the estimates from the conditional analysis were combined in a meta-analysis.

### Functional annotation

The functional annotations of the SNPs were determined using HaploReg v4.1^56^. Using data from ENCODE^57^ and the Roadmap Epigenomics Consortium^58^, we examined the chromatin states including core 15-state model and 25-state model using 12 imputed marks, H3K4me1 and H3K27ac (enhancers), and H3K4me3 and H3K9ac (promoters) for each identified SNP and other SNPs in strong LD with these lead SNPs (>0.8). We also assessed evolutionary conserved regions, DNase hypersensitivity sites, and variant effect on regulatory motifs, proteins bound and eQTL hits from previous studies.

### eQTL analysis

We carried out a cis-eQTL analysis to understand possible target genes in the six loci. All genes within ±1 MB around each index SNP were evaluated and gene expression in breast tumors from TCGA breast cancer patients (African ancestry, *n* = 164 and European ancestry, *n* = 778) were used in the analysis. A linear regression model estimated additive effects for each SNP, adjusting for age, ancestry, copy number variation, batch effect, and molecular subtype. Separate analyses were done for African and European ancestry samples and the estimates were meta-analysed to obtain overall estimates. Bonferroni significance levels were applied to determine statistical significance. We also checked associations between the identified loci and gene expression in several tissues, including normal breast, that had been published from previous eQTL analyses on the Haploreg website.

### Allelic pleiotropy

We assessed the GWAS catalog (www.ebi.ac.uk) for previously reported associations for the identified lead SNPs and all other SNPs in LD with *r*^2^ > 0.4 and phenotypes.

### Ethical approval

Informed consent was obtained from all subjects included in the analysis. The relevant ethical review boards at all participating institutions approved study protocols.

## Supplementary information

Supplemental information

## Data Availability

The genotype datasets used in this study are publicly available via dbGaP (https://www.ncbi.nlm.nih.gov/gap/) including AABC under accession code phs000851.v1.p1, ONCO under accession code phs001265.v1.p1, AMBER under accession code phs000669.v1.p1, ROOT under accession code phs000383.v1.p1, and GBHS under accession code phs002387.v1.p1. Data for TCGA is available via https://portal.gdc.cancer.gov/. The remaining data are available within the Article, Supplementary Information or Source Data file.

## Code availability

The codes and summary statistics of the top 10000+ variants are stored on github (https://github.com/gmgaous/Cross-ancestry-GWAS-for-breast-cancer). The remaining data are available within the Article, Supplementary Information or available from the authors upon request.
