# Supplementary material for: Cross-ancestry GWAS meta-analysis identifies six breast cancer loci in African and European ancestry women
Source: Nat Commun. 2021 Jul 7;12:4198. doi: 10.1038/s41467-021-24327-x (PMC8263739; doi:10.1038/s41467-021-24327-x)
Supplement: Supplementary file 1 — Supplemental information [file 41467_2021_24327_MOESM1_ESM.pdf]

Supplementary Materials

Supplementary Authorship

The GBHS Study Team

Institution and name	Position	Email
Korle Bu Teaching Hospital, Accra, Ghana (KBTH):		
Prof. Joe Nat Clegg-Lamptey	**Surgery (PI)**	clegglamptey@hotmail.com
Dr. Florence Dedey	Surgery	flodedey@yahoo.com
Dr. Lawrence Edusei	Pathology	dredusei@yahoo.com
Dr. Verna Vanderpuye	Radiotherapy and Oncology	vanaglat@yahoo.com ; vanaglat@gmail.com
Dr. Joel Yarney	**Radiotherapy and Oncology (PI)**	kodwoahen@gmail.com
Evelyn Tay	Project coordinator	ghanaprs@africaonline.com.gh
Naomi Oyoe Ohene Oti	Oncology Nurse/Cancer Registry Manager	naaoyoe2000@gmail.com
Victoria Okyne	Study manager	ghprojects2004@gmail.com
University of Ghana, Accra, Ghana:		
Prof. Richard Biritwum	Statistician	biritwum@africaonline.com.gh
Dr. Kofi Nyarko	**Epidemiologist (PI)**	konyarko22@yahoo.com
Komfo Anoyke Teaching Hospital, Kumasi, Ghana (KATH):		
Dr. Ernest Adjei	Pathology	ernest24us@yahoo.com
Dr. Francis Aitpillah	Surgery	fraitp@yahoo.com
Dr. Daniel Ansong	**Epidemiologist (Co-PI)**	ansongd@yahoo.com
Dr. Baffour Awuah	**Oncology (PI)**	baff1470awuah@gmail.com
Dr. Joseph Oppong	Surgery	josephkoppong@yahoo.com
Dr. Ernest Osei-Bonsu	Oncology	ernest.bonsu@gmail.com
Dr. Nicholas Titiloye	Pathology	waleht2000@yahoo.com
Isaac Boakye	Study manager	au347kb@yahoo.com
Bernard Arhin	IT	barhinn@gmail.com
Marion Alcpaloo	Head Nurse Breast Care Center	Madejoe8@gmail.com
Peace and Love Hospital, Kumasi, Ghana:		
Dr. Beatrice Addai Wiafe	**Surgery (PI)**	drwiafe@gmail.com
Dr. Seth Wiafe	**Epidemiologist (Co-PI)**	swiafe@llu.edu
Margaret Frempong	Study manager (PLH)	mtfrempong@gmail.com

Emma Abaidoo	Biological specimens/molecular biology (PLH)	emmaabaidoo@yahoo.com
Prince Agyapong	Data management (PLH)	thelastagyapong@gmail.com
University of Edinburgh, Edinburgh, Scotland:		
Dr. Jonine Figueroa	Epidemiologist	jonine.figueroa@ed.ac.uk
U.S. National Cancer Institute, Bethesda, MD		
**Dr. Montserrat Garcia-Closas**	**Epidemiologist (PI)**	montserrat.garcia-closas@nih.gov
Dr. Louise Brinton	Epidemiologist	brinton6610@gmail.com
Dr. Thomas Ahearn	Epidemiologist	thomas.ahearn@nih.gov
Michelle Brotzman	Study manager (formerly at Westat)	michelle.brotzman@nih.gov
Westat, Inc., MD, USA		
Ann Truelove	Study manager, Westat	AnnTruelove@westat.com

Supplementary Table S1: Consortia contributing to the meta-analysis

Consortium/Study	Total	Controls	Cases	ER positive	ER negative	Genotyping platform
**African ancestry**						
AABC	5727	2720	3007	1518	987	IlluminaHuman1M-Duo BeadChip
BCAC (OncoArray)	3677	1406	2271	1130	613	Illumina Infinium OncoArray-500K BeadChip
AMBER	3815	2408	1407	952	385	Illumina Human Exome Beadchip v1.1 Mega array
Ghana Study	2529	1630	899	296	277	Infinium Global Screening Array-24
ROOT	3686	2029	1657	403	374	Illumina HumanOmni2.5-8v1 array
Total	19434	10193	9241	4299	2636	
**European ancestry**						
BCAC GWAS	32498	17588	14910	4226	4480	Illumina 370K/550K/610K/670K/1.2M, Affymetrix 5.0/6.0
BCAC iCOGs	89677	42892	46785	27078	7333	Illumina iSelect Genotyping Array
BCAC Oncoarray	106776	45494	61282	38197	9655	Illumina Infinium OncoArray-500K BeadChip
Total	228951	105974	122977	69501	21468	
**ROOT Consortium contributing studies**						
NBCS - Nigerian Breast Cancer Study	1335	624	711	42	99	
BNCS - Barbados National Cancer Study	321	229	92			
RVGBC - Racial Variability in Genotypic Determinants of Breast Cancer Study	402	257	145	27	25	
CCPS - Chicago Cancer Prone Study	781	387	394	171	140	
BBCS - Baltimore Breast Cancer Study	197	102	95	45	44	
SCCS - Southern Community Cohort Study	650	430	220	118	66	
**AABC Consortium contributing studies**						
MEC - Multiethnic Cohort Study	1667	976	691	407	176	
CARE - Women's Contraceptive and Reproductive Experiences Study, Los Angeles component	568	212	356	183	129	
WCHS - Women's Circle of Health Study	497	236	261	131	80	
SFBCS - San Francisco Bay Area Breast Cancer Study	382	218	164	84	50	
NC-BCFR - Northern California site of the Breast Cancer Family Registry	470	50	420	218	121	
CBCS - Carolina Breast Cancer Study	1224	589	635	272	317	
PLCO - Prostate, Lungs, Colorectal and Ovarian Cancer Screening Trial Cohort	172	116	56	14	6	
NBHS - Nashville Breast Health Study	485	181	304	143	65	
WFBC - Wake Forest University Breast Cancer Study	262	142	120	66	43	
**AMBER Consortium**						
BWHS - Black Women's Health Study	2466	2154	312	206	64	
CBCS - Carolina Breast Cancer Study	607	1	606	407	186	
WCHS - Women's Circle of Health Study	742	253	489	339	135	
**BCAC (OncoArray) African ancestry**						
2SISTER - The Two Sister Study	45	0	45	16	28	
MEC - Multiethnic Cohort Study	1329	700	629	138	413	
CBCS - Carolina Breast Cancer Study	975	64	911	299	504	
WAABCS - West African Ancestry Breast Cancer Study	622	309	313	63	21	
NC-BCFR, The Northern California Breast Cancer Family Registry	80	1	79	28	40	
NBHS - The Nashville Breast Health Study	146	59	87	40	17	

PLCO - Prostate, Lungs, Colorectal and Ovarian Cancer Screening Trial Cohort	93	68	25	2	13
SISTER - The Sister Study	319	166	153	27	94
USRT - The U.S. Radiologic Technologists	68	39	29		

Table S2. Association results of 6 loci in each study of African ancestry

						OVERALL			ER Negative		
**SNP**	**Locus**	**Position**	**Test**	**Other**	**Dataset**	**TAF**	**OR(95% CI)**	**P value**	**TAF**	**OR (95% CI)**	**P value**
rs17024629	1p13.3	110179756	T	C	All	0.13	0.88 (0.83 - 0.95)	0.00052	0.13	0.83 (0.74 - 0.92)	0.00064
					AMBER	0.13	0.83 (0.67 - 1.02)	0.082	0.13	0.69 (0.48 - 0.99)	0.044
					ROOT	0.13	0.92 (0.80 - 1.06)	0.27	0.13	0.92 (0.72 - 1.18)	0.53
					BCAC	0.14	0.86 (0.74 - 0.99)	0.033	0.14	0.81 (0.65 - 1.01)	0.059
					AABC	0.12	0.92 (0.82 - 1.03)	0.16	0.13	0.86 (0.72 - 1.02)	0.085
					Ghana	0.11	0.84 (0.68 - 1.04)	0.11	0.11	0.73 (0.51 - 1.03)	0.065
rs67931591	1q41	215330292	G	GCTGAGG-CAGGAGA	All	0.28	0.95 (0.90 - 1.00)	0.034	0.29	0.92 (0.85 - 0.99)	0.024
					AMBER	0.29	1.01 (0.87 - 1.17)	0.91	0.29	0.91 (0.71 - 1.17)	0.47
					ROOT	0.28	0.89 (0.80 - 1.00)	0.043	0.28	0.81 (0.68 - 0.97)	0.025
					BCAC	0.29	0.98 (0.89 - 1.09)	0.72	0.29	0.97 (0.83 - 1.13)	0.67
					AABC	0.3	0.94 (0.86 - 1.02)	0.12	0.31	0.93 (0.83 - 1.05)	0.24
					Ghana	0.21	0.96 (0.83 - 1.11)	0.58	0.21	0.93 (0.74 - 1.18)	0.57
rs2522057	5q31.1	131801947	C	G	All	0.77	0.92 (0.86 - 0.98)	0.0084	0.87	0.93 (0.85 - 1.03)	0.18
					AMBER	0.85	0.91 (0.75 - 1.10)	0.35	0.85	1.07 (0.77 - 1.46)	0.7
					ROOT	0.88	0.93 (0.80 - 1.08)	0.34	0.88	0.97 (0.76 - 1.24)	0.81
					BCAC	0.86	0.92 (0.81 - 1.06)	0.27	0.86	0.95 (0.78 - 1.17)	0.65
					AABC	0.85	0.92 (0.82 - 1.02)	0.11	0.85	0.93 (0.79 - 1.09)	0.34
					Ghana	0.9	0.88 (0.71 - 1.07)	0.19	0.89	0.78 (0.58 - 1.05)	0.11
rs1637365	7q11.23	74359358	T	C	All	0.56	1.06 (1.01 - 1.12)	0.024	0.61	1.15 (1.06 - 1.25)	0.00069
					AMBER	0.59	0.95 (0.82 - 1.11)	0.52	0.59	0.87 (0.69 - 1.11)	0.27
					ROOT	0.64	1.05 (0.94 - 1.17)	0.42	0.64	1.05 (0.87 - 1.28)	0.59
					BCAC	0.61	1.06 (0.95 - 1.18)	0.29	0.61	1.19 (1.01 - 1.40)	0.04
					AABC	0.56	1.12 (1.01 - 1.24)	0.031	0.56	1.20 (1.04 - 1.39)	0.012
					Ghana	0.69	1.09 (0.95 - 1.26)	0.23	0.69	1.45 (1.14 - 1.84)	0.0015
rs60381548	15q24.2	75728474	CA	C	All	0.49	0.93 (0.89 - 0.97)	0.0016	0.51	0.99 (0.92 - 1.06)	0.78
					AMBER	0.48	0.92 (0.80 - 1.05)	0.21	0.48	1.03 (0.83 - 1.29)	0.77
					ROOT	0.50	0.97 (0.88 - 1.07)	0.57	0.50	1.14 (0.97 - 1.35)	0.12
					BCAC	0.52	0.92 (0.83 - 1.01)	0.068	0.52	1.01 (0.88 - 1.17)	0.86
					AABC	0.49	0.93 (0.86 - 1.01)	0.078	0.49	0.93 (0.84 - 1.04)	0.24
					Ghana	0.45	0.90 (0.80 - 1.02)	0.11	0.45	0.90 (0.74 - 1.09)	0.29
rs1869959	15q24.1	75147332	A	C	All	0.57	0.95 (0.91 - 1.00)	0.043	0.59	0.96 (0.89 - 1.03)	0.22
					AMBER	0.59	1.07 (0.93 - 1.24)	0.32	0.59	0.95 (0.76 - 1.19)	0.68
					ROOT	0.6	0.98 (0.89 - 1.08)	0.63	0.6	0.94 (0.79 - 1.11)	0.44
					BCAC	0.59	1.00 (0.91 - 1.10)	0.99	0.59	1.02 (0.88 - 1.18)	0.77
					AABC	0.59	0.92 (0.85 - 0.99)	0.03	0.59	0.93 (0.83 - 1.04)	0.18
					Ghana	0.59	1.08 (0.95 - 1.22)	0.23	0.59	1.22 (1.00 - 1.49)	0.044
rs181337095	15q26.3	100907094	A	G	All	0.64	1.06 (1.01 - 1.12)	0.017	0.69	1.07 (0.99 - 1.16)	0.067
					AMBER	0.68	0.96 (0.83 - 1.12)	0.64	0.68	0.88 (0.69 - 1.14)	0.34
					ROOT	0.67	1.01 (0.91 - 1.12)	0.81	0.67	0.97 (0.81 - 1.16)	0.74
					BCAC	0.69	1.07 (0.96 - 1.19)	0.22	0.69	1.06 (0.90 - 1.23)	0.5
					AABC	0.7	1.11 (1.02 - 1.22)	0.015	0.7	1.20 (1.06 - 1.37)	0.003
					Ghana	0.68	1.10 (0.96 - 1.26)	0.17	0.68	1.04 (0.85 - 1.29)	0.69

Abbreviation: TAF, Test allele frequency; SNP, single nucleotide polymorphism; OR, odds ratio; CI, confidence intervals.

Table S3. Association results of novel loci in each BCAC datasets in Europeans

						OVERALL			ER NEGATIVE		
**SNP**	**Locus**	**Position**	**Test**	**Other**	**Dataset**	**TAF**	**OR (95% CI)**	**P value**	**TAF**	**OR (95% CI)**	**P value**
rs17024629	1p13.3	110179756	T	C	Meta	0.16	0.96 (0.94 - 0.98)	1.2E-06	0.16	0.96 (0.93 - 0.99)	0.02
					GWAS	0.16	0.91 (0.87 - 0.98)	0.00014	0.16	0.91 (0.85 - 0.98)	0.014
					iCOGS	0.16	0.95 (0.93 - 0.98)	0.00033	0.16	0.96 (0.91 - 1.02)	0.18
					Oncoarray	0.17	0.98 (0.95 - 1.00)	0.72	0.16	0.98 (0.94 - 1.03)	0.44
rs67931591	1q41	215330292	G	GCTGAGG-CAGGAGA	Meta	0.68	0.98 (0.96 - 0.99)	0.00039	0.68	0.94 (0.92 - 0.96)	4.6E-07
					GWAS	0.68	0.95 (0.91 - 0.99)	0.01	0.68	0.85 (0.79 - 0.91)	3.9E-06
					iCOGS	0.68	0.99 (0.97 - 1.01)	0.55	0.68	0.98 (0.95 - 1.02)	0.44
					Oncoarray	0.68	0.97 (0.95 - 0.99)	0.00083	0.68	0.93 (0.90 - 0.96)	0.00033
rs2522057	5q31.1	131801947	C	G	Meta	0.58	0.97 (0.96 - 0.98)	9.3E-08	0.58	0.99 (0.96 - 1.01)	0.22
					GWAS	0.59	0.95 (0.92 - 0.98)	0.0042	0.59	0.96 (0.91 - 1.01)	0.15
					iCOGS	0.59	0.97 (0.95 - 0.99)	0.0022	0.59	1.00 (0.96 - 1.03)	0.81
					Oncoarray	0.59	0.97 (0.95 - 0.99)	0.00052	0.59	0.99 (0.96 - 1.02)	0.48
rs1637365	7q11.23	74359358	T	C	Meta	0.28	1.04 (1.02 - 1.05)	3.3E-06	0.28	1.07 (1.04 - 1.10)	9.0E-07
					GWAS	0.28	1.06 (1.00 - 1.12)	0.033	0.28	1.10 (1.00 - 1.22)	0.056
					iCOGS	0.29	1.03 (1.00 - 1.05)	0.027	0.28	1.05 (1.00 - 1.10)	0.06
					Oncoarray	0.28	1.04 (1.02 - 1.06)	0.00026	0.28	1.08 (1.04 - 1.12)	1.70E-05
rs60381548	15q24.2	75728474	CA	C	Meta	0.25	0.96 (0.95 - 0.98)	4.0E-07	0.25	0.95 (0.92 - 0.97)	8.60E-05
					GWAS	0.24	0.98 (0.94 - 1.03)	0.41	0.25	0.94 (0.87 - 1.01)	0.11
					iCOGS	0.24	0.96 (0.94 - 0.98)	0.00091	0.24	0.95 (0.91 - 1.00)	0.034
					Oncoarray	0.25	0.96 (0.94 - 0.98)	0.00011	0.25	0.95 (0.91 - 0.98)	0.0037
rs1869959	15q24.1	75147332	A	C	Meta	0.7	1.04 (1.02 - 1.05)	3.6E-07	0.7	1.04 (1.01 - 1.06)	0.005
					GWAS	0.71	1.02 (0.98 - 1.06)	0.36	0.7	0.99 (0.93 - 1.04)	0.61
					iCOGS	0.71	1.02 (1.00 - 1.05)	0.025	0.71	1.03 (0.99 - 1.07)	0.18
					Oncoarray	0.7	1.05 (1.03 - 1.07)	1.2E-06	0.7	1.06 (1.02 - 1.10)	0.0013
rs181337095	15q26.3	100907094	A	G	Meta	0.87	1.05 (1.03 - 1.07)	3.4E-07	0.87	1.06 (1.02 - 1.10)	0.0016
					GWAS	0.88	1.03 (0.98 - 1.10)	0.25	0.88	1.03 (0.94 - 1.13)	0.51
					iCOGS	0.86	1.08 (1.05 - 1.12)	1.6E-06	0.86	1.10 (1.03 - 1.18)	0.0038
					Oncoarray	0.87	1.04 (1.01 - 1.07)	0.011	0.87	1.05 (0.99 - 1.10)	0.08

Abbreviation: TAF, Test allele frequency; SNP, single nucleotide polymorphism; OR, odds ratio; CI, confidence intervals.

Table S4. Pleiotropy of primary hits in the GWAS Catalog

SNP	SNP in LD	rsq	Effect allele	Other allele	Disease/trait	Beta	P	Study	Journal	PMID	Source of beta
rs2522057 at 5q31.1	rs2248116	0.94	C	A	Mosquito bite size, itch intensity, attractiveness to mosquitoes	0.034	3.5 X 10-21	GWAS of self-reported mosquito bite size, itch intensity and attractiveness to mosquitoes implicates immune-related predisposition loci.	Hum Mol Genet	28199695[1]	
					Eosinophil % of WBCs, sum of eosinophil and basophol counts, neutrophil % of granulocytes	0.073	6.61 X 10-95	The Allelic Landscape of Human Blood Cell Trait Variation and Links to Common Complex Disease.	Cell	27863252[2]	
	rs6866614	0.42	G	A	Asthma	0.072	4.4 X 10-23	Genetic Architectures of Childhood- and Adult-Onset Asthma Are Partly Distinct.	Am J Hum Genet	30929738[3]	
					Mosquito bite size, itch intensity, attractiveness to mosquitoes	-0.048	5.5 X 10-36	GWAS of self-reported mosquito bite size, itch intensity and attractiveness to mosquitoes implicates immune-related predisposition loci.	Hum Mol Genet	28199695[1]	
rs1869959 at 15q24.1	rs11072518	0.59	T	C	Mean arterial blood pressure x alcohol consumption interaction	0.29	9.12 X 10-31	Novel genetic associations for blood pressure identified via gene-alcohol interaction in up to 570K individuals across multiple ancestries	PLOS One	29912962[4]	
	rs2301249	0.53	T	C	Systolic blood pressure x alcohol consumption (light vs heavy) interaction (2df test)	0.44	4.9 x 10-14	Novel genetic associations for blood pressure identified via gene-alcohol interaction in up to 570K individuals across multiple ancestries.	PLOS One	29912962[4]	Cross ancestry meta-analysis
	rs936226	0.56	T	C	Diastolic blood pressure	-0.549	3.06 X 10-16	The genetics of blood pressure regulation and its target organs from association studies in 342,415 individuals.	Nat Genet	27618452[5]	
	rs2472299	0.4	G	A	Coronary heart disease		3.09 x 10-6	A genome-wide association study in Europeans and South Asians identifies five new loci for coronary artery disease.	Nat Genet	21378988[6]	
	rs1350193	0.49	C	G	Diastolic blood pressure x alcohol consumption (light vs heavy) interaction (2df test)	-0.27	2.69 X 10-13	Novel genetic associations for blood pressure identified via gene-alcohol interaction in up to 570K individuals across multiple ancestries	PLOS One	29912962[4]	Cross ancestry meta-analysis
	rs10628234	0.65	CACA	CA	Diastolic blood pressure x smoking status (ever vs never) interaction (2df test)	0.32	1.57 x 10-24	A Large-Scale Multi-ancestry Genome-wide Study Accounting for Smoking Behavior Identifies Multiple Significant Loci for Blood Pressure.	Am J Hum Genet	29455858[6]	Trans-ancestry analysis
	rs6495127	0.76	T		Self reported educational attainment	0.009	5.142 x 10-9	Gene discovery and polygenic prediction from a genome-wide association study of educational attainment in 1.1 million individuals.	Nat Genet	30038396[7]	
	rs1543927	0.42	T	C	Medication use (beta blocking agents)	0.05	2.2 x 10-8	Genome-wide association study of medication-use and associated disease in the UK Biobank.	Nat Commun	31015401[8]	
rs60381548 at 15q24.2	rs8027365	0.83	C	A	Breast cancer	0.038	1 x10-7	Association analysis identifies 65 new breast cancer risk loci.	Nature	29059683[9]	
	rs4886707	0.83	T	C	Body height	0.06	8 x 10-8	A genome-wide association study in 19 633 Japanese subjects identified LHX3-QSOX2 and IGF1 as adult height loci.	Hum Mol Genet	20189936[10]	
	rs4886707	0.83	T	C	Body height		1 x 10-7	Evaluation and application of summary statistic imputation to discover new height-associated loci.	PLOS Genet	29782485[11]	
	rs8024244	0.82	A	G	Height		1 x 10-51	Leveraging Polygenic Functional Enrichment to Improve GWAS Power	Am J Hum Genet	30595370[12]	
	rs4886699	0.77	C	A	Estimated glomerular filtration rate	0.0031	5 x 10-19	A catalog of genetic loci associated with kidney function from analyses of a million individuals.	Nat Genet	31152163[13]	
	rs7166281	0.4	G	T	type II diabetes mellitus		1 X 10-7	Leveraging Polygenic Functional Enrichment to Improve GWAS Power	Am J Hum Genet	30595370[14]	

Table S5A. Association between novel SNPs and candidate genes in eQTL analysis of breast tumors in TCGA*

					Beta			P value		
Novel SNP	test/other alleles	geneSymbol	gene_start	gene_end	African	European	Meta-analysis	African	European	Meta-analysis
rs17024629	T/C	GSTM4	110198703	110208118	0.28	0.29	0.29	0.07	1.49E-07	2.12E-08
		GSTM2	110210644	110252171	0.48	0.49	0.49	0.15	4.65E-06	1.30E-06
		GSTM1	110230436	110251661	1.33	1.45	1.43	0.06	1.38E-07	1.70E-08
rs67931591	G/GCTGAGGCAGGAGA	PTPN14	214522039	214725792	0.17	0.10	0.11	0.15	0.059	0.0205
rs2522057	C/G	IRF1	131817301	131826490	0.18	0.19	0.19	0.28	0.000148	7.39E-05
		CCNI2	132083137	132089856	0.01	0.33	0.31	0.99	0.000179	2.87E-04
rs1637366**	C/G	STAG3L2	74112305	74306731	0.09	0.22	0.20	0.56	0.000358	0.000368
rs1869959	C/A	ULK3	75128457	75135687	-0.01	-0.10	-0.08	0.86	8.47E-04	0.001458
		MPI	75182346	75191798	0.00	0.11	0.09	0.98	3.35E-05	0.000108
rs60381548	CA/C	SIN3A	75661720	75748183	0.16	0.05	0.07	0.00	2.94E-02	5.29E-04
		PTPN9	75759462	75871630	0.13	0.05	0.07	0.02	2.22E-02	2.08E-03
		SNUPN	75890424	75918810	0.06	0.11	0.10	0.27	2.35E-05	1.68E-05
		SNX33	75940247	75954642	0.09	0.05	0.06	0.07	3.24E-02	5.98E-03

* Multivariable linear model adjusted for age, ancestry, CNV, batch effect and molecular subtype of breast cancer.
** rs1637366 is in strong LD with rs1637365 (r^2=1 for European population and r^2=0.69 in African population)

Table S5B. Association between novel SNPs and candidate genes in eQTL analysis of normal breast tissues in GTEx*

Novel SNP	Loci	geneSymbol	gene_start	gene_end	p-value
rs2522057	5q31	SLC22A5	131,705,444	131,731,306	9.27E-07
rs1869959	15q24.1	ULK3	75,128,457	75,135,687	7.59E-11

* The Genotype-Tissue Expression (GTEx) pilot analysis: Multitissue gene regulation in humans. The GTEx Consortium. Science 2015; 348(6235): 648-660

Supplementary Table S6: Functional annotation of lead SNPs and others in high LD using Haploreg

chr	pos (hg38)	LD (r²)	LD (D')	variant	Ref	Alt	AFR freq	AMR freq	ASN freq	EUR freq	Promoter histone marks	Enhancer histone marks	DNAse	Proteins bound	Motifs changed	NHGRI/EBI GWAS hits	GRASP QTL hits	Selected eQTL hits	GENCODE genes	dbSNP func annot
**EUROPEANS**																				
**rs2522057**																				
5	132435113	0.86	-0.94	rs2188962	C	T	0.05	0.29	0	0.38		22 tissues	6 tissues	HNF4A,HNF4G	6 altered motifs	3 hits	324 hits	45 hits	C5orf56	intronic
5	132442760	0.9	-0.96	rs17622378	A	G	0.05	0.28	0	0.38		9 tissues			4 altered motifs			45 hits	C5orf56	intronic
5	132448701	0.91	-0.97	rs12521868	G	T	0.05	0.28	0	0.38		12 tissues			5 altered motifs	1 hit	5 hits	45 hits	C5orf56	intronic
5	132449992	0.92	-0.97	rs12515180	C	T	0.04	0.28	0	0.38		7 tissues			8 altered motifs			44 hits	C5orf56	intronic
5	132450759	0.91	-0.97	rs146604341	G	GATA	0.04	0.28	0	0.38		10 tissues	SKIN,PLCNT,OVRY	FOXA1,GATA3	14 altered motifs			40 hits	C5orf56	intronic
5	132450903	0.92	-0.97	rs11951091	G	A	0.04	0.28	0	0.37		10 tissues	6 tissues		GATA,Nkx2			45 hits	C5orf56	intronic
5	132451445	0.92	0.97	rs6866614	A	G	0.9	0.72	1	0.63		14 tissues	PLCNT		ZID			44 hits	C5orf56	intronic
5	132457594	0.99	-0.99	rs4705950	C	T	0.05	0.28	0.03	0.37	BLD, THYM	24 tissues	23 tissues	ELF1,POL2	4 altered motifs			43 hits	C5orf56	3'-UTR
5	132460471	0.98	-0.99	rs72797303	T	C	0.04	0.28	0.03	0.37		23 tissues	7 tissues	CTCF	BDP1,ZNF263			38 hits	C5orf56	intronic
5	132461117	1	1	rs2706396	A	C	0.88	0.71	0.97	0.63		16 tissues	GI,GI	POL2	7 altered motifs			39 hits	C5orf56	3'-UTR
5	132462795	1	1	rs2522052	C	T	0.88	0.71	0.97	0.63		18 tissues			5 altered motifs			44 hits	C5orf56	intronic
5	132463320	0.89	1	rs2706403	A	G	0.85	0.7	0.94	0.6		19 tissues	GI,GI,GI		TCF12			34 hits	C5orf56	intronic
5	132464269	0.99	1	rs2706336	T	C	0.88	0.71	0.97	0.63	20 tissues	13 tissues	7 tissues	EBF1	6 altered motifs			39 hits	C5orf56	intronic
5	132465058	1	-1	rs72797306	G	C	0.05	0.28	0.03	0.37	14 tissues	20 tissues	7 tissues		7 altered motifs			38 hits	C5orf56	intronic
5	132466255	1	1	rs2522057	G	C	0.88	0.71	0.97	0.63	4 tissues	22 tissues	10 tissues	GATA1			2 hits	44 hits	C5orf56	intronic
5	132468655	0.98	0.99	rs2248116	C	A	0.87	0.71	0.97	0.63		13 tissues			Hsf		1 hit	45 hits	C5orf56	intronic
5	132475490	0.94	-0.97	rs11741255	G	A	0.05	0.28	0.02	0.37		24 tissues	7 tissues		10 altered motifs			44 hits	C5orf56	intronic

Supplementary Table S6: Functional annotation of lead SNPs and others in high LD using Haploreg (Contd)

chr	pos (hg38)	LD (r²)	LD (D')	variant	Ref	Alt	AFR freq	AMR freq	ASN freq	EUR freq	Promoter histone marks	Enhancer histone marks	DNAse	Proteins bound	Motifs changed	GRASP QTL hits	Selected eQTL hits	GENCODE genes	dbSNP func annot
**EUROPEANS**																			
1	109626568	0.94	0.99	rs865774	C	T	0.12	0.13	0.03	0.16		5 tissues			VDR	8 hits	27 hits	AMPD2	intronic
1	109628903	0.94	0.99	rs560674	C	G	0.12	0.13	0.03	0.16		6 tissues			VDR		20 hits	RP5-1160K1.6	intronic
1	109631153	0.95	1	rs568686	G	T	0.11	0.13	0.03	0.16		8 tissues				4 hits	25 hits	AMPD2	3'-UTR
1	109631583	0.95	1	rs538388	C	T	0.11	0.13	0.03	0.16		7 tissues	THYM		HNF4,VDR	1 hit	20 hits	AMPD2	3'-UTR
1	109636928	1	1	rs17024628	G	A	0.05	0.12	0	0.16	CRVX, LIV	13 tissues	CRVX	CTCF, NFYB	TEF-1		18 hits	4.9kb 3' of AMPD2	
1	109637134	1	1	rs17024629	C	T	0.13	0.13	0	0.16	LIV	7 tissues	SKIN				23 hits	5.1kb 3' of AMPD2	
1	109637740	1	1	rs58434753	AT	A	0.13	0.13	0	0.16					10 altered motifs		18 hits	5.7kb 3' of AMPD2	
		1	1	rs148898127	7-mer	A	0.13	0.13	0	0.16					4 altered motifs		12 hits	5.8kb 3' of AMPD2	
1	109640399	1	1	rs3850616	G	T	0.28	0.14	0	0.16	BLD, SKIN	13 tissues	48 tissues	11 bound proteins	CTCF	4 hits	19 hits	8.3kb 3' of AMPD2	
1	109650432	0.94	0.97	rs604337	G	A	0.17	0.13	0.03	0.16		8 tissues			6 altered motifs		19 hits	5.6kb 5' of GSTM4	
1	109653057	0.92	-0.96	rs669426	G	A	0.85	0.88	0.97	0.84		GI, CRVX			HP1-site-factor		20 hits	3kb 5' of GSTM4	
1	109653885	0.9	-0.96	rs2781815	T	C	0.83	0.88	0.97	0.84		GI, CRVX					21 hits	2.2kb 5' of GSTM4	
1	109653977	0.89	-0.96	rs2781814	C	T	0.83	0.88	0.97	0.84		GI, CRVX			4 altered motifs		18 hits	2.1kb 5' of GSTM4	
1	109654074	0.86	-0.96	rs201520834	T	TC-GAA	0.78	0.87	0.96	0.84		GI, CRVX			8 altered motifs		17 hits	2kb 5' of GSTM4	
**rs1869959**																			
15	74849699	0.8	0.93	rs4886613	G	A	0.3	0.71	0.7	0.67					Cphx,HNF4		38 hits	SCAMP2	intronic
15	74852757	0.86	0.99	rs936230	T	C	0.54	0.73	0.71	0.68		SKIN	MUS,LIV		Irf,Smad4	2 hits	53 hits	SCAMP2	intronic
15	74854991	1	1	rs1869959	A	C	0.52	0.71	0.7	0.65		MUS, GI			9 altered motifs		51 hits	SCAMP2	intronic
15	74856432	0.85	0.98	rs7180432	A	G	0.32	0.72	0.7	0.68		MUS, GI, BLD			PLZF		41 hits	SCAMP2	intronic
**rs60381548**																			
15	75436133	1	1	rs60381548	C	CA									13 altered motifs			SIN3A	intronic
**rs67931591**																			
1	215156949	1	1	rs67931591	14-mer	G									9 altered motifs			KCNK2	intronic
**rs1637365**																			
7	74944369	1	1	rs1637365	C	T	0.58	0.17	0.08	0.22							35 hits	20kb 5' of GATSL1	
**rs181337095**																			
15	100366889	1	1	rs181337095	G	A	0.21	0.28	0.28	0.3					5 altered motifs			6kb 5' of RP11-168G16.2	

Supplementary Table S6: Functional annotation of lead SNPs and others in high LD using Haploreg (Contd)

chr	pos (hg38)	LD	LD	variant	Ref	Alt	AFR	AMR	ASN	EUR	Promoter	Enhancer	DNAse	Proteins	Motifs	GRASP QTL	Selected eQTL	GENCODE	dbSNP
		**(r²)**	**(D')**				**freq**	**freq**	**freq**	**freq**	**histone marks**	**histone marks**		**bound**	**changed**	**hits**	**hits**	**genes**	**func annot**
		**(r²)**	**(D')**				**freq**	**freq**	**freq**	**freq**	**histone marks**	**histone marks**		**bound**	**changed**	**hits**	**hits**	**genes**	**func annot**
**AFRICANS**																			
**rs2522057**																			
5	132461117	0.98	1	rs2706396	A	C	0.88	0.71	0.97	0.63		16 tissues	GI,GI	POL2	7 altered motifs		39 hits	C5orf56	3'-UTR
5	132462795	0.98	1	rs2522052	C	T	0.88	0.71	0.97	0.63		18 tissues			5 altered motifs		44 hits	C5orf56	intronic
5	132464269	0.98	1	rs2706336	T	C	0.88	0.71	0.97	0.63	20 tissues	13 tissues	7 tissues	EBF1	6 altered motifs		39 hits	C5orf56	intronic
5	132466255	1	1	rs2522057	G	C	0.88	0.71	0.97	0.63	4 tissues	22 tissues	10 tissues	GATA1		2 hits	44 hits	C5orf56	intronic
5	132468655	0.91	1	rs2248116	C	A	0.87	0.71	0.97	0.63		13 tissues			Hsf	1 hit	45 hits	C5orf56	intronic
**rs17024629**																			
1	109637134	1	1	rs17024629	C	T	0.13	0.13	0	0.16		9 tissues	SKIN				23 hits	5.1kb 3' of AMPD2	
1	109637740	1	1	rs58434753	AT	A	0.13	0.13	0	0.16					10 altered motifs		18 hits	5.7kb 3' of AMPD2	
		0.93	0.96	rs148898127	7-mer	A	0.13	0.13	0	0.16					4 altered motifs		12 hits	5.8kb 3' of AMPD2	
**rs1869959**																			
15	74852757	0.8	0.92	rs936230	T	C	0.54	0.73	0.71	0.68		SKIN	MUS,LIV		Irf,Smad4	2 hits	53 hits	SCAMP2	intronic
15	74854991	1	1	rs1869959	A	C	0.52	0.71	0.7	0.65		MUS, GI			9 altered motifs		51 hits	SCAMP2	intronic
**rs60381548**																			
15	75436133	1	1	rs60381548	C	CA									13 altered motifs			SIN3A	intronic
**rs67931591**																			
1	215156949	1	1	rs67931591	14-mer	G									9 altered motifs			KCNK2	intronic
**rs181337095**																			
15	100366889	1	1	rs181337095	G	A	0.21	0.28	0.28	0.3					5 altered motifs			6kb 5' of RP11-168G16.2	

Supplementary table 7A: Detailed functional annotation for novel loci and correlated SNPs (r^2 >0.8) - Europeans

SNP	Roadmap<br>25-state model using 12 imputed marks	ENCODE<br>25-state model using 12 imputed marks	Roadmap<br>H3K4me1	Roadmap<br>H3K4me3	Roadmap<br>H3K27ac	Roadmap<br>H3K9ac	ENCODE Enhancers H3K4me1 and H3K27ac	ENCODE Promoters H3K4me3 and H3K9ac	DNase
**rs2522057**	**22_PromP**<br>(ESDR.H9.NEUR.PROG; ESDR.CD56.MESO; BRN.CRTX.DR.NRSPHR; BRN.GRM.MTRX)<br><br>**19_DNase**<br>(ESDR.H9.NEUR)<br><br>**17_EnhW2**<br>ESDR.H1.BMP4.TROP; SKIN.PEN.FRSK.FIB.02; SKIN.PEN.FRSK.MEL.03; OVRY; PANC.ISLT)<br><br>**15_EnhAF**<br>ESDR.H1.MSC; FAT.ADIP.DR.MSC; FAT.MSC.DR.ADIP; SKIN.PEN.FRSK.FIB.01; SKIN.PEN.FRSK.MEL.01; HRT.ATR.R; HRT.VENT.L; HRT.VNT.R; GI.STMC.GAST; PANC; LNG)<br><br>**13_EnhA1**<br>BLD.PER.MONUC.PC; BLD.CD4.CD25I.CD127.TMEMPC; BLD.CD3.CPC; BLDBLD.CD4.CD25M.CD45RA.NPC; BLD.CD4.CD25M.IL17P.PL.TPC; BLD.CD8.NPC; BLD.CD34.PC; STRM.MRW.MSC; THYM; FAT.ADIP.NUC; GI.DUO.SM.MUS; GI.S.INT; GI.CLN.SIG; GI.RECT.MUC.31; LIV.ADLT)<br><br>3_PromD1 (BLD.CD3.PPC; BLD.CD4.CD25.CD127M.TREGPC; BLD.CD4.CD25M.TPC;	**2_PromU**<br>(BLD.GM12878; BLD.CD14.MONO); 13_EnhA1 (LIV.ADLT; BLD.DND41.CNCR; MUS.HSMMT; BLD.K562.CNCR)<br><br>**14_EnhA2**<br><br>(LIV.HEPG2.CNCR; BRST.HMEC; MUS.HSMM; VAS.HUVEC; SKIN.NHEK; BONE.OSTEO); 15_EnhAF (LNG.A549.ETOH002.CNCR; BRN.NHA; SKIN.NHDFAD; LNG.NHLF); 17_EnhW2	LNG.IMR90; ESC.WA7; ESC.H9; ESC.I3; ESC.HUES6; ESC.HUES48; ESC.H1; IPSC.20B; IPSC.15b; IPSC.DF.19.11; ESDR.CD56.MESO; ESDR.H1.BMP4.TROP; ESDR.H1.MSC; BLD.PER.MONUC.PC; BLD.CD3.PPC; BLD.CD4.CD25I.CD127.TMEMPC; BLD.CD3.CPC; BLD.CD4.CD25.CD127M.TREGPC; BLD.CD4.CD25M.TPC; BLD.CD4.CD25M.CD45RA.NPC; BLD.CD4.CD25M.IL17M.PL.TPC; BLD.CD4.CD25M.IL17P.PL.TPC; BLD.CD4.CD25M.CD45RO.MPC; BLD.CD4.MPC; BLD.CD8.MPC; BLD.CD4.NPC; BLD.CD8.NPC; BLD.CD14.PC; BLD.CD19.CPC; BLD.CD34.PC; BLD.MOB.CD34.PC.M; BLD.MOB.CD34.PC.F; BLD.CD34.CC; BLD.CD19.PPC; BLD.CD56.PC; BLD.CD15.PC; STRM.MRW.MSC; STRM.CHON.MRW.DR.MSC; MUS.SAT; SKIN.PEN.FRSK.FIB.01; SKIN.PEN.FRSK.FIB.02; SKIN.PEN.FRSK.MEL.01; SKIN.PEN.FRSK.MEL.03; SKIN.PEN.FRSK.KER.02; SKIN.PEN.FRSK.KER.03; BRST.HMEC.35; BRST.MYO; BRN.GANGEM.DR.NRSPHR; BRN.CRTX.DR.NRSPHR; THYM; THYM.FET; BRN.HIPP.MID; BRN.SUB.NIG; BRN.ANT.CAUD;	BLD.PER.MONUC.PC; BLD.CD3.PPC; BLD.CD4.CD25I.CD127.TMEMPC; BLD.CD3.CPC; BLD.CD4.CD25.CD127M.TREGPC; BLD.CD4.CD25M.TPC; BLD.CD4.CD25M.CD45RA.NPC; BLD.CD4.CD25M.IL17M.PL.TPC; BLD.CD4.CD25M.IL17P.PL.TPC; BLD.CD4.CD25M.CD45RO.MPC; BLD.CD4.MPC; BLD.CD8.MPC; BLD.CD4.NPC; BLD.CD8.NPC; BLD.CD14.PC; BLD.CD19.CPC; BLD.CD34.PC; BLD.MOB.CD34.PC.M; BLD.MOB.CD34.PC.F; BLD.CD34.CC;	IPSC.DF.19.11; BLD.PER.MONUC.PC; BLD.CD3.PPC; BLD.CD4.CD25I.CD127.TMEMPC; BLD.CD3.CPC; BLD.CD4.CD25.CD127M.TREGPC; BLD.CD4.CD25M.TPC; BLD.CD4.CD25M.CD45RA.NPC; BLD.CD4.CD25M.IL17M.PL.TPC; BLD.CD4.CD25M.IL17P.PL.TPC; BLD.CD4.CD25M.CD45RO.MPC; BLD.CD4.MPC; BLD.CD8.MPC; BLD.CD4.NPC; BLD.CD8.NPC; BLD.CD14.PC; BLD.CD19.CPC; BLD.CD34.PC; BLD.MOB.CD34.PC.M; BLD.MOB.CD34.PC.F; BLD.CD34.CC; BLD.CD19.PPC; BLD.CD56.PC; BLD.CD15.PC; STRM.MRW.MSC; MUS.SAT; SKIN.PEN.FRSK.FIB.02; SKIN.PEN.FRSK.KER.02; THYM; THYM.FET; BRN.HIPP.MID; BRN.ANT.CAUD; FAT.ADIP.NUC; MUS.SKLT.F; GI.DUO.SM.MUS; GI.CLN.SM.MUS; GI.RECT.SM.MUS; GI.STMC.MUS; GI.CLN.SIG; GI.CLN.MUC;	ESC.HUES6; BLD.PER.MONUC.PC; BLD.CD4.NPC; BLD.CD8.NPC; STRM.MRW.MSC; FAT.MSC.DR.ADIP; MUS.SAT; BRN.ANT.CAUD; BRN.INF.TMP; BRN.ANG.GYR; BRN.DL.PRFRNTL.CRTX; BRN.DL.PRFRNTL.CRTX; FAT.ADIP.NUC; MUS.SKLT.F; MUS.SKLT.M ; GI.CLN.SM.MUS; GI.RECT.SM.MUS; GI.STMC.MUS; GI.CLN.MUC; GI.RECT.MUC.29; GI.RECT.MUC.31; GI.STMC.MUC; GI.DUO.MUC; PANC.ISLT; LIV.ADLT	**H3K4me1**<br><br>LNG.A549.ETOH002.CNCR; BLD.DND41.CNCR; BLD.GM12878; CRVX.HELAS3.CNCR; LIV.HEPG2.CNCR; BRST.HMEC; MUS.HSMM; MUS.HSMMT; VAS.HUVEC; BLD.K562.CNCR; BLD.CD14.MONO; BRN.NHA; SKIN.NHDFAD; SKIN.NHEK; LNG.NHLF; BONE.OSTEO	**H3K4me3**<br><br>BLD.DND41.CNCR; BLD.GM12878; MUS.HSMMT; BLD.CD14.MONO; SKIN.NHEK; BONE.OSTEO.<br><br>**H3K9ac**<br><br>LNG.A549.ETOH0	ROADMAP BLD.CD3.PPC; BLD.CD3.CPC; BLD.MOB.CD34.PC.M; BLD.MOB.CD34.PC.F; BLD.CD19.PPC; BLD.CD56.PC; SKIN.PEN.FRSK.KER.02; THYM.FET;<br><br>ENCODE BLD.GM12878; SKIN.NHEK

	BLD.CD4.CD25M.IL17M.PL.TPC; BLD.CD4.CD25M.CD45RO.MPC; BLD.CD4.MPC; BLD.CD8.MPC; BLD.CD4.NPC; BLD.CD19.CPC; BLD.MOB.CD34.PC.M; BLD.MOB.CD34.PC.F; BLD.CD34.CC; BLD.CD19.PPC; BLD.CD56.PC; BLD.CD15.PC; THYM.FET)<br><br>**2_PromU** (BLD.CD14.PC)<br><br>**9_TxReg** (GI.RECT.MUC.29; GI.DUO.MUC; SPLN)<br><br>**14_EnhA2**<br>STRM.CHON.MRW.DR.MSC; MUS.SAT; SKIN.PEN.FRSK.KER.02; SKIN.PEN.FRSK.KER.03; BRST.HMEC.35; BRST.MYO; BRN.HIPP.MID; BRN.DL.PRFRNTL.CRTX; MUS.SKLT.F; MUS.SKLT.M; MUS.TRNK.FET; MUS.LEG.FET; GI.CLN.SM.MUS; GI.RECT.SM.MUS; GI.STMC.MUS; GI.S.INT.FET; GI.L.INT.FET; GI.CLN.MUC; GI.STMC.MUC; GI.DUO.MUC; KID.FET; LNG.FET; ADRL.GLND.FET; PLCNT.FET)<br><br>**16_EnhW1**<br>BRN.SUB.NIG; BRN.ANT.CAUD; BRN.CING.GYR; BRN.INF.TMP; BRN.ANG.GYR; BRN.FET.M; MUS.PSOAS; GI.STMC.FET; PLCNT.AMN)	(CRVX.HELAS3.CNCR)	BRN.CING.GYR; BRN.DL.PRFRNTL.CRTX; FAT.ADIP.NUC; MUS.PSOAS; MUS.SKLT.F; MUS.TRNK.FET; MUS.LEG.FET; HRT.FET; HRT.ATR.R; HRT.VENT.L; HRT.VNT.R; GI.DUO.SM.MUS; GI.CLN.SM.MUS; GI.RECT.SM.MUS; GI.STMC.MUS; GI.STMC.FET; GI.S.INT.FET; GI.L.INT.FET; GI.S.INT; GI.CLN.SIG; GI.CLN.MUC; GI.RECT.MUC.29; GI.RECT.MUC.31; GI.STMC.MUC; GI.DUO.MUC; GI.ESO; GI.STMC.GAST; PLCNT.AMN; LNG.FET; OVRY; ADRL.GLND.FET; PLCNT.FET; LIV.ADLT; PANC; LNG; SPLN	BLD.CD19.PPC; BLD.CD56.PC; BLD.CD15.PC; STRM.MRW.MSC; MUS.SAT; SKIN.PEN.FRSK.FIB.02; SKIN.PEN.FRSK.KER.02; THYM; THYM.FET; BRN.HIPP.MID; BRN.ANT.CAUD; FAT.ADIP.NUC; MUS.SKLT.F; GI.DUO.SM.MUS; GI.CLN.SM.MUS; GI.RECT.SM.MUS; GI.STMC.MUS; GI.CLN.SIG; GI.CLN.MUC; GI.RECT.MUC.29; GI.RECT.MUC.31; GI.STMC.MUC; GI.DUO.MUC; GI.ESO; LIV.ADLT.	GI.RECT.MUC.29; GI.RECT.MUC.31; GI.STMC.MUC; GI.DUO.MUC; GI.ESO; LIV.ADLT.		**H3K27ac**<br>BLD.DND41.CNCR; BLD.GM12878; MUS.HSMMT; BLD.K562.CNCR; BLD.CD14.MONO; SKIN.NHEK; BONE.OSTEO.	02.CNCR; BLD.DND41.CNCR; BLD.GM12878; CRVX.HELAS3.CNCR; LIV.HEPG2.CNCR; BRST.HMEC; MUS.HSMM; MUS.HSMMT; VAS.HUVEC; BLD.K562.CNCR; BLD.CD14.MONO; BRN.NHA; SKIN.NHDFAD; SKIN.NHEK; LNG.NHLF; BONE.OSTEO	
rs2188962	**13_EnhA1** (LNG.IMR90)<br><br>**17_EnhW2** (IPSC.DF.19.11; ESDR.H1.NEUR.PROG; ESDR.H9.NEUR.PROG; ESDR.H9.NEUR; FAT.MSC.DR.ADIP; MUS.SAT; BRST.HMEC.35; MUS.TRNK.FET; HRT.FET; VAS.AOR; PLCNT.AMN; PANC.ISLT)	**17_EnhW2**<br><br>(LNG.A549.ETOH002.CNCR; CRVX.HELAS3.CNCR; BRST.HMEC; MUS.HSM	LNG.IMR90; ESC.I3; ESC.HUES6; ESC.HUES48; ESC.HUES64; ESC.4STAR; IPSC.20B; PSC.DF.6.9; IPSC.DF.19.11; ESDR.H1.NEUR.PROG; ESDR.H9.NEUR.PROG; ESDR.H9.NEUR; ESDR.CD56.MESO; ESDR.CD56.ECTO; ESDR.CD184.ENDO;	BLD.CD4.CD25I.CD127.TMEMPC; BLD.CD4. BLD.MOB.CD34.PC.M; BLD.MOB.CD34.PC.F; MUS.SKLT.F; GI.DUO.SM.M	LNG.IMR90; ESDR.H1.MSC; BLD.PER.MONUC.PC; BLD.CD3.PPC; BLD.CD4.CD25I.CD127.TMEMPC; BLD.CD4.CD25.CD127M.TREGPC; BLD.CD4.CD25M.TPC; BLD.CD4.CD25M.CD45RA.NPC; BLD.CD4.CD25M.IL17M.PL.T	LNG.IMR90; IPSC.15b; ESDR.H1.NEUR.PROG; ESDR.H1.BMP4.TROP; BLD.PER.MONUC.PC; BLD.CD4.NPC; BRN.SUB.NIG;	**H3K4me1**<br><br>BLD.DND41.CNCR; BLD.GM12878; LIV.HEPG2.CNCR; BRST.HMEC;	**H3K4me3**<br><br>BLD.DND41.CNCR; LIV.HEPG2.CNCR.	ROADMAP GI.S.INT.FET; GI.L.INT.FET; GI.S.INT; GI.STMC.GAST<br><br>ENCODE

	**16_EnhW1** (SKIN.PEN.FRSK.KER.03)<br><br>**15_EnhAF** (ESDR.H1.BMP4.TROP; SKIN.PEN.FRSK.KER.02; BRST.MYO; MUS.PSOAS; GI.ESO; GI.STMC.GAST; KID.FET; LNG.FET; OVRY; ADRL.GLND.FET;<br><br>**14_EnhA2** (ESDR.H1.MSC)<br><br>**12_TxEnhW** (BLD.PER.MONUC.PC; BLD.CD3.CPC; BLD.CD4.CD25.CD127M.TREGPC; BLD.CD34.PC; BLD.CD34.CC; SKIN.PEN.FRSK.FIB.01; SKIN.PEN.FRSK.FIB.02; SKIN.PEN.FRSK.MEL.01; THYM; BRN.ANT.CAUD; BRN.CING.GYR; BRN.INF.TMP; BRN.ANG.GYR; BRN.DL.PRFRNTL.CRTX; MUS.LEG.FET)<br><br>**10_TxEnhW** (BLD.CD3.PPC; BLD.CD4.CD25I.CD127.TMEMPC; BLD.CD4.CD25M.TPC; BLD.CD4.CD25M.CD45RA.NPC; BLD.CD4.CD25M.IL17M.PL.TPC; BLD.CD4.CD25M.IL17P.PL.TPC; BLD.CD4.CD25M.CD45RO.MPC; BLD.CD4.MPC; BLD.CD8.MPC; BLD.CD4.NPC; BLD.CD8.NPC; BLD.CD14.PC; BLD.CD19.CPC; BLD.MOB.CD34.PC.M; BLD.MOB.CD34.PC.F; BLD.CD19.PPC; BLD.CD56.PC; BLD.CD15.PC; THYM.FET; BRN.HIPP.MID; BRN.SUB.NIG; MUS.SKLT.F; HRT.ATR.R; HRT.VENT.L; HRT.VNT.R; GI.CLN.SM.MUS; GI.STMC.FET; GI.S.INT; GI.STMC.MUC; PLCNT.FET; PANC)<br><br>**9_TxReg** (FAT.ADIP.NUC; MUS.SKLT.M; GI.DUO.SM.MUS; GI.RECT.SM.MUS; GI.STMC.MUS; GI.S.INT.FET; GI.L.INT.FET; LIV.ADLT; GI.CLN.SIG; GI.CLN.MUC; GI.RECT.MUC.29; GI.RECT.MUC.31; GI.DUO.MUC; LNG; SPLN)	M; BONE.OSTEO)<br><br>**16_EnhW2** (MUS.HSMMT)<br><br>**9_TxReg** (BLD.DND41.CNCR)<br><br>**12_TxEnhW** (BLD.GM12878)<br><br>**11_TxEnh3** (BLD.K562.CNCR)<br><br>**13_EnhA1** (LIV.HEPG2.CNCR)<br><br>**10_TxEnh5** (BLD.CD14.MONO; LNG.NHLF)<br><br>**15_EnhAF** (SKIN.NHEK)	ESDR.H1.BMP4.TROP; ESDR.H1.MSC; BLD.PER.MONUC.PC; BLD.CD3.PPC; BLD.CD4.CD25I.CD127.TMEMPC; BLD.CD4.CD25.CD127M.TREGPC; BLD.CD4.CD25M.TPC; BLD.CD4.CD25M.CD45RA.NPC; BLD.CD4.CD25M.IL17M.PL.TPC; BLD.CD4.CD25M.IL17P.PL.TPC; BLD.CD4.CD25M.CD45RO.MPC; BLD.CD4.MPC; BLD.CD8.MPC; BLD.CD4.NPC; BLD.CD8.NPC; BLD.CD14.PC; BLD.CD19.CPC; BLD.CD34.PC; BLD.MOB.CD34.PC.M; BLD.MOB.CD34.PC.F; BLD.CD34.CC; BLD.CD19.PPC; BLD.CD56.PC; BLD.CD15.PC; STRM.CHON.MRW.DR.MSC; FAT.ADIP.DR.MSC; SKIN.PEN.FRSK.FIB.01; SKIN.PEN.FRSK.FIB.02; SKIN.PEN.FRSK.KER.02; SKIN.PEN.FRSK.KER.03; BRST.HMEC.35; BRST.MYO; THYM; THYM.FET; BRN.HIPP.MID; BRN.SUB.NIG; BRN.ANT.CAUD; BRN.CING.GYR; BRN.INF.TMP; BRN.ANG.GYR; BRN.DL.PRFRNTL.CRTX; FAT.ADIP.NUC; MUS.PSOAS; MUS.SKLT.F; MUS.SKLT.M; MUS.TRNK.FET; HRT.FET; HRT.ATR.R; HRT.VENT.L; HRT.VNT.R; VAS.AOR; GI.DUO.SM.MUS; GI.CLN.SM.MUS; GI.RECT.SM.MUS; GI.STMC.MUS; GI.STMC.FET; GI.S.INT.FET; GI.L.INT.FET; GI.S.INT; GI.CLN.SIG; GI.CLN.MUC; GI.RECT.MUC.29; GI.RECT.MUC.31; GI.STMC.MUC; GI.DUO.MUC; GI.ESO; GI.STMC.GAST; PLCNT.AMN; KID.FET; LNG.FET; OVRY;	US; GI.CLN.SM.MUS; GI.RECT.SM.MUS; GI.STMC.MUS; GI.CLN.SIG; GI.CLN.MUC; GI.RECT.MUC.29; GI.DUO.MUC; SPLN.	PC; BLD.CD4.CD25M.IL17P.PL.TPC; BLD.CD4.CD25M.CD45RO.MPC; BLD.CD4.MPC; BLD.CD4.NPC; BLD.CD14.PC; BLD.MOB.CD34.PC.F; BLD.CD56.PC; THYM; THYM.FET; BRN.ANT.CAUD; BRN.CING.GYR; BRN.INF.TMP; FAT.ADIP.NUC; MUS.SKLT.F; HRT.ATR.R; HRT.VENT.L; HRT.VNT.R; GI.DUO.SM.MUS; GI.CLN.SM.MUS; GI.RECT.SM.MUS; GI.STMC.MUS; GI.STMC.FET; GI.S.INT.FET; GI.L.INT.FET; GI.S.INT; GI.CLN.SIG; GI.CLN.MUC; GI.RECT.MUC.29; GI.RECT.MUC.31; GI.ESO; GI.STMC.GAST; PLCNT.FET; LIV.ADLT; PANC; LNG; SPLN	BRN.CING.GYR; FAT.ADIP.NUC; MUS.SKLT.M; GI.CLN.MUC; GI.RECT.MUC.29; GI.RECT.MUC.31; GI.DUO.MUC.	MUS.HSMM; MUS.HSMMT; BLD.K562.CNCR; BLD.CD14.MONO; BRN.NHA; SKIN.NHDFAD; SKIN.NHEK; LNG.NHLF.<br><br>**H3K27ac** BLD.DND41.CNCR; BLD.GM12878; LIV.HEPG2.CNCR; MUS.HSMMT; BLD.CD14.MONO; LNG.NHLF; BONE.OSTEO	**H3K9ac** BLD.DND41.CNCR; LIV.HEPG2.CNCR; BLD.CD14.MONO.	LIV.HEPG2.CNCR; BLD.K562.CNCR

			ADRL.GLND.FET; PLCNT.FET; LIV.ADLT; PANC; LNG; SPLN						
rs17622378	**12_TxEnhW**<br>(BLD.PER.MONUC.PC; BLD.CD4.CD25I.CD127.TMEMPC; BLD.CD3.CPC; BLD.CD4.CD25.CD127M.TREGPC)<br><br>**10_TxEnh5**<br>(BLD.CD3.PPC; BLD.CD4.CD25M.TPC; BLD.CD4.CD25M.CD45RA.NPC; BLD.CD4.CD25M.IL17M.PL.TPC; BLD.CD4.CD25M.IL17P.PL.TPC; BLD.CD4.CD25M.CD45RO.MPC; BLD.CD4.MPC; BLD.CD8.MPC; BLD.CD4.NPC; BLD.CD8.NPC; BLD.MOB.CD34.PC.F; BLD.CD56.PC; THYM.FET)<br><br>**17_EnhW2**<br><br>(BLD.CD14.PC; MUS.SKLT.M; GI.RECT.SM.MUS; GI.RECT.MUC.31; GI.STMC.MUC)<br><br>**12_TxEnhW2**<br><br>(BLD.CD19.CPC; BLD.CD34.PC; BLD.MOB.CD34.PC.M; BLD.CD34.CC; BLD.CD19.PPC; BLD.CD15.PC; THYM; FAT.ADIP.NUC; GI.S.INT; GI.CLN.SIG; GI.DUO.MUC; PLCNT.FET; LIV.ADLT; LNG; SPLN)	**12_TxEnhW2**<br><br>(BLD.DND41.CNCR; BLD.GM12878; BLD.CD14.MONO)	IPSC.DF.6.9; IPSC.DF.19.11; BLD.PER.MONUC.PC; BLD.CD3.PPC; BLD.CD4.CD25I.CD127.TMEMPC; BLD.CD3.CPC; BLD.CD4.CD25.CD127M.TREGPC; BLD.CD4.CD25M.TPC; BLD.CD4.CD25M.CD45RA.NPC; BLD.CD4.CD25M.IL17M.PL.TPC; BLD.CD4.CD25M.IL17P.PL.TPC; BLD.CD4.CD25M.CD45RO.MPC; BLD.CD4.MPC; BLD.CD8.MPC; BLD.CD4.NPC; BLD.CD8.NPC; BLD.CD14.PC; BLD.CD19.CPC; BLD.CD34.PC; BLD.MOB.CD34.PC.M; BLD.MOB.CD34.PC.F; BLD.CD34.CC; BLD.CD19.PPC; BLD.CD56.PC; BLD.CD15.PC; SKIN.PEN.FRSK.FIB.01; SKIN.PEN.FRSK.FIB.02; SKIN.PEN.FRSK.KER.03; BRST.MYO; THYM; THYM.FET; BRN.HIPP.MID; BRN.ANT.CAUD; BRN.CING.GYR; BRN.ANG.GYR; FAT.ADIP.NUC; MUS.PSOAS; MUS.SKLT.F; MUS.SKLT.M; MUS.LEG.FET; HRT.ATR.R; HRT.VENT.L; HRT.VNT.R; VAS.AOR; GI.DUO.SM.MUS; GI.CLN.SM.MUS; GI.RECT.SM.MUS; GI.STMC.MUS; GI.STMC.FET; GI.S.INT.FET; GI.L.INT.FET; GI.S.INT; GI.CLN.SIG; GI.RECT.MUC.29; GI.RECT.MUC.31; GI.STMC.MUC; GI.DUO.MUC; GI.ESO; GI.STMC.GAST; PLCNT.AMN; PLCNT.FET; LIV.ADLT; PANC; LNG; SPLN	ESDR.H1.BMP4.TROP; BLD.CD8.MPC; BLD.CD4.NPC; GI.STMC.MUS; LNG	ESC.H9; ESC.H1; BLD.PER.MONUC.PC; BLD.CD3.PPC; BLD.CD4.CD25I.CD127.TMEMPC; BLD.CD4.CD25.CD127M.TREGPC; BLD.CD4.CD25M.TPC; BLD.CD4.CD25M.IL17M.PL.TPC; BLD.CD4.CD25M.IL17P.PL.TPC; BLD.CD4.CD25M.CD45RO.MPC; BLD.CD8.MPC; BLD.CD8.NPC; BLD.MOB.CD34.PC.F; BLD.CD56.PC; SKIN.PEN.FRSK.FIB.02; THYM; THYM.FET; BRN.HIPP.MID; BRN.ANT.CAUD; FAT.ADIP.NUC; MUS.PSOAS; HRT.VENT.L; HRT.VNT.R; GI.RECT.SM.MUS; GI.S.INT; GI.CLN.SIG; GI.RECT.MUC.29; PLCNT.FET; LIV.ADLT; LNG; SPLN		**H3K4me1**<br>BLD.DND41.CNCR; BLD.GM12878; BLD.CD14.MONO<br><br>**H3K27ac**<br>BLD.DND41.CNCR; BLD.GM12878; BLD.CD14.MONO.	**H3K4me3**<br>None<br><br>**H3K9ac**<br>ROADMAP<br>BLD.PER.MONUC.PC; BRN.ANG.GYR; FAT.ADIP.NUC; MUS.SKLT.M; HRT.FET; GI.RECT.SM.MUS; GI.STMC.MUS; GI.RECT.MUC.29.	
rs12521868	**18_EnhAc**	**10_TxEnh5**<br>(BLD.DND41.CNCR; BLD.CD14.MONO;	ESC.HUES6; PSC.DF.6.9; ESDR.H1.BMP4.MESO; ESDR.H1.MSC; BLD.PER.MONUC.PC; BLD.CD3.PPC;	ROADMAP<br>BLD.CD4.CD25M.IL17M.PL.TPC; BLD.CD4.CD25	(ESC.H1; IPSC.20B; ESDR.H1.BMP4.TROP; BLD.PER.MONUC.PC; BLD.CD3.PPC; BLD.CD4.CD25I.CD127.TME	ESC.HUES6; BRN.INF.TMP; BRN.ANG.GYR; FAT.ADIP.NUC; MUS.SKLT.M;	**H3K4me1**<br>BLD.DND41.CNCR; BLD.CD14.	**H3K4me3**<br>None<br><br>**H3K9ac**	

	(ESDR.H1.BMP4.TROP; MUS.SKLT.F; HRT.ATR.R; HRT.VNT.R; GI.RECT.MUC.29; PLCNT.AMN; LIV.ADLT)<br><br>**12_TxEnhW**<br><br>(BLD.PER.MONUC.PC; BLD.CD19.CPC; BLD.CD19.PPC; THYM; GI.CLN.SIG; GI.DUO.MUC; PLCNT.FET; SPLN)<br><br>**10_TxEnh5**<br><br>(BLD.CD3.PPC; BLD.CD4.CD25.CD127M.TREGPC; BLD.CD4.CD25M.TPC; BLD.CD4.CD25M.CD45RA.NPC; BLD.CD4.CD25M.IL17M.PL.TPC; BLD.CD4.CD25M.CD45RO.MPC; BLD.CD4.MPC; BLD.CD8.MPC; BLD.CD4.NPC; BLD.CD8.NPC; BLD.MOB.CD34.PC.F; BLD.CD56.PC; BLD.CD15.PC; THYM.FET; FAT.ADIP.NUC; HRT.VENT.L; GI.DUO.SM.MUS)<br><br>**11_TxEnh5**<br>(BLD.CD4.CD25M.IL17P.PL.TPC; GI.CLN.SM.MUS)<br><br>**15_EnhAF**<br>(BLD.CD14.PC)<br><br>**17_EnhW2**<br>(SKIN.PEN.FRSK.FIB.02; GI.STMC.MUS; GI.S.INT; LNG)	BRN.NHA; SKIN.NHDFAD; SKIN.NHEK; LNG.NHLF; BONE.OSTEO)	BLD.CD4.CD25I.CD127.TMEMPC; BLD.CD4.CD25.CD127M.TREGPC; BLD.CD4.CD25M.TPC; BLD.CD4.CD25M.CD45RA.NPC; BLD.CD4.CD25M.IL17M.PL.TPC; BLD.CD4.CD25M.IL17P.PL.TPC; BLD.CD4.CD25M.CD45RO.MPC; BLD.CD4.MPC; BLD.CD8.MPC; BLD.CD4.NPC; BLD.CD8.NPC; BLD.CD14.PC; BLD.CD56.PC; BLD.CD15.PC; FAT.ADIP.DR.MSC; SKIN.PEN.FRSK.FIB.01; SKIN.PEN.FRSK.FIB.02; SKIN.PEN.FRSK.KER.02; SKIN.PEN.FRSK.KER.03; BRST.HMEC.35; THYM; THYM.FET; BRN.SUB.NIG; BRN.CING.GYR; BRN.ANG.GYR; BRN.DL.PRFRNTL.CRTX; FAT.ADIP.NUC; MUS.PSOAS; MUS.SKLT.F; MUS.SKLT.M; MUS.LEG.FET; HRT.ATR.R; HRT.VENT.L; HRT.VNT.R; GI.DUO.SM.MUS; GI.CLN.SM.MUS; GI.RECT.SM.MUS; GI.STMC.MUS; GI.STMC.FET; GI.S.INT.FET; GI.S.INT; GI.CLN.SIG; GI.CLN.MUC; GI.RECT.MUC.29; GI.RECT.MUC.31; GI.STMC.MUC; GI.DUO.MUC; GI.ESO; GI.STMC.GAST; PLCNT.FET; LIV.ADLT; PANC; LNG; SPLN	M.IL17P.PL.TPC; GI.CLN.SM.MUS; GI.RECT.SM.MUS;	MPC; BLD.CD4.CD25.CD127M.TREGPC; BLD.CD4.CD25M.TPC; BLD.CD4.CD25M.CD45RA.NPC; BLD.CD4.CD25M.IL17M.PL.TPC; BLD.CD4.CD25M.IL17P.PL.TPC; BLD.CD4.CD25M.CD45RO.MPC; BLD.CD4.MPC; BLD.CD8.MPC; BLD.CD4.NPC; BLD.MOB.CD34.PC.F; BLD.CD56.PC; SKIN.PEN.FRSK.FIB.02; THYM; THYM.FET; BRN.CING.GYR; FAT.ADIP.NUC; HRT.ATR.R; HRT.VENT.L; HRT.VNT.R; GI.DUO.SM.MUS; GI.CLN.SM.MUS; GI.RECT.SM.MUS; GI.STMC.MUS; GI.STMC.FET; GI.S.INT; GI.CLN.SIG; GI.CLN.MUC; GI.RECT.MUC.31; GI.ESO; PLCNT.FET; LIV.ADLT; LNG; SPLN)	GI.CLN.MUC; GI.RECT.MUC.29; GI.RECT.MUC.31; GI.STMC.MUC; GI.DUO.MUC.	MONO; LNG.NHLF<br><br>**H3K27ac**<br>BLD.DND41.CNCR; BLD.CD14.MONO; SKIN.NHEK.	CRVX.HELAS3.CNCR	
rs12515180	**18_EnhAc**<br><br>(LNG.IMR90; ESDR.H1.BMP4.TROP; PLCNT.AMN)<br><br>**10_TxEnh5**<br>(BLD.CD3.PPC; BLD.CD56.PC; THYM.FET; PLCNT.FET)<br><br>**12_TxEnhW**<br>(BLD.CD4.CD25.CD127M.TREGPC)	**12_TxEnhW**<br>(BLD.DND41.CNCR)	LNG.IMR90; ESC.HUES6; ESC.HUES48; ESC.HUES64; ESC.4STAR; ESDR.H1.MSC; BLD.PER.MONUC.PC; BLD.CD3.PPC; BLD.CD4.CD25.CD127M.TREGPC; BLD.CD4.CD25M.TPC; BLD.CD4.CD25M.CD45RA.NPC; BLD.CD4.CD25M.IL17M.PL.TPC; BLD.CD4.CD25M.IL17P.PL.TPC; BLD.CD4.CD25M.CD45RO.MPC; BLD.CD4.MPC; BLD.CD8.MPC;	IPSC.20B; BLD.CD4.CD25M.IL17P.PL.TPC	LNG.IMR90; ESC.H1; BLD.CD3.PPC; BLD.CD4.CD25I.CD127.TMEMPC; BLD.CD4.CD25.CD127M.TREGPC; BLD.CD4.CD25M.CD45RA.NPC; BLD.CD4.CD25M.IL17M.PL.TPC; BLD.CD4.CD25M.IL17P.PL.TPC;	ESC.H9; ESC.HUES6; FAT.ADIP.NUC; GI.RECT.MUC.29; GI.STMC.MUC; GI.DUO.MUC; LNG.FET;	**H3K4me1**<br>BLD.DND41.CNCR; LNG.NHLF<br><br>**H3K27ac**<br>BLD.DND41.CNCR; BLD.CD14.MONO; LNG.NHLF;	**H3K4me3**<br>None<br><br>**H3K9ac**<br>LNG.A549.ETOH002.CNCR; BLD.DND41.CNCR;	

	**11_TxEnh3**<br>(BLD.CD4.CD25M.IL17M.PL.TPC; BLD.CD4.CD25M.IL17P.PL.TPC; BLD.CD14.PC; BLD.CD19.PPC; SKIN.PEN.FRSK.FIB.02; SPLN)<br><br>**17_EnhW2**<br>(SKIN.PEN.FRSK.FIB.01)		BLD.CD4.NPC; BLD.CD8.NPC; BLD.MOB.CD34.PC.F; BLD.CD56.PC; BLD.CD15.PC; FAT.MSC.DR.ADIP; SKIN.PEN.FRSK.FIB.02; SKIN.PEN.FRSK.KER.02; SKIN.PEN.FRSK.KER.03; THYM; THYM.FET; FAT.ADIP.NUC; HRT.ATR.R; HRT.VENT.L; HRT.VNT.R; GI.DUO.SM.MUS; GI.STMC.MUS; GI.STMC.FET; GI.S.INT.FET; GI.S.INT; GI.RECT.MUC.29; GI.STMC.MUC; GI.DUO.MUC; GI.ESO; GI.STMC.GAST; LNG.FET; OVRY; PLCNT.FET; LIV.ADLT; PANC; LNG; SPLN		BLD.CD4.CD25M.CD45RO.MPC; BLD.CD8.MPC; BLD.CD4.NPC; BLD.CD19.PPC; BLD.CD56.PC; SKIN.PEN.FRSK.FIB.01; SKIN.PEN.FRSK.FIB.02; THYM; THYM.FET; FAT.ADIP.NUC; HRT.VNT.R; GI.DUO.SM.MUS; GI.CLN.SM.MUS; GI.RECT.SM.MUS; GI.S.INT; GI.CLN.SIG; GI.CLN.MUC; GI.RECT.MUC.31; OVRY; PLCNT.FET; LIV.ADLT; LNG; SPLN		BONE.OSTEO	BLD.GM12878; CRVX.HELAS3.CNCR; LIV.HEPG2.CNCR; BRST.HMEC; MUS.HSMM; MUS.HSMMT; VAS.HUVEC; BLD.K562.CNCR; BLD.CD14.MONO; BRN.NHA; SKIN.NHDFAD; SKIN.NHEK; LNG.NHLF; BONE.OSTEO	
rs146604341	**18_EnhAc**<br>(LNG.IMR90; ESDR.H1.BMP4.TROP; PLCNT.AMN)<br><br>**17_EnhW2**<br>(ESC.WA7; ESC.H9; ESC.I3; ESC.HUES6; ESC.HUES48; ESC.H1; IPSC.15b; ESDR.CD184.ENDO; ESDR.H1.BMP4.MESO; FAT.ADIP.NUC; GI.DUO.SM.MUS; KID.FET; PLCNT.FET)<br><br>**14_EnhA2**<br>(ESC.HUES64; IPSC.18)<br><br>**16_EnhW1**<br>(IPSC.20B)	None	LNG.IMR90; ESC.I3; ESC.HUES6; ESC.HUES48; ESC.HUES64; ESC.4STAR; IPSC.20B; IPSC.18; IPSC.15b; ESDR.H1.BMP4.MESO; ESDR.H1.BMP4.TROP; BLD.CD3.PPC; BLD.CD4.CD25.CD127M.TREGPC; BLD.CD4.CD25M.TPC; BLD.CD4.CD25M.CD45RA.NPC; BLD.CD4.CD25M.IL17M.PL.TPC; BLD.CD4.CD25M.IL17P.PL.TPC; BLD.CD4.CD25M.CD45RO.MPC; BLD.CD4.MPC; BLD.CD8.MPC; BLD.CD4.NPC; BLD.CD8.NPC; BLD.MOB.CD34.PC.F; BLD.CD56.PC; BLD.CD15.PC;	ESC.HUES64; IPSC.20B;	LNG.IMR90; ESC.HUES48; ESC.H1; IPSC.20B; BLD.CD3.PPC; BLD.CD4.CD25I.CD127.TMEMPC; BLD.CD4.CD25.CD127M.TREGPC; BLD.CD4.CD25M.CD45RA.NPC; BLD.CD4.CD25M.IL17M.PL.TPC; BLD.CD4.CD25M.IL17P.PL.TPC; BLD.CD4.CD25M.CD45RO.MPC; BLD.CD8.MPC; BLD.CD4.NPC;	LNG.IMR90; ESC.H9; FAT.ADIP.NUC; GI.STMC.MUC; GI.DUO.MUC; LNG.FET; LIV.ADLT	**H3K4me1**<br>BLD.DND41.CNCR; BLD.K562.CNCR; SKIN.NHEK; LNG.NHLF; BONE.OSTEO<br><br>**H3K27ac**<br>BLD.DND41.CNCR; BLD.CD14.	**H3K4me3**<br>None<br><br>**H3K9ac**<br>None	ROADMAP<br>SKIN.PEN.FRSK.KER.02; OVRY; PLCNT.FET<br><br>ENCODE

	**11_TxEnh3**<br>(BLD.CD4.CD25I.CD127.TMEMPC; BLD.CD4.CD25M.IL17P.PL.TPC)<br><br>**12_TxEnhW**<br>(BLD.CD4.CD25M.CD45RA.NPC)<br><br>**19_DNase**<br>(SKIN.PEN.FRSK.KER.03; BRST.HMEC.35)<br><br>**15_EnhAF**<br>(LIV.ADLT)		STRM.MRW.MSC; FAT.MSC.DR.ADIP; SKIN.PEN.FRSK.FIB.01; SKIN.PEN.FRSK.FIB.02; SKIN.PEN.FRSK.KER.02; SKIN.PEN.FRSK.KER.03; BRST.HMEC.35; THYM.FET; FAT.ADIP.NUC; GI.DUO.SM.MUS; GI.STMC.FET; GI.S.INT.FET; GI.S.INT; GI.CLN.MUC; GI.RECT.MUC.29; GI.STMC.MUC; GI.DUO.MUC; LNG.FET; PLCNT.FET; LIV.ADLT		BLD.CD19.PPC; STRM.MRW.MSC; SKIN.PEN.FRSK.FIB.02; THYM.FET; FAT.ADIP.NUC; GI.DUO.SM.MUS; GI.S.INT; GI.CLN.SIG; GI.CLN.MUC;.STMC.GAST; PLCNT.AMN; KID.FET; LNG.FET; OVRY; PANC.ISLT; ADRL.GLND.FET; PLCNT.FET; LIV.ADLT; SPLN		MONO; LNG.NHLF		
rs11951091	**18_EnhAc**<br><br>(LNG.IMR90; ESDR.H1.BMP4.TROP; PLCNT.AMN)<br><br>**17_EnhW2**<br>(ESC.WA7; ESC.H9; ESC.I3; ESC.HUES6; ESC.HUES48; ESC.H1; IPSC.15b; ESDR.CD184.ENDO; ESDR.H1.BMP4.MESO; FAT.ADIP.NUC; GI.DUO.SM.MUS; KID.FET; PLCNT.FET)<br><br>**14_EnhA2**<br>(ESC.HUES64; IPSC.18)<br><br>**16_EnhW1**<br>(IPSC.20B)<br><br>**11_TxEnh3**<br>(BLD.CD4.CD25I.CD127.TMEMPC; BLD.CD4.CD25M.IL17P.PL.TPC)<br><br>**12_TxEnhW**<br>(BLD.CD4.CD25M.CD45RA.NPC)<br><br>**19_DNase**<br>(SKIN.PEN.FRSK.KER.03; BRST.HMEC.35)<br><br>**15_EnhAF**<br>(LIV.ADLT)	None	LNG.IMR90; ESC.WA7; ESC.H9; ESC.I3; ESC.HUES6; ESC.HUES48; ESC.HUES64; ESC.4STAR; IPSC.20B; IPSC.18; IPSC.15b; ESDR.H1.BMP4.MESO; ESDR.H1.BMP4.TROP; BLD.CD3.PPC; BLD.CD4.CD25.CD127M.TREGPC; BLD.CD4.CD25M.TPC; BLD.CD4.CD25M.CD45RA.NPC; BLD.CD4.CD25M.IL17M.PL.TPC; BLD.CD4.CD25M.IL17P.PL.TPC; BLD.CD4.CD25M.CD45RO.MPC; BLD.CD4.MPC; BLD.CD8.MPC; BLD.CD4.NPC; BLD.CD8.NPC; BLD.MOB.CD34.PC.F; BLD.CD56.PC; BLD.CD15.PC; STRM.MRW.MSC; STRM.CHON.MRW.DR.MSC; FAT.ADIP.DR.MSC; FAT.MSC.DR.ADIP; SKIN.PEN.FRSK.FIB.01; SKIN.PEN.FRSK.FIB.02; SKIN.PEN.FRSK.KER.02; SKIN.PEN.FRSK.KER.03; BRST.HMEC.35; THYM.FET; FAT.ADIP.NUC; MUS.SKLT.F; MUS.SKLT.M; GI.DUO.SM.MUS; GI.STMC.FET; GI.S.INT.FET; GI.S.INT; GI.CLN.MUC; GI.RECT.MUC.29; GI.STMC.MUC; GI.DUO.MUC; LNG.FET; OVRY; PLCNT.FET; LIV.ADLT	ESC.HUES48; ESC.HUES64; IPSC.20B.	LNG.IMR90; ESC.HUES48; ESC.H1; IPSC.20B; ESDR.H1.MSC; BLD.CD3.PPC; BLD.CD4.CD25I.CD127.TMEMPC; BLD.CD4.CD25.CD127M.TREGPC; BLD.CD4.CD25M.CD45RA.NPC; BLD.CD4.CD25M.IL17M.PL.TPC; BLD.CD4.CD25M.IL17P.PL.TPC; BLD.CD4.CD25M.CD45RO.MPC; BLD.CD8.MPC; BLD.CD4.NPC; BLD.CD19.PPC; BLD.CD56.PC; STRM.MRW.MSC; SKIN.PEN.FRSK.FIB.02; THYM; THYM.FET; FAT.ADIP.NUC; GI.DUO.SM.MUS; GI.S.INT; GI.CLN.SIG; GI.CLN.MUC; GI.RECT.MUC.31; GI.ESO; PLCNT.FET; LIV.ADLT; SPLN	LNG.IMR90; ESC.H9; ESC.HUES64; ESC.H1; FAT.ADIP.NUC; GI.STMC.MUC; GI.DUO.MUC; LNG.FET; LIV.ADLT	**H3K4me1**<br>BLD.DND41.CNCR; BRST.HMEC; MUS.HSMM; MUS.HSMMT; VAS.HUVEC; BLD.K562.CNCR; BRN.NHA; SKIN.NHEK; LNG.NHLF; BONE.OSTEO.<br><br>**H3K27ac**<br>BLD.DND41.CNCR; BLD.CD14.MONO; LNG.NHLF	**H3K4me3**<br>None<br><br>**H3K9ac**<br>LNG.A549.ETOH002.CNCR; BLD.DND41.CNCR; BLD.GM12878; CRVX.HELAS3.CNCR; LIV.HEPG2.CNCR; BRST.HMEC; MUS.HSMM; MUS.HSMMT; VAS.HUVEC; BLD.K562.CNCR; BLD.CD14.MONO;	ROADMAP ESC.H1; IPSC.DF.6.9; IPSC.DF.19.11; ESDR.H1.MSC; SKIN.PEN.FRSK.KER.02; PLCNT.FET

								BRN.NHA; SKIN.NHDFAD; SKIN.NHEK; LNG.NHLF; BONE.OSTEO	
rs6866614	**14_EnhW2**<br>(LNG.IMR90; IPSC.18)<br><br>**17_EnhW2**<br>(ESC.WA7; ESC.I3; ESC.HUES6; ESC.HUES48; ESC.HUES64; ESC.H1; ESC.4STAR; IPSC.20B; PSC.DF.6.9; IPSC.DF.19.11; ESDR.H1.NEUR.PROG; ESDR.H9.NEUR; ESDR.CD56.ECTO; ESDR.CD184.ENDO; ESDR.H1.BMP4.MESO; ESDR.H1.MSC; STRM.MRW.MSC; FAT.ADIP.DR.MSC; FAT.MSC.DR.ADIP; MUS.SAT; SKIN.PEN.FRSK.FIB.01; SKIN.PEN.FRSK.FIB.02; SKIN.PEN.FRSK.MEL.01; SKIN.PEN.FRSK.KER.02; BRST.HMEC.35; BRST.MYO; LNG)<br><br>**15_EnhAF**<br>(ESC.H9; ESDR.H1.BMP4.TROP)<br><br>**16_EnhW1**<br>(IPSC.15b; STRM.CHON.MRW.DR.MSC)<br><br>**10_TxEnh5**<br>(BLD.CD3.PPC; BLD.CD4.CD25M.TPC; PLCNT.FET)<br><br>**12_TxEnhW**<br>(BLD.CD4.CD25M.CD45RA.NPC; BLD.CD4.NPC)<br><br>**11_TxEnh3**<br>(BLD.CD4.CD25M.IL17P.PL.TPC; BLD.CD14.PC; BLD.CD56.PC;	**17_EnhW2**<br><br>(LNG.A549.ETOH002.CNCR; CRVX.HELAS3.CNCR; MUS.HSMM; MUS.HSMMT; BRN.NHA; SKIN.NHDFAD; SKIN.NHEK; LNG.NHLF)<br><br>**10_TxEnhW2**<br><br>(BLD.DND41.CNCR)<br><br>**11_TxEnh3**<br>(BLD.K562.CNCR)<br><br>**15_EnhAF**<br>(BONE.OSTEO)	LNG.IMR90; ESC.WA7; ESC.H9; ESC.I3; ESC.HUES6; ESC.HUES48; ESC.HUES64; ESC.H1; ESC.4STAR; IPSC.20B; IPSC.18; IPSC.15b; PSC.DF.6.9; ESDR.H1.NEUR.PROG; ESDR.H1.BMP4.MESO; ESDR.H1.BMP4.TROP; ESDR.H1.MSC; BLD.CD3.PPC; BLD.CD4.CD25I.CD127.TMEMPC; BLD.CD4.CD25.CD127M.TREGPC; BLD.CD4.CD25M.TPC; BLD.CD4.CD25M.CD45RA.NPC; BLD.CD4.CD25M.IL17M.PL.TPC; BLD.CD4.CD25M.IL17P.PL.TPC; BLD.CD4.CD25M.CD45RO.MPC; BLD.CD4.MPC; BLD.CD8.MPC; BLD.CD4.NPC; BLD.CD8.NPC; BLD.MOB.CD34.PC.F; BLD.CD19.PPC; BLD.CD56.PC; BLD.CD15.PC; STRM.MRW.MSC; STRM.CHON.MRW.DR.MSC; FAT.ADIP.DR.MSC; FAT.MSC.DR.ADIP; SKIN.PEN.FRSK.FIB.01; SKIN.PEN.FRSK.FIB.02; SKIN.PEN.FRSK.KER.02; SKIN.PEN.FRSK.KER.03; BRST.HMEC.35; THYM.FET; MUS.PSOAS; MUS.SKLT.F; MUS.SKLT.M; GI.DUO.SM.MUS; GI.STMC.FET; GI.S.INT.FET; GI.S.INT; GI.CLN.MUC; GI.RECT.MUC.29; GI.STMC.MUC; GI.DUO.MUC; GI.ESO; PLCNT.AMN; LNG.FET; PLCNT.FET; LIV.ADLT; LNG; SPLN	HUES48; IPSC.20B; BLD.CD4.CD25M.IL17P.PL.TPC; BLD.CD4.NPC	LNG.IMR90; ESC.HUES48; ESC.H1; IPSC.20B; IPSC.DF.6.9; BLD.CD3.PPC; BLD.CD4.CD25I.CD127.TMEMPC; BLD.CD4.CD25.CD127M.TREGPC; BLD.CD4.CD25M.CD45RA.NPC; BLD.CD4.CD25M.IL17M.PL.TPC; BLD.CD4.CD25M.IL17P.PL.TPC; BLD.CD4.CD25M.CD45RO.MPC; BLD.CD4.MPC; BLD.CD8.MPC; BLD.CD4.NPC; BLD.CD19.PPC; BLD.CD56.PC; STRM.MRW.MSC; SKIN.PEN.FRSK.FIB.02; THYM; THYM.FET; FAT.ADIP.NUC; HRT.VNT.R; GI.DUO.SM.MUS; GI.S.INT; GI.CLN.SIG; GI.CLN.MUC; GI.RECT.MUC.31; GI.ESO; PLCNT.FET; LIV.ADLT; LNG; SPLN	LNG.IMR90; ESC.H9; ESC.HUES64; ESC.H1; ESDR.H1.BMP4.TROP; FAT.ADIP.NUC; GI.STMC.MUC; LNG.FET.	**H3K4me1**<br>BLD.DND41.CNCR; BRST.HMEC; BLD.K562.CNCR; BRN.NHA; SKIN.NHDFAD; SKIN.NHEK; LNG.NHLF; BONE.OSTEO<br><br>**H3K27ac**<br>BLD.DND41.CNCR; BLD.CD14.MONO; LNG.NHLF	**H3K4me3**<br>None<br><br>**H3K9ac**<br>None	ROADMAP PLCNT.FET

	BLD.CD15.PC; FAT.ADIP.NUC; MUS.SKLT.M)<br><br>**10_TxEnh5**<br>(BLD.CD4.CD25M.CD45RO.MPC)<br><br>**18_EnhAc**<br>(PLCNT.AMN)								
rs4705950	**13_EnhA1**<br>(LNG.IMR90; ESDR.H1.MSC; BLD.PER.MONUC.PC; BLD.CD3.CPC; BLD.CD34.PC; STRM.MRW.MSC; STRM.CHON.MRW.DR.MSC; FAT.ADIP.DR.MSC; FAT.MSC.DR.ADIP; MUS.SAT; SKIN.PEN.FRSK.FIB.01; SKIN.PEN.FRSK.FIB.02; SKIN.PEN.FRSK.KER.02; FAT.ADIP.NUC; MUS.SKLT.F; GI.L.INT.FET; GI.S.INT; GI.CLN.SIG; GI.CLN.MUC; GI.RECT.MUC.29; GI.RECT.MUC.31; GI.STMC.MUC; GI.ESO; PLCNT.FET; LIV.ADLT; LNG; SPLN)<br><br>**17EnhW2**<br>ESC.WA7; ESC.H9; ESC.I3;<br><br>**19_DNase**<br>(ESC.HUES6; IPSC.18)<br><br>**17_EnhW2**<br>(ESC.H1; ESC.4STAR; IPSC.15b; PSC.DF.6.9; ESDR.H9.NEUR.PROG; BRN.CRTX.DR.NRSPHR)<br><br>**15_EnhAF**<br>(IPSC.DF.19.11; ESDR.H1.BMP4.TROP; SKIN.PEN.FRSK.MEL.01; SKIN.PEN.FRSK.MEL.03; BRST.HMEC.35; BRN.SUB.NIG; BRN.ANT.CAUD; BRN.CING.GYR; BRN.INF.TMP; BRN.ANG.GYR; BRN.FET.M; MUS.LEG.FET; HRT.FET; HRT.ATR.R; HRT.VENT.L; HRT.VNT.R; VAS.AOR; GI.STMC.GAST; KID.FET; OVRY; PANC.ISLT; ADRL.GLND.FET; PANC)<br><br>**14_EnhA2**	**15_EnhAF**<br><br>(LNG.A549.ETOH002.CNCR; CRVX.HELAS3.CNCR; LIV.HEPG2.CNCR; BRST.HMEC; VAS.HUVEC; BLD.K562.CNCR; BRN.NHA)<br><br>**3_PromD1**<br>(BLD.DND41.CNCR)<br><br>**9_TxReg**<br>(BLD.GM12878; BLD.CD14.MONO)<br><br>**13_EnhA1**<br>(MUS.HSMM; MUS.HSMMT; SKIN.NHDFAD; SKIN.NHEK; LNG.NHLF;	LNG.IMR90; ESC.WA7; ESC.H9; ESC.I3; ESC.HUES6; ESC.HUES48; ESC.H1; ESC.4STAR; IPSC.20B; IPSC.18; IPSC.15b; IPSC.DF.19.11; ESDR.H9.NEUR.PROG; ESDR.CD56.MESO; ESDR.H1.BMP4.TROP; ESDR.H1.MSC; BLD.PER.MONUC.PC; BLD.CD3.PPC; BLD.CD4.CD25I.CD127.TMEMPC; BLD.CD3.CPC; BLD.CD4.CD25.CD127M.TREGPC; BLD.CD4.CD25M.TPC; BLD.CD4.CD25M.CD45RA.NPC; BLD.CD4.CD25M.IL17M.PL.TPC; BLD.CD4.CD25M.IL17P.PL.TPC; BLD.CD4.CD25M.CD45RO.MPC; BLD.CD4.MPC; BLD.CD8.MPC; BLD.CD4.NPC; BLD.CD8.NPC; BLD.CD14.PC; BLD.CD19.CPC; BLD.CD34.PC; BLD.MOB.CD34.PC.M; BLD.MOB.CD34.PC.F; BLD.CD34.CC; BLD.CD19.PPC; BLD.CD56.PC; BLD.CD15.PC; STRM.MRW.MSC; STRM.CHON.MRW.DR.MSC; FAT.ADIP.DR.MSC; FAT.MSC.DR.ADIP; MUS.SAT; SKIN.PEN.FRSK.FIB.01; SKIN.PEN.FRSK.FIB.02; SKIN.PEN.FRSK.KER.02; SKIN.PEN.FRSK.KER.03; BRST.HMEC.35; BRST.MYO; THYM; THYM.FET; BRN.HIPP.MID; BRN.ANT.CAUD; BRN.CING.GYR; BRN.DL.PRFRNTL.CRTX; FAT.ADIP.NUC; MUS.PSOAS;	ESDR.H1.NEUR.PROG; BLD.CD3.PPC; BLD.CD4.CD25I.CD127.TMEMPC; BLD.CD3.CPC; BLD.CD4.CD25.CD127M.TREGPC; BLD.CD4.CD25M.TPC; BLD.CD4.CD25M.CD45RA.NPC; BLD.CD4.CD25M.IL17M.PL.TPC; BLD.CD4.CD25M.IL17P.PL.TPC; BLD.CD4.CD25M.CD45RO.MPC; BLD.CD4.MPC; BLD.CD8.MPC; BLD.CD4.NPC; BLD.CD8.NPC; BLD.CD19.CPC; BLD.CD34.PC; BLD.MOB.CD34.PC.M; BLD.MOB.CD34.PC.F; BLD.CD34.CC; BLD.CD19.PPC; BLD.CD56.PC;	LNG.IMR90; ESC.H1; IPSC.DF.19.11; ESDR.H1.MSC; BLD.PER.MONUC.PC; BLD.CD3.PPC; BLD.CD4.CD25I.CD127.TMEMPC; BLD.CD4.CD25.CD127M.TREGPC; BLD.CD4.CD25M.TPC; BLD.CD4.CD25M.CD45RA.NPC; BLD.CD4.CD25M.IL17M.PL.TPC; BLD.CD4.CD25M.IL17P.PL.TPC; BLD.CD4.CD25M.CD45RO.MPC; BLD.CD4.MPC; BLD.CD8.MPC; BLD.CD4.NPC; BLD.CD8.NPC; BLD.CD14.PC; BLD.MOB.CD34.PC.F; BLD.CD19.PPC; BLD.CD56.PC; STRM.MRW.MSC; STRM.CHON.MRW.DR.MSC; SKIN.PEN.FRSK.FIB.01; SKIN.PEN.FRSK.FIB.02; SKIN.PEN.FRSK.KER.03; THYM; THYM.FET; BRN.HIPP.MID; BRN.SUB.NIG; BRN.ANT.CAUD; BRN.CING.GYR; BRN.INF.TMP; FAT.ADIP.NUC; MUS.PSOAS; MUS.SKLT.F; MUS.TRNK.FET; MUS.LEG.FET; HRT.ATR.R; HRT.VENT.L; HRT.VNT.R;	LNG.IMR90; ESC.HUES48; ESC.HUES64; IPSC.15b; ESDR.H1.NEUR.PROG; ESDR.H9.NEUR.PROG; BLD.PER.MONUC.PC; BLD.CD4.NPC; BLD.CD8.NPC; STRM.MRW.MSC; STRM.CHON.MRW.DR.MSC; FAT.ADIP.DR.MSC; FAT.MSC.DR.ADIP; MUS.SAT; BRN.ANT.CAUD; FAT.ADIP.NUC; MUS.SKLT.F; MUS.SKLT.M; HRT.FET; GI.RECT.SM.MUS; GI.STMC.MUS; GI.CLN.MUC; GI.RECT.MUC.29; GI.RECT.MUC.31; GI.STMC.MUC; GI.DUO.MUC; LNG.FET; LIV.ADLT	**H3K4me1**<br>BLD.DND41.CNCR; BLD.GM12878; CRVX.HELAS3.CNCR; LIV.HEPG2.CNCR; BRST.HMEC; MUS.HSMM; MUS.HSMMT; VAS.HUVEC; BLD.K562.CNCR; BLD.CD14.MONO; BRN.NHA; SKIN.NHDFAD; SKIN.NHEK; LNG.NHLF; BONE.OSTEO<br><br>**H3K27ac**<br>BLD.DND41.CNCR; BLD.GM12878;; LIV.HEPG2.CNCR;	**H3K4me3**<br>BLD.DND41.CNCR; BLD.GM12878; MUS.HSMMT; BLD.CD14.MONO; SKIN.NHEK<br><br>**H3K9ac**<br>BLD.DND41.CNCR; BLD.GM12878; LIV.HEPG2.CNCR; MUS.HSMMT; BLD.K562.CNCR; BLD.CD14.MONO; BRN.NHA; SKIN.NHEK; LNG.NHLF	**ROADMAP**<br>LNG.IMR90, ESDR.H1.MSC, BLD.CD3.PPC, BLD.CD3.CPC, BLD.CD14.PC, BLD.MOB.CD34.PC.M, BLD.MOB.CD34.PC.F, BLD.CD19.PPC, BLD.CD56.PC, SKIN.PEN.FRSK.FIB.01, SKIN.PEN.FRSK.FIB.02, THYM.FET, MUS.PSOAS, PLCNT.FET, PANC<br><br>**ENCODE**<br>BLD.GM12878, BRST.HMEC, MUS.HSMM, MUS.HSMMT, BLD.CD14.

	(ESDR.H1.NEUR.PROG; SKIN.PEN.FRSK.KER.03; BRST.MYO; BRN.HIPP.MID; BRN.DL.PRFRNTL.CRTX; MUS.PSOAS; MUS.TRNK.FET; GI.CLN.SM.MUS; GI.RECT.SM.MUS; PLCNT.AMN; LNG.FET)<br><br>**18_EnhW2**<br>(ESDR.CD56.MESO; BRN.GRM.MTRX)<br><br>**19_DNase**<br>(ESDR.CD184.ENDO; BRN.GANGEM.DR.NRSPHR; BRN.FET.F)<br><br>**3_PromD1**<br>(BLD.CD3.PPC; BLD.CD4.CD25I.CD127.TMEMPC; BLD.CD4.CD25.CD127M.TREGPC; BLD.CD4.CD25M.TPC; BLD.CD4.CD25M.CD45RA.NPC; BLD.CD4.CD25M.IL17M.PL.TPC; BLD.CD4.CD25M.IL17P.PL.TPC; BLD.CD4.CD25M.CD45RO.MPC; BLD.CD4.MPC; BLD.CD8.MPC; BLD.CD4.NPC; BLD.CD8.NPC; BLD.CD19.PPC; BLD.CD56.PC; BLD.CD15.PC; THYM.FET)<br><br>**2_PromD1**<br>(BLD.CD14.PC; BLD.CD19.CPC; BLD.MOB.CD34.PC.M; BLD.MOB.CD34.PC.F; BLD.CD34.CC)<br><br>**9_TxReg**<br>(THYM; MUS.SKLT.M; GI.DUO.SM.MUS; GI.STMC.MUS; GI.S.INT.FET; GI.DUO.MUC)<br><br>**10_TxEnh5**<br>(GI.STMC.FET)	BONE.OSTEO)	MUS.SKLT.F; MUS.SKLT.M; MUS.TRNK.FET; MUS.LEG.FET; HRT.ATR.R; HRT.VENT.L; HRT.VNT.R; GI.DUO.SM.MUS; GI.CLN.SM.MUS; GI.RECT.SM.MUS; GI.STMC.MUS; GI.STMC.FET; GI.S.INT.FET; GI.L.INT.FET; GI.S.INT; GI.CLN.SIG; GI.CLN.MUC; GI.RECT.MUC.29; GI.RECT.MUC.31; GI.STMC.MUC; GI.DUO.MUC; GI.ESO; GI.STMC.GAST; PLCNT.AMN; LNG.FET; PANC.ISLT; ADRL.GLND.FET; PLCNT.FET; LIV.ADLT; PANC; LNG; SPLN	BLD.CD15.PC; STRM.MRW.MSC; FAT.ADIP.DR.MSC; FAT.MSC.DR.ADIP; MUS.SAT; SKIN.PEN.FRSK.FIB.01; SKIN.PEN.FRSK.FIB.02; GI.DUO.SM.MUS; GI.RECT.SM.MUS; GI.STMC.MUS; GI.RECT.MUC.29; GI.STMC.MUC; GI.DUO.MUC; LIV.ADLT; SPLN	VAS.AOR; GI.DUO.SM.MUS; GI.RECT.SM.MUS; GI.STMC.MUS; GI.STMC.FET; GI.S.INT.FET; GI.L.INT.FET; GI.S.INT; GI.CLN.SIG; GI.CLN.MUC; GI.RECT.MUC.29; GI.RECT.MUC.31; GI.ESO; GI.STMC.GAST; PLCNT.AMN; ADRL.GLND.FET; PLCNT.FET; LIV.ADLT; PANC; LNG; SPLN		BRST.HMEC; MUS.HSMM; MUS.HSMMT;; BLD.CD14.MONO; BRN.NHA; SKIN.NHDFAD; SKIN.NHEK; LNG.NHLF; BONE.OSTEO		MONO, BRN.NHA, SKIN.NHDFAD, LNG.NHLF
rs72797303	**17_EnhW2**<br>(LNG.IMR90; IPSC.15b; ESDR.H1.NEUR.PROG; ESDR.H1.BMP4.TROP; ESDR.H1.MSC; STRM.MRW.MSC; MUS.SAT; SKIN.PEN.FRSK.FIB.02; SKIN.PEN.FRSK.MEL.01;	**17_EnhW2**<br><br>(LNG.A549.ETOH002.CNCR)<br><br>**9_TxReg**	LNG.IMR90; ESC.WA7; ESC.I3; ESC.HUES6; IPSC.20B; IPSC.DF.19.11; ESDR.CD56.MESO; ESDR.CD56.ECTO; ESDR.H1.BMP4.TROP; ESDR.H1.MSC; BLD.PER.MONUC.PC;	ESDR.H1.NEUR.PROG; ESDR.H1.MSC; BLD.CD4.CD25I.CD127.TMEMPC; BLD.CD4.CD25	ESDR.H1.MSC; BLD.PER.MONUC.PC; BLD.CD3.PPC; BLD.CD4.CD25I.CD127.TMEMPC; BLD.CD4.CD25.CD127M.TREGPC; BLD.CD4.CD25M.TPC;	LNG.IMR90; ESDR.H1.NEUR.PROG; BLD.PER.MONUC.PC; BLD.CD4.NPC; FAT.MSC.DR.AD	**H3K4me1**<br>LNG.A549.ETOH002.CNCR; BLD.DND41.CNCR; BLD.GM1	**H3K4me3**<br>BLD.DND41.CNCR; MUS.HSMMT;	DNase ROADMAP<br><br>ENCODE ESDR.H1.BMP4.MESO

	SKIN.PEN.FRSK.MEL.03; SKIN.PEN.FRSK.KER.02; SKIN.PEN.FRSK.KER.03; BRST.HMEC.35; BRST.MYO; BRN.SUB.NIG; BRN.ANT.CAUD; BRN.CING.GYR; BRN.INF.TMP; BRN.ANG.GYR; BRN.FET.M; MUS.PSOAS; MUS.TRNK.FET; VAS.AOR; GI.RECT.SM.MUS; GI.STMC.FET; PLCNT.AMN; KID.FET; LNG.FET; OVRY; PANC.ISLT)<br><br>**19_DNase**<br><br>(ESC.WA7; ESC.I3; ESC.HUES6; IPSC.18; ESDR.H9.NEUR.PROG; ESDR.CD184.ENDO)<br><br>**10_TxEnh5**<br>(BLD.PER.MONUC.PC; BLD.CD4.CD25I.CD127.TMEMPC; BLD.CD3.CPC; BLD.CD4.CD25M.TPC; BLD.CD4.CD25M.CD45RA.NPC; BLD.CD4.CD25M.CD45RO.MPC; BLD.CD4.NPC; BLD.CD8.NPC; BLD.CD34.PC; BLD.CD19.PPC; THYM; BRN.HIPP.MID; GI.DUO.SM.MUS; GI.S.INT.FET)<br><br>**9_TxReg**<br>(BLD.CD3.PPC; BLD.CD4.CD25.CD127M.TREGPC; BLD.CD4.CD25M.IL17M.PL.TPC; BLD.CD4.CD25M.IL17P.PL.TPC; BLD.CD4.MPC; BLD.CD8.MPC; BLD.CD14.PC; BLD.CD19.CPC; BLD.MOB.CD34.PC.M; BLD.MOB.CD34.PC.F; BLD.CD34.CC; BLD.CD56.PC; BLD.CD15.PC; THYM.FET; GI.DUO.MUC; ; SPLN)<br><br>**15_EnhAF**<br>(STRM.CHON.MRW.DR.MSC; FAT.ADIP.DR.MSC; FAT.MSC.DR.ADIP; SKIN.PEN.FRSK.FIB.01; MUS.SKLT.F; MUS.SKLT.M; HRT.FET; HRT.ATR.R;	(BLD.DND41.CNCR; BLD.CD14.MONO)<br><br>**10_TxEnh5**<br>(BLD.GM12878)<br><br>**15_EnhAF**<br>CRVX.HELAS3.CNCR; LIV.HEPG2.CNCR; MUS.HSMMT; BLD.K562.CNCR; BRN.NHA;<br><br>**17_EnhW2**<br>(BRST.HMEC; MUS.HSMM; VAS.HUVEC; SKIN.NHDFAD; SKIN.NHEK; LNG.NHLF; BONE.OSTEO)	BLD.CD3.PPC; BLD.CD4.CD25I.CD127.TMEMPC; BLD.CD3.CPC; BLD.CD4.CD25.CD127M.TREGPC; BLD.CD4.CD25M.TPC; BLD.CD4.CD25M.CD45RA.NPC; BLD.CD4.CD25M.IL17M.PL.TPC; BLD.CD4.CD25M.IL17P.PL.TPC; BLD.CD4.CD25M.CD45RO.MPC; BLD.CD4.MPC; BLD.CD8.MPC; BLD.CD4.NPC; BLD.CD8.NPC; BLD.CD14.PC; BLD.CD19.CPC; BLD.CD34.PC; BLD.MOB.CD34.PC.M; BLD.MOB.CD34.PC.F; BLD.CD34.CC; BLD.CD19.PPC; BLD.CD56.PC; BLD.CD15.PC; STRM.MRW.MSC; STRM.CHON.MRW.DR.MSC; FAT.ADIP.DR.MSC; FAT.MSC.DR.ADIP; SKIN.PEN.FRSK.FIB.01; SKIN.PEN.FRSK.FIB.02; SKIN.PEN.FRSK.KER.02; SKIN.PEN.FRSK.KER.03; BRST.HMEC.35; BRST.MYO; THYM; THYM.FET; BRN.HIPP.MID; BRN.SUB.NIG; BRN.ANT.CAUD; BRN.CING.GYR; BRN.INF.TMP; BRN.DL.PRFRNTL.CRTX; FAT.ADIP.NUC; MUS.PSOAS; MUS.SKLT.F; MUS.SKLT.M; MUS.TRNK.FET; MUS.LEG.FET; HRT.FET; HRT.ATR.R; HRT.VENT.L; HRT.VNT.R; GI.DUO.SM.MUS; GI.CLN.SM.MUS; GI.RECT.SM.MUS; GI.STMC.MUS; GI.STMC.FET; GI.S.INT.FET; GI.L.INT.FET; GI.S.INT; GI.CLN.SIG; GI.CLN.MUC; GI.RECT.MUC.29; GI.RECT.MUC.31; GI.STMC.MUC; GI.DUO.MUC; GI.ESO; GI.STMC.GAST; PLCNT.AMN; LNG.FET; ADRL.GLND.FET; PLCNT.FET; LIV.ADLT; PANC; LNG; SPLN	.CD127M.TREGPC; BLD.CD4.CD25M.CD45RA.NPC; BLD.CD4.CD25M.IL17M.PL.TPC; BLD.CD4.CD25M.IL17P.PL.TPC; BLD.CD4.CD25M.CD45RO.MPC; BLD.CD4.MPC; BLD.CD8.MPC; BLD.CD4.NPC; BLD.MOB.CD34.PC.M; BLD.MOB.CD34.PC.F; BLD.CD34.CC; BLD.CD15.PC; STRM.MRW.MSC; SKIN.PEN.FRSK.FIB.02; GI.DUO.SM.MUS; GI.RECT.SM.MUS; GI.STMC.MUS; GI.CLN.MUC; GI.RECT.MUC.29; GI.STMC.MUC ; GI.DUO.MUC; LIV.ADLT; PANC	BLD.CD4.CD25M.CD45RA.NPC; BLD.CD4.CD25M.IL17M.PL.TPC; BLD.CD4.CD25M.IL17P.PL.TPC; BLD.CD4.CD25M.CD45RO.MPC; BLD.CD4.MPC; BLD.CD8.MPC; BLD.CD4.NPC; BLD.CD8.NPC; BLD.CD14.PC; BLD.MOB.CD34.PC.F; BLD.CD19.PPC; BLD.CD56.PC; STRM.MRW.MSC; SKIN.PEN.FRSK.FIB.01; SKIN.PEN.FRSK.FIB.02; SKIN.PEN.FRSK.KER.03; THYM; THYM.FET; BRN.HIPP.MID; BRN.SUB.NIG; BRN.ANT.CAUD; BRN.CING.GYR; BRN.INF.TMP; FAT.ADIP.NUC; MUS.PSOAS; MUS.SKLT.F; HRT.ATR.R; HRT.VENT.L; HRT.VNT.R; GI.DUO.SM.MUS; GI.RECT.SM.MUS; GI.STMC.MUS; GI.STMC.FET; GI.S.INT.FET; GI.L.INT.FET; GI.S.INT; GI.CLN.SIG; GI.CLN.MUC; GI.RECT.MUC.29; GI.RECT.MUC.31; GI.ESO; GI.STMC.GAST; PLCNT.FET; LIV.ADLT; LNG; SPLN	IP; FAT.ADIP.NUC; MUS.SKLT.M; HRT.FET; GI.STMC.MUS; GI.CLN.MUC; GI.RECT.MUC.29; GI.RECT.MUC.31; GI.STMC.MUC; GI.DUO.MUC; LIV.ADLT.	2878; CRVX.HELAS3.CNCR ; LIV.HEPG2.CNCR; BRST.HMEC; MUS.HSMM; MUS.HSMMT; BLD.K562.CNCR; BLD.CD14.MONO; BRN.NHA; SKIN.NHDFAD; SKIN.NHEK; LNG.NHLF ; BONE.OSTEO<br><br>**H3K27ac**<br>BLD.DND41.CNCR; BLD.GM12878; CRVX.HELAS3.CNCR ; MUS.HSMMT; BLD.K562.CNCR; BLD.CD14.MONO; SKIN.NHEK; BONE.OSTEO	BLD.CD14.MONO<br><br>**H3K9ac**<br>BLD.DND41.CNCR ; BLD.CD14.MONO ; BRN.NHA; LNG.NHLF.	; BLD.CD3.PPC; BLD.CD14.PC; LNG.A549.ETOH002.CNCR; BLD.GM12878; MUS.HSMMT; BLD.CD14.MONO

	HRT.VENT.L; HRT.VNT.R; GI.CLN.SM.MUS; GI.L.INT.FET; GI.S.INT; GI.CLN.SIG; GI.RECT.MUC.29; GI.RECT.MUC.31; GI.ESO; GI.STMC.GAST, PLCNT.FET; PANC; LNG)<br><br>**11_TxEnh3**<br>(BRN.DL.PRFRNTL.CRTX; MUS.LEG.FET; GI.STMC.MUS; ADRL.GLND.FET)<br><br>**14_EnhA2**<br>(FAT.ADIP.NUC; GI.CLN.MUC; GI.STMC.MUC)<br><br>**13_EnhA1**<br>(LIV.ADLT)								
rs2706396	**12_TxEnhW**<br>(BLD.PER.MONUC.PC; BLD.CD3.CPC; THYM; BRN.HIPP.MID)<br><br>**9_TxReg**<br>(BLD.CD3.PPC; BLD.CD56.PC; BLD.CD15.PC; GI.DUO.MUC; LIV.ADLT)<br><br>**10_TxEnh5**<br>(BLD.CD4.CD25I.CD127.TMEMPC; BLD.CD4.CD25.CD127M.TREGPC; BLD.CD4.CD25M.TPC; BLD.CD4.CD25M.CD45RA.NPC; BLD.CD4.CD25M.IL17M.PL.TPC; BLD.CD4.CD25M.IL17P.PL.TPC; BLD.CD4.CD25M.CD45RO.MPC; BLD.CD4.MPC; BLD.CD8.MPC; BLD.CD4.NPC; BLD.CD8.NPC; BLD.CD14.PC; BLD.CD19.CPC; BLD.CD34.PC; BLD.MOB.CD34.PC.M; BLD.MOB.CD34.PC.F; BLD.CD34.CC; BLD.CD19.PPC; THYM.FET; GI.DUO.SM.MUS; GI.S.INT.FET; GI.S.INT; GI.RECT.MUC.29)<br><br>**17_EmhW2**<br>(BRST.MYO; BRN.SUB.NIG; BRN.ANT.CAUD; BRN.CING.GYR; BRN.INF.TMP; MUS.SKLT.F; MUS.SKLT.M; MUS.LEG.FET; HRT.VNT.R; GI.RECT.SM.MUS; GI.STMC.MUS;	**13_EnhA1**<br>(BLD.DND41.CNCR)<br><br>**10_TxEnhW2**<br>(BLD.GM12878)<br><br>**17_EnhW2**<br>(CRVX.HELAS3.CNCR; BLD.K562.CNCR)<br><br>**15_EnhAF**<br>(LIV.HEPG2.CNCR)<br><br>**9_TxReg**<br>(BLD.CD14.MONO)	LNG.IMR90; IPSC.DF.19.11; ESDR.CD56.MESO; ESDR.CD56.ECTO; ESDR.H1.BMP4.TROP; ESDR.H1.MSC; BLD.PER.MONUC.PC; BLD.CD3.PPC; BLD.CD4.CD25I.CD127.TMEMPC; BLD.CD3.CPC; BLD.CD4.CD25.CD127M.TREGPC; BLD.CD4.CD25M.TPC; BLD.CD4.CD25M.CD45RA.NPC; BLD.CD4.CD25M.IL17M.PL.TPC; BLD.CD4.CD25M.IL17P.PL.TPC; BLD.CD4.CD25M.CD45RO.MPC; BLD.CD4.MPC; BLD.CD8.MPC; BLD.CD4.NPC; BLD.CD8.NPC; BLD.CD14.PC; BLD.CD19.CPC; BLD.CD34.PC; BLD.MOB.CD34.PC.M; BLD.MOB.CD34.PC.F; BLD.CD34.CC; BLD.CD19.PPC; BLD.CD56.PC; BLD.CD15.PC; STRM.MRW.MSC; SKIN.PEN.FRSK.FIB.01; SKIN.PEN.FRSK.FIB.02; SKIN.PEN.FRSK.KER.03; BRST.MYO; BRN.GANGEM.DR.NRSPHR; THYM; THYM.FET; BRN.HIPP.MID; BRN.SUB.NIG; BRN.ANT.CAUD;	BLD.CD4.CD25I.CD127.TMEMPC; BLD.CD4.CD25M.IL17P.PL.TPC; BLD.CD4.CD25M.CD45RO.MPC; BLD.CD4.MPC; BLD.CD8.MPC; BLD.MOB.CD34.PC.M; BLD.MOB.CD34.PC.F; BLD.CD15.PC; GI.DUO.SM.MUS; GI.CLN.SM.MUS; GI.RECT.SM.MUS; GI.STMC.MUS; GI.RECT.MUC.29; GI.STMC.MUC; GI.DUO.MUC; LIV.ADLT; PANC.	ESDR.H1.MSC; BLD.PER.MONUC.PC; BLD.CD3.PPC; BLD.CD4.CD25I.CD127.TMEMPC; BLD.CD4.CD25.CD127M.TREGPC; BLD.CD4.CD25M.TPC; BLD.CD4.CD25M.CD45RA.NPC; BLD.CD4.CD25M.IL17M.PL.TPC; BLD.CD4.CD25M.IL17P.PL.TPC; BLD.CD4.CD25M.CD45RO.MPC; BLD.CD4.MPC; BLD.CD8.MPC; BLD.CD4.NPC; BLD.CD8.NPC; BLD.CD14.PC; BLD.MOB.CD34.PC.F; BLD.CD19.PPC; BLD.CD56.PC; SKIN.PEN.FRSK.FIB.01; THYM; THYM.FET; BRN.HIPP.MID; BRN.ANT.CAUD; BRN.CING.GYR; FAT.ADIP.NUC; MUS.PSOAS; MUS.SKLT.F; HRT.ATR.R; HRT.VNT.R; GI.DUO.SM.MUS; GI.RECT.SM.MUS;	ESDR.H1.NEUR.PROG; BLD.PER.MONUC.PC; BLD.CD4.NPC; BRN.ANG.GYR; FAT.ADIP.NUC; MUS.SKLT.M; HRT.FET; GI.CLN.SM.MUS; GI.STMC.MUS; GI.CLN.MUC; GI.RECT.MUC.29; GI.RECT.MUC.31; GI.STMC.MUC; GI.DUO.MUC; LNG.FET; LIV.ADLT.	**H3K4me1**<br>LNG.A549.ETOH002.CNCR; BLD.DND41.CNCR; BLD.GM12878; CRVX.HELAS3.CNCR; LIV.HEPG2.CNCR; BRST.HMEC; MUS.HSMMT; BLD.CD14.MONO; LNG.NHLF.<br><br>**H3K27ac**<br>BLD.DND41.CNCR; BLD.CD14.MONO; BONE.OSTEO	**H3K4me3**<br><br>BLD.CD14.MONO.<br><br>**H3K9ac**<br>BLD.DND41.CNCR; BLD.CD14.MONO.	

	GI.CLN.MUC; GI.ESO; GI.STMC.GAST; KID.FET; LNG.FET; ADRL.GLND.FET; PANC; LNG; SPLN)<br><br>**15_EnhAF** (FAT.ADIP.NUC; GI.CLN.SM.MUS; GI.L.INT.FET; GI.CLN.SIG; GI.RECT.MUC.31; GI.STMC.MUC)<br><br>**11_TxEnh3** (GI.STMC.FET; PLCNT.FET)		BRN.CING.GYR; BRN.DL.PRFRNTL.CRTX; FAT.ADIP.NUC; MUS.SKLT.F; MUS.SKLT.M; MUS.TRNK.FET; MUS.LEG.FET; HRT.FET; HRT.ATR.R; HRT.VENT.L; HRT.VNT.R; GI.DUO.SM.MUS; GI.CLN.SM.MUS; GI.RECT.SM.MUS; GI.STMC.MUS; GI.STMC.FET; GI.S.INT.FET; GI.L.INT.FET; GI.S.INT; GI.CLN.SIG; GI.CLN.MUC; GI.RECT.MUC.29; GI.RECT.MUC.31; GI.STMC.MUC; GI.DUO.MUC; GI.ESO; GI.STMC.GAST; LNG.FET; ADRL.GLND.FET; PLCNT.FET; LIV.ADLT; PANC; LNG; SPLN		GI.STMC.MUS; GI.STMC.FET; GI.S.INT.FET; GI.L.INT.FET; GI.S.INT; GI.CLN.SIG; GI.CLN.MUC; GI.RECT.MUC.29; GI.RECT.MUC.31; GI.ESO; GI.STMC.GAST; PLCNT.FET; LIV.ADLT; LNG.				
rs2522052	**17_EnhW2** (ESDR.H1.BMP4.TROP; SKIN.PEN.FRSK.FIB.02; SKIN.PEN.FRSK.MEL.01; BRST.MYO; BRN.CRTX.DR.NRSPHR; BRN.SUB.NIG; BRN.ANT.CAUD; BRN.CING.GYR; BRN.INF.TMP; BRN.ANG.GYR; BRN.DL.PRFRNTL.CRTX; BRN.GRM.MTRX; BRN.FET.M; MUS.PSOAS; HRT.ATR.R; GI.RECT.SM.MUS; GI.ESO; PLCNT.AMN; KID.FET; LNG.FET; OVRY; PANC.ISLT)<br><br>**10_TxEnh5** (BLD.PER.MONUC.PC; BLD.CD3.PPC; BLD.CD4.CD25I.CD127.TMEMPC; BLD.CD4.CD25.CD127M.TREGPC; BLD.CD4.CD25M.TPC; BLD.CD4.CD25M.CD45RA.NPC; BLD.CD4.CD25M.IL17M.PL.TPC; BLD.CD4.CD25M.IL17P.PL.TPC; BLD.CD4.CD25M.CD45RO.MPC; BLD.CD4.MPC; BLD.CD8.MPC; BLD.CD4.NPC; BLD.CD8.NPC; BLD.CD14.PC; BLD.CD19.CPC; BLD.CD34.PC; BLD.CD19.PPC; BLD.CD56.PC; THYM; THYM.FET; MUS.LEG.FET; HRT.VNT.R; GI.DUO.SM.MUS; GI.CLN.SM.MUS;	**9_TxReg** (BLD.DND41.CNCR)<br><br>**10_TxEnh5** (BLD.GM12878)<br><br>**15_EnhAF** (LIV.HEPG2.CNCR)<br><br>**15_EnhAF** (BLD.K562.CNCR)<br><br>**10_TxEnhAF** (BLD.CD14.MONO)	ESC.4STAR; IPSC.20B; IPSC.15b; IPSC.DF.19.11; ESDR.CD56.ECTO; ESDR.H1.MSC; BLD.PER.MONUC.PC; BLD.CD3.PPC; BLD.CD4.CD25I.CD127.TMEMPC; BLD.CD3.CPC; BLD.CD4.CD25.CD127M.TREGPC; BLD.CD4.CD25M.TPC; BLD.CD4.CD25M.CD45RA.NPC; BLD.CD4.CD25M.IL17M.PL.TPC; BLD.CD4.CD25M.IL17P.PL.TPC; BLD.CD4.CD25M.CD45RO.MPC; BLD.CD4.MPC; BLD.CD8.MPC; BLD.CD4.NPC; BLD.CD8.NPC; BLD.CD14.PC; BLD.CD19.CPC; BLD.CD34.PC; BLD.MOB.CD34.PC.M; BLD.MOB.CD34.PC.F; BLD.CD34.CC; BLD.CD19.PPC; BLD.CD56.PC; BLD.CD15.PC; SKIN.PEN.FRSK.FIB.02; BRST.MYO; THYM; THYM.FET; BRN.HIPP.MID; BRN.SUB.NIG; BRN.ANT.CAUD; BRN.INF.TMP; BRN.ANG.GYR; BRN.DL.PRFRNTL.CRTX; BRN.FET.F; BRN.FET.M; FAT.ADIP.NUC; MUS.SKLT.F;	BLD.CD4.CD25I.CD127.TMEMPC; BLD.CD4.CD25.CD127M.TREGPC; BLD.CD4.CD25M.IL17M.PL.TPC; BLD.CD4.CD25M.IL17P.PL.TPC; BLD.CD4.MPC; BLD.MOB.CD34.PC.F; BLD.CD15.PC; SKIN.PEN.FRSK.FIB.01; SKIN.PEN.FRSK.FIB.02; GI.DUO.SM.MUS; GI.CLN.SM.MUS; GI.RECT.SM.MUS; GI.CLN.MUC; GI.DUO.MUC;	BLD.PER.MONUC.PC; BLD.CD3.PPC; BLD.CD4.CD25I.CD127.TMEMPC; BLD.CD4.CD25.CD127M.TREGPC; BLD.CD4.CD25M.TPC; BLD.CD4.CD25M.CD45RA.NPC; BLD.CD4.CD25M.IL17M.PL.TPC; BLD.CD4.CD25M.IL17P.PL.TPC; BLD.CD4.CD25M.CD45RO.MPC; BLD.CD4.MPC; BLD.CD8.MPC; BLD.CD4.NPC; BLD.CD8.NPC; BLD.MOB.CD34.PC.F; BLD.CD19.PPC; BLD.CD56.PC; SKIN.PEN.FRSK.FIB.02; THYM; THYM.FET; BRN.HIPP.MID; BRN.SUB.NIG; BRN.ANT.CAUD; BRN.CING.GYR; BRN.INF.TMP; BRN.DL.PRFRNTL.CRTX; FAT.ADIP.NUC; MUS.PSOAS;	ESC.HUES6; BRN.SUB.NIG; BRN.ANT.CAUD; BRN.INF.TMP; BRN.ANG.GYR; FAT.ADIP.NUC; GI.CLN.SM.MUS; GI.RECT.SM.MUS; GI.CLN.MUC; GI.RECT.MUC.29; GI.RECT.MUC.31; GI.STMC.MUC; GI.DUO.MUC; GI.ESO; LNG.FET; PANC.ISLT; LIV.ADLT.	**H3K4me1** BLD.DND41.CNCR; BLD.GM12878; LIV.HEPG2.CNCR; MUS.HSMM; MUS.HSMMT; BLD.CD14.MONO.<br><br>**H3K27ac** BLD.DND41.CNCR; BLD.GM12878; BLD.CD14.MONO.	**H3K4me3** BLD.DND41.CNCR; BLD.K562.CNCR; BLD.CD14.MONO; BRN.NHA; SKIN.NHDFAD; SKIN.NHEK; LNG.NHLF; BONE.OSTEO<br><br>**H3K9ac** BLD.DND41.CNCR.	

	GI.STMC.FET; GI.CLN.SIG; GI.RECT.MUC.29; SPLN)<br><br>**12_TxEnhW**<br>(BLD.CD3.CPC; SKIN.PEN.FRSK.FIB.01; BRN.HIPP.MID; MUS.SKLT.M)<br><br>**9_TxReg**<br>(BLD.MOB.CD34.PC.M; BLD.MOB.CD34.PC.F; BLD.CD34.CC; BLD.CD15.PC; GI.S.INT.FET; GI.DUO.MUC)<br><br>**15_EnhW2**<br>(FAT.ADIP.NUC; MUS.SKLT.F; MUS.TRNK.FET; HRT.VENT.L; GI.S.INT; GI.RECT.MUC.31; GI.STMC.MUC; ADRL.GLND.FET; PANC; LNG)<br><br>**11_TxEnh3**<br>(GI.STMC.MUS; GI.STMC.GAST; PLCNT.FET)<br><br>**13_EnhA1**<br>(GI.L.INT.FET; LIV.ADLT)<br><br>**14_EnhA2**<br>(GI.CLN.MUC)		MUS.SKLT.M; MUS.TRNK.FET; MUS.LEG.FET; HRT.ATR.R; HRT.VENT.L; HRT.VNT.R; GI.DUO.SM.MUS; GI.CLN.SM.MUS; GI.RECT.SM.MUS; GI.STMC.MUS; GI.STMC.FET; GI.S.INT.FET; GI.L.INT.FET; GI.S.INT; GI.CLN.SIG; GI.CLN.MUC; GI.RECT.MUC.29; GI.RECT.MUC.31; GI.STMC.MUC; GI.DUO.MUC; GI.ESO; GI.STMC.GAST; KID.FET; LNG.FET; PANC.ISLT; PLCNT.FET; LIV.ADLT; PANC; LNG; SPLN	LIV.ADLT; SPLN	MUS.SKLT.F; MUS.LEG.FET; HRT.VENT.L; HRT.VNT.R; GI.DUO.SM.MUS; GI.CLN.SM.MUS; GI.STMC.MUS; GI.STMC.FET; GI.S.INT.FET; GI.L.INT.FET; GI.S.INT; GI.CLN.SIG; GI.CLN.MUC; GI.RECT.MUC.29; GI.RECT.MUC.31; GI.ESO; GI.STMC.GAST; PLCNT.FET; LIV.ADLT; LNG; SPLN				
rs2706403	**17_EnhW2**<br>(ESDR.H1.NEUR.PROG; ESDR.CD56.ECTO; ESDR.H1.BMP4.TROP; SKIN.PEN.FRSK.FIB.01; SKIN.PEN.FRSK.FIB.02; SKIN.PEN.FRSK.MEL.01; SKIN.PEN.FRSK.MEL.03; BRST.MYO; BRN.CRTX.DR.NRSPHR; BRN.SUB.NIG; BRN.ANT.CAUD; BRN.INF.TMP; BRN.ANG.GYR; BRN.DL.PRFRNTL.CRTX; BRN.GRM.MTRX; BRN.FET.M; MUS.PSOAS; HRT.FET; HRT.ATR.R; PLCNT.AMN; KID.FET; OVRY; PANC.ISLT; LNG)<br><br>**18_EnhAc**<br>(ESDR.H1.MSC; BRN.GANGEM.DR.NRSPHR;	**9_TxReg**<br>(BLD.DND41.CNCR; BLD.CD14.MONO)<br><br>**10_TxEnh5**<br><br>(BLD.GM12878)<br><br>**17_EnhW2**<br>(CRVX.HELAS3.CNCR)<br><br>**14_EnhW2**<br>(LIV.HEPG2.CNCR)	ESC.H1; ESC.4STAR; IPSC.20B; IPSC.15b; IPSC.DF.19.11; ESDR.CD56.MESO; ESDR.CD56.ECTO; ESDR.H1.MSC; BLD.PER.MONUC.PC; BLD.CD3.PPC; BLD.CD4.CD25I.CD127.TMEMPC; BLD.CD3.CPC; BLD.CD4.CD25.CD127M.TREGPC; BLD.CD4.CD25M.TPC; BLD.CD4.CD25M.CD45RA.NPC; BLD.CD4.CD25M.IL17M.PL.TPC; BLD.CD4.CD25M.IL17P.PL.TPC; BLD.CD4.CD25M.CD45RO.MPC; BLD.CD4.MPC; BLD.CD8.MPC; BLD.CD4.NPC; BLD.CD8.NPC; BLD.CD14.PC; BLD.CD19.CPC; BLD.CD34.PC; BLD.MOB.CD34.PC.M;	ESDR.CD56.ECTO; BLD.CD4.CD25I.CD127.TMEMPC; BLD.CD4.CD25.CD127M.TREGPC; BLD.CD4.CD25M.TPC; BLD.CD4.CD25M.IL17M.PL.TPC; BLD.CD4.CD25M.IL17P.PL.TPC; BLD.CD4.CD25M.CD45RO.MPC;	IPSC.20B; BLD.PER.MONUC.PC; BLD.CD3.PPC; BLD.CD4.CD25I.CD127.TMEMPC; BLD.CD4.CD25.CD127M.TREGPC; BLD.CD4.CD25M.TPC; BLD.CD4.CD25M.CD45RA.NPC; BLD.CD4.CD25M.IL17M.PL.TPC; BLD.CD4.CD25M.IL17P.PL.TPC; BLD.CD4.CD25M.CD45RO.MPC; BLD.CD4.MPC; BLD.CD8.MPC; BLD.CD4.NPC; BLD.CD8.NPC; BLD.MOB.CD34.PC.F;	ESC.HUES6; BLD.PER.MONUC.PC; BRN.SUB.NIG; BRN.ANT.CAUD; BRN.CING.GYR; BRN.INF.TMP; BRN.ANG.GYR; BRN.DL.PRFRNTL.CRTX; FAT.ADIP.NUC; MUS.SKLT.F; MUS.SKLT.M; GI.CLN.SM.MUS; GI.RECT.SM.MUS; GI.STMC.MUS;	**H3K4me1**<br>LNG.A549.ETOH002.CNCR; BLD.DND41.CNCR; BLD.GM12878; LIV.HEPG2.CNCR; MUS.HSMM; MUS.HSMMT; VAS.HUVEC; BLD.CD14.MONO.	**H3K4me3**<br>BLD.DND41.CNCR; LIV.HEPG2.CNCR; BLD.CD14.MONO.<br><br>**H3K9ac**<br>BLD.DND41.CNCR	ROADMAP<br>GI.S.INT.FET; GI.L.INT.FET; GI.S.INT;<br><br>ENCODE<br><br>NONE

	10_TxEnh5<br>BLD.PER.MONUC.PC; BLD.CD3.CPC; BLD.CD4.CD25.CD127M.TREGPC; BLD.CD4.CD25M.CD45RA.NPC; BLD.CD8.NPC; BLD.CD14.PC; BLD.CD34.PC; BLD.CD19.PPC; THYM; GI.DUO.SM.MUS; GI.CLN.SIG; SPLN)<br><br>**9_TxReg**<br>(BLD.CD3.PPC; BLD.CD4.CD25I.CD127.TMEMPC; BLD.CD4.CD25M.TPC; GI.RECT.MUC.29; BLD.CD4.CD25M.IL17M.PL.TPC; BLD.CD4.CD25M.IL17P.PL.TPC; BLD.CD4.CD25M.CD45RO.MPC; BLD.CD4.MPC; BLD.CD8.MPC; BLD.CD4.NPC; BLD.CD19.CPC; BLD.MOB.CD34.PC.M; BLD.MOB.CD34.PC.F; BLD.CD34.CC; BLD.CD56.PC; BLD.CD15.PC; THYM.FET; GI.S.INT.FET; GI.DUO.MUC)<br><br>**15_EnhAF**<br>(BRN.HIPP.MID; BRN.CING.GYR; FAT.ADIP.NUC; MUS.SKLT.F; MUS.SKLT.M; MUS.TRNK.FET; MUS.LEG.FET; HRT.VENT.L; HRT.VNT.R; GI.CLN.SM.MUS; GI.RECT.SM.MUS; GI.STMC.MUS; GI.STMC.FET; GI.STMC.MUC; GI.ESO; GI.STMC.GAST; LNG.FET; ADRL.GLND.FET; PLCNT.FET; LIV.ADLT; PANC)<br><br>**19_DNase**<br>(VAS.AOR)<br><br>**13_EnhA1**<br>(GI.L.INT.FET; GI.S.INT; GI.CLN.MUC)<br><br>**14_EnhA2**<br>(GI.RECT.MUC.31)	**15_EnhAF**<br>(BLD.K562.CNCR)	BLD.MOB.CD34.PC.F; BLD.CD34.CC; BLD.CD19.PPC; BLD.CD56.PC; BLD.CD15.PC; SKIN.PEN.FRSK.FIB.01; SKIN.PEN.FRSK.FIB.02; SKIN.PEN.FRSK.MEL.01; BRST.MYO; BRN.CRTX.DR.NRSPHR; THYM; THYM.FET; BRN.HIPP.MID; BRN.SUB.NIG; BRN.ANT.CAUD; BRN.CING.GYR; BRN.INF.TMP; BRN.ANG.GYR; BRN.DL.PRFRNTL.CRTX; BRN.FET.F; BRN.FET.M; FAT.ADIP.NUC; MUS.PSOAS; MUS.SKLT.F; MUS.SKLT.M; MUS.TRNK.FET; MUS.LEG.FET; HRT.ATR.R; HRT.VENT.L; HRT.VNT.R; GI.DUO.SM.MUS; GI.CLN.SM.MUS; GI.RECT.SM.MUS; GI.STMC.MUS; GI.STMC.FET; GI.S.INT.FET; GI.L.INT.FET; GI.S.INT; GI.CLN.SIG; GI.CLN.MUC; GI.RECT.MUC.29; GI.RECT.MUC.31; GI.STMC.MUC; GI.DUO.MUC; GI.ESO; GI.STMC.GAST; PLCNT.AMN; KID.FET; LNG.FET; PANC.ISLT; ADRL.GLND.FET; PLCNT.FET; LIV.ADLT; PANC; LNG; SPLN	BLD.CD4.MPC; BLD.CD8.MPC; BLD.CD8.NPC; BLD.MOB.CD34.PC.M; BLD.MOB.CD34.PC.F; BLD.CD19.PPC; BLD.CD15.PC; MUS.SAT; SKIN.PEN.FRSK.FIB.01; SKIN.PEN.FRSK.FIB.02; BRN.ANT.CAUD; BRN.CING.GYR; GI.DUO.SM.MUS; GI.CLN.SM.MUS; GI.RECT.SM.MUS; GI.STMC.MUS; GI.CLN.MUC; GI.RECT.MUC.29; GI.RECT.MUC.31; GI.STMC.MUC; GI.DUO.MUC; LIV.ADLT; SPLN	BLD.CD19.PPC; BLD.CD56.PC; SKIN.PEN.FRSK.FIB.02; THYM; THYM.FET; BRN.HIPP.MID; BRN.SUB.NIG; BRN.ANT.CAUD; BRN.CING.GYR; BRN.INF.TMP; BRN.ANG.GYR; BRN.DL.PRFRNTL.CRTX; FAT.ADIP.NUC; MUS.PSOAS; MUS.SKLT.F; MUS.TRNK.FET; MUS.LEG.FET; HRT.VENT.L; HRT.VNT.R; GI.DUO.SM.MUS; GI.CLN.SM.MUS; GI.RECT.SM.MUS; GI.STMC.MUS; GI.STMC.FET; GI.S.INT.FET; GI.L.INT.FET; GI.S.INT; GI.CLN.SIG; GI.CLN.MUC; GI.RECT.MUC.29; GI.RECT.MUC.31; GI.ESO; GI.STMC.GAST; ADRL.GLND.FET; PLCNT.FET; LIV.ADLT; LNG; SPLN	GI.CLN.MUC; GI.RECT.MUC.29; GI.RECT.MUC.31; GI.STMC.MUC; GI.DUO.MUC; LNG.FET; PANC.ISLT; LIV.ADLT.	**H3K27ac**<br>BLD.DND41.CNCR; BLD.GM12878; CRVX.HELAS3.CNCR; BRST.HMEC; BLD.CD14.MONO.	; LIV.HEPG2.CNCR; MUS.HSMMT.	
rs2706336	**12_TxEnhW**<br>(BLD.PER.MONUC.PC; BLD.CD3.CPC; THYM; BRN.HIPP.MID)<br><br>**9_TxReg**	**13_EnhA1**<br>9BLD.DND41.CNCR)<br><br>**10_TxEnh5**	LNG.IMR90; IPSC.DF.19.11; ESDR.CD56.MESO; ESDR.CD56.ECTO; ESDR.H1.BMP4.TROP; ESDR.H1.MSC; BLD.PER.MONUC.PC;	BLD.CD4.CD25I.CD127.TMEMPC; BLD.CD4.CD25M.IL17P.PL.TPC;	ESDR.H1.MSC; BLD.PER.MONUC.PC; BLD.CD3.PPC; BLD.CD4.CD25I.CD127.TMEMPC; BLD.CD4.CD25.CD127M.TRE	ESDR.H1.NEUR.PROG; BLD.PER.MONUC.PC; BLD.CD4.NPC; BRN.ANG.GYR;	**H3K4me1**<br>LNG.A549.ETOH002.CNCR; BLD.DND41.CNCR;	**H3K4me3**<br>BLD.CD14.MONO	DNase ROADMAP GI.S.INT.FET, GI.L.INT.FET

	(BLD.CD3.PPC; BLD.CD56.PC; BLD.CD15.PC; GI.DUO.MUC; LIV.ADLT)<br><br>**10_TxEnhW**<br>(BLD.CD4.CD25I.CD127.TMEMPC; BLD.CD4.CD25.CD127M.TREGPC; BLD.CD4.CD25M.TPC; BLD.CD4.CD25M.CD45RA.NPC; BLD.CD4.CD25M.IL17M.PL.TPC; BLD.CD4.CD25M.IL17P.PL.TPC; BLD.CD4.CD25M.CD45RO.MPC; BLD.CD4.MPC; BLD.CD8.MPC; BLD.CD4.NPC; BLD.CD8.NPC; BLD.CD14.PC; BLD.CD19.CPC; BLD.CD34.PC; BLD.MOB.CD34.PC.M; BLD.MOB.CD34.PC.F; BLD.CD34.CC; BLD.CD19.PPC; THYM.FET; GI.DUO.SM.MUS; GI.S.INT.FET; GI.S.INT; GI.RECT.MUC.29)<br><br>**17_EnhW2**<br>(BRST.MYO; BRN.SUB.NIG; BRN.ANT.CAUD; BRN.CING.GYR; BRN.INF.TMP; MUS.SKLT.F; MUS.LEG.FET; MUS.SKLT.M; HRT.VNT.R; GI.RECT.SM.MUS; GI.STMC.MUS; GI.CLN.MUC; GI.ESO; GI.STMC.GAST; KID.FET; LNG.FET; ADRL.GLND.FET; PANC; LNG; SPLN)<br><br>**15_EnhAF**<br>(FAT.ADIP.NUC; GI.CLN.SM.MUS; GI.L.INT.FET; GI.CLN.SIG; GI.RECT.MUC.31; GI.STMC.MUC)<br><br>**11_TxEnh3**<br>(GI.STMC.FET; PLCNT.FET)	(BLD.GM12878)<br><br>**17_EnhW2**<br>(CRVX.HELAS3.CNCR; BLD.K562.CNCR)<br><br>**15_EnhAF**<br>(LIV.HEPG2.CNCR)<br><br>**9_TxReg**<br>(BLD.CD14.MONO)	BLD.CD3.PPC; BLD.CD4.CD25I.CD127.TMEMPC; BLD.CD3.CPC; BLD.CD4.CD25.CD127M.TREGPC; BLD.CD4.CD25M.TPC; BLD.CD4.CD25M.CD45RA.NPC; BLD.CD4.CD25M.IL17M.PL.TPC; BLD.CD4.CD25M.IL17P.PL.TPC; BLD.CD4.CD25M.CD45RO.MPC; BLD.CD4.MPC; BLD.CD8.MPC; BLD.CD4.NPC; BLD.CD8.NPC; BLD.CD14.PC; BLD.CD19.CPC; BLD.CD34.PC; BLD.MOB.CD34.PC.M; BLD.MOB.CD34.PC.F; BLD.CD34.CC; BLD.CD19.PPC; BLD.CD56.PC; BLD.CD15.PC; STRM.MRW.MSC; SKIN.PEN.FRSK.FIB.01; SKIN.PEN.FRSK.FIB.02; SKIN.PEN.FRSK.KER.03; BRST.MYO; BRN.GANGEM.DR.NRSPHR; THYM; THYM.FET; BRN.HIPP.MID; BRN.SUB.NIG; BRN.ANT.CAUD; BRN.CING.GYR; BRN.DL.PRFRNTL.CRTX; FAT.ADIP.NUC; MUS.SKLT.F; MUS.SKLT.M; MUS.TRNK.FET; MUS.LEG.FET; HRT.FET; HRT.ATR.R; HRT.VENT.L; HRT.VNT.R; GI.DUO.SM.MUS; GI.CLN.SM.MUS; GI.RECT.SM.MUS; GI.STMC.MUS; GI.STMC.FET; GI.S.INT.FET; GI.L.INT.FET; GI.S.INT; GI.CLN.SIG; GI.CLN.MUC; GI.RECT.MUC.29; GI.RECT.MUC.31; GI.STMC.MUC; GI.DUO.MUC; GI.ESO; GI.STMC.GAST; LNG.FET; ADRL.GLND.FET; PLCNT.FET; LIV.ADLT; PANC; LNG; SPLN	BLD.CD4.CD25M.CD45RO.MPC; BLD.CD4.MPC; BLD.CD8.MPC; BLD.MOB.CD34.PC.M; BLD.MOB.CD34.PC.F; BLD.CD15.PC; GI.DUO.SM.MUS; GI.CLN.SM.MUS; GI.RECT.SM.MUS; GI.RECT.MUC.29; GI.STMC.MUC; GI.DUO.MUC; LIV.ADLT; PANC	GPC; BLD.CD4.CD25M.TPC; BLD.CD4.CD25M.CD45RA.NPC; BLD.CD4.CD25M.IL17M.PL.TPC; BLD.CD4.CD25M.IL17P.PL.TPC; BLD.CD4.CD25M.CD45RO.MPC; BLD.CD4.MPC; BLD.CD8.MPC; BLD.CD4.NPC; BLD.CD8.NPC; BLD.CD14.PC; BLD.MOB.CD34.PC.F; BLD.CD19.PPC; BLD.CD56.PC; SKIN.PEN.FRSK.FIB.01; THYM; THYM.FET; BRN.HIPP.MID; BRN.ANT.CAUD; BRN.CING.GYR; FAT.ADIP.NUC; MUS.PSOAS; MUS.SKLT.F; HRT.ATR.R; HRT.VNT.R; GI.DUO.SM.MUS; GI.RECT.SM.MUS; GI.STMC.MUS; GI.STMC.FET; GI.S.INT.FET; GI.L.INT.FET; GI.S.INT; GI.CLN.SIG; GI.CLN.MUC; GI.RECT.MUC.29; GI.RECT.MUC.31; GI.ESO; GI.STMC.GAST; PLCNT.FET; LIV.ADLT; LNG;	FAT.ADIP.NUC; MUS.SKLT.M; HRT.FET; GI.CLN.SM.MUS; GI.STMC.MUS; GI.CLN.MUC; GI.RECT.MUC.29; GI.RECT.MUC.31; GI.STMC.MUC; GI.DUO.MUC; LNG.FET; LIV.ADLT	BLD.GM12878; CRVX.HELAS3.CNCR; LIV.HEPG2.CNCR; BRST.HMEC; MUS.HSMMT; BLD.CD14.MONO; LNG.NHLF<br><br>**H3K27ac**<br>BLD.DND41.CNCR; BLD.CD14.MONO; BONE.OSTEO	**H3K9ac**<br>BLD.DND41.CNCR; BLD.CD14.MONO	ENCODE
rs72797306	14_EnhW2<br>(LNG.IMR90; ESC.H9; ESC.I3; ESC.HUES6; ESC.HUES48; ESC.HUES64; ESC.H1; ESC.4STAR; IPSC.20B; IPSC.18; IPSC.15b;	**14_EnhA2**<br>(LNG.A549.ETOH002.CNCR;	LNG.IMR90; ESC.WA7; ESC.H9; ESC.I3; ESC.HUES6; ESC.HUES48; ESC.HUES64; ESC.H1; ESC.4STAR; IPSC.20B; IPSC.18; IPSC.15b;	ESC.HUES6; IPSC.20B; IPSC.18; BLD.PER.MON	LNG.IMR90; ESC.HUES64; ESC.H1; IPSC.20B; IPSC.DF.19.11; ESDR.H1.BMP4.TROP;	LNG.IMR90; ESC.H9; ESC.I3; ESC.HUES6; ESC.H1;	**H3K4me1**<br>LNG.A549.ETOH002.CNCR;	**H3K4me3**<br>LNG.A549.ETOH0	ROADMAP<br><br>ENCODE

	IPSC.DF.19.11; ESDR.CD56.MESO; ESDR.CD56.ECTO; FAT.ADIP.DR.MSC; FAT.MSC.DR.ADIP; SKIN.PEN.FRSK.MEL.01; SKIN.PEN.FRSK.MEL.03; BRN.GANGEM.DR.NRSPHR; BRN.SUB.NIG; BRN.ANT.CAUD; BRN.CING.GYR; BRN.ANG.GYR; BRN.GRM.MTRX; BRN.FET.F; BRN.FET.M; MUS.PSOAS; MUS.SKLT.F; HRT.VENT.L; HRT.VNT.R; PLCNT.AMN; KID.FET; OVRY; PANC.ISLT)<br><br>17_EnhW2<br>(ESC.WA7; PSC.DF.6.9)<br><br>15_EnhAF<br>(ESDR.H1.NEUR.PROG; ESDR.CD184.ENDO; HRT.FET; VAS.AOR; GI.STMC.GAST; PANC)<br><br>16_EnhW1<br><br>(ESDR.H9.NEUR.PROG; ESDR.H9.NEUR; ESDR.H1.BMP4.MESO)<br><br>13_EnhA1<br>(ESDR.H1.BMP4.TROP; ESDR.H1.MSC; STRM.CHON.MRW.DR.MSC; SKIN.PEN.FRSK.FIB.01; SKIN.PEN.FRSK.FIB.02; BRST.HMEC.35; BRN.CRTX.DR.NRSPHR; BRN.HIPP.MID; BRN.INF.TMP; BRN.DL.PRFRNTL.CRTX; MUS.TRNK.FET; HRT.ATR.R; GI.STMC.FET; GI.S.INT; GI.CLN.SIG; GI.ESO; LNG)<br><br>3_PromD1<br><br>(BLD.PER.MONUC.PC; BLD.CD3.PPC; BLD.CD4.CD25I.CD127.TMEMPC; BLD.CD4.CD25.CD127M.TREGPC; BLD.CD4.CD25M.TPC; BLD.CD4.CD25M.CD45RA.NPC; BLD.CD4.CD25M.IL17M.PL.TPC; BLD.CD4.CD25M.IL17P.PL.TPC;	LIV.HEPG2.CNCR; BRN.NHA; SKIN.NHDFAD; LNG.NHLF)<br><br>**3_PromD1**<br>(BLD.DND41.CNCR; BLD.GM12878; BLD.CD14.MONO)<br><br>**15_EnhAF**<br>(CRVX.HELAS3.CNCR)<br><br>**2_PromU**<br>(BRST.HMEC; MUS.HSMM; MUS.HSMMT; SKIN.NHEK; BONE.OSTEO)<br><br>**13_EnhA1**<br>(VAS.HUVEC)<br><br>**15_EnhAF**<br>(BLD.K562.CNCR)	PSC.DF.6.9; IPSC.DF.19.11; ESDR.H1.NEUR.PROG; ESDR.H9.NEUR.PROG; ESDR.CD56.MESO; ESDR.CD56.ECTO; ESDR.CD184.ENDO; ESDR.H1.BMP4.MESO; ESDR.H1.BMP4.TROP; ESDR.H1.MSC; BLD.PER.MONUC.PC; BLD.CD3.PPC; BLD.CD4.CD25I.CD127.TMEMPC; BLD.CD3.CPC; BLD.CD4.CD25.CD127M.TREGPC; BLD.CD4.CD25M.TPC; BLD.CD4.CD25M.CD45RA.NPC; BLD.CD4.CD25M.IL17M.PL.TPC; BLD.CD4.CD25M.IL17P.PL.TPC; BLD.CD4.CD25M.CD45RO.MPC; BLD.CD4.MPC; BLD.CD8.MPC; BLD.CD4.NPC; BLD.CD8.NPC; BLD.CD14.PC; BLD.CD19.CPC; BLD.CD34.PC; BLD.MOB.CD34.PC.M; BLD.MOB.CD34.PC.F; BLD.CD34.CC; BLD.CD19.PPC; BLD.CD56.PC; BLD.CD15.PC; STRM.MRW.MSC; STRM.CHON.MRW.DR.MSC; FAT.ADIP.DR.MSC; FAT.MSC.DR.ADIP; MUS.SAT; SKIN.PEN.FRSK.FIB.01; SKIN.PEN.FRSK.FIB.02; SKIN.PEN.FRSK.MEL.01; SKIN.PEN.FRSK.MEL.03; SKIN.PEN.FRSK.KER.02; SKIN.PEN.FRSK.KER.03; BRST.HMEC.35; BRST.MYO; BRN.GANGEM.DR.NRSPHR; BRN.CRTX.DR.NRSPHR; THYM; THYM.FET; BRN.HIPP.MID; BRN.SUB.NIG; BRN.ANT.CAUD; BRN.CING.GYR; BRN.INF.TMP; BRN.ANG.GYR; BRN.DL.PRFRNTL.CRTX; BRN.FET.F; BRN.FET.M; FAT.ADIP.NUC; MUS.PSOAS;	UC.PC; BLD.CD3.PPC; BLD.CD4.CD25I.CD127.TMEMPC; BLD.CD3.CPC; BLD.CD4.CD25.CD127M.TREGPC; BLD.CD4.CD25M.TPC; BLD.CD4.CD25M.CD45RA.NPC; BLD.CD4.CD25M.IL17M.PL.TPC; BLD.CD4.CD25M.IL17P.PL.TPC; BLD.CD4.CD25M.CD45RO.MPC; BLD.CD4.MPC; BLD.CD8.MPC; BLD.CD4.NPC; BLD.CD8.NPC; BLD.CD14.PC; BLD.CD19.CPC; BLD.CD34.PC; BLD.MOB.CD34.PC.M; BLD.MOB.CD34.PC.F; BLD.CD34.CC; BLD.CD19.PPC; BLD.CD15.PC; STRM.MRW.MSC; STRM.CHON.MRW.DR.MSC; MUS.SAT; SKIN.PEN.FRSK.FIB.01; SKIN.PEN.FRS	ESDR.H1.MSC; BLD.PER.MONUC.PC; BLD.CD3.PPC; BLD.CD4.CD25I.CD127.TMEMPC; BLD.CD4.CD25.CD127M.TREGPC; BLD.CD4.CD25M.TPC; BLD.CD4.CD25M.CD45RA.NPC; BLD.CD4.CD25M.IL17M.PL.TPC; BLD.CD4.CD25M.IL17P.PL.TPC; BLD.CD4.CD25M.CD45RO.MPC; BLD.CD4.MPC; BLD.CD8.MPC; BLD.CD4.NPC; BLD.CD8.NPC; BLD.CD14.PC; BLD.MOB.CD34.PC.F; BLD.CD19.PPC; BLD.CD56.PC; STRM.MRW.MSC; SKIN.PEN.FRSK.FIB.01; SKIN.PEN.FRSK.FIB.02; SKIN.PEN.FRSK.MEL.01; SKIN.PEN.FRSK.KER.03; THYM; THYM.FET; BRN.HIPP.MID; BRN.SUB.NIG; BRN.ANT.CAUD; BRN.CING.GYR; BRN.INF.TMP; BRN.ANG.GYR; BRN.DL.PRFRNTL.CRTX; FAT.ADIP.NUC; MUS.PSOAS; MUS.SKLT.F; MUS.TRNK.FET; MUS.LEG.FET; HRT.ATR.R; HRT.VENT.L; HRT.VNT.R; GI.DUO.SM.MUS; GI.CLN.SM.MUS; GI.RECT.SM.MUS; GI.STMC.MUS; GI.STMC.FET; GI.S.INT.FET; GI.L.INT.FET; GI.S.INT; GI.CLN.SIG; GI.CLN.MUC; GI.RECT.MUC.29;	IPSC.20B; IPSC.15b; ESDR.H1.NEUR.PROG; ESDR.H1.MSC; BLD.PER.MONUC.PC; BLD.CD4.NPC; BLD.CD8.NPC; STRM.MRW.MSC; STRM.CHON.MRW.DR.MSC; MUS.SAT; BRST.MYO; BRN.SUB.NIG; BRN.ANT.CAUD; BRN.CING.GYR; BRN.INF.TMP; BRN.ANG.GYR; BRN.DL.PRFRNTL.CRTX; FAT.ADIP.NUC; MUS.SKLT.F; MUS.SKLT.M; GI.CLN.SM.MUS; GI.RECT.SM.MUS; GI.STMC.MUS; GI.CLN.MUC; GI.RECT.MUC.29; GI.RECT.MUC.31; GI.STMC.MUC; GI.DUO.MUC; LNG.FET; PANC.ISLT; LIV.ADLT.	BLD.DND41.CNCR; BLD.GM12878; CRVX.HELAS3.CNCR; LIV.HEPG2.CNCR; BRST.HMEC; MUS.HSMM; MUS.HSMMT; VAS.HUVEC; BLD.K562.CNCR; BLD.CD14.MONO; BRN.NHA; SKIN.NHDFAD; SKIN.NHEK; LNG.NHLF; BONE.OSTEO<br><br>**H3K27ac**<br>BLD.DND41.CNCR; BLD.GM12878; CRVX.HELAS3.CNCR; BRST.HMEC; MUS.HSMM; MUS.HSMMT; BLD.CD14.MONO;	02.CNCR; BLD.DND41.CNCR; BLD.GM12878; BRST.HMEC; MUS.HSMM; MUS.HSMMT; BLD.CD14.MONO; SKIN.NHEK; BONE.OSTEO<br><br>**H3K9ac**<br>LNG.A549.ETOH02.CNCR; BLD.DND41.CNCR; BLD.GM12878; LIV.HEPG2.CNCR; BRST.HMEC; MUS.HSMM; MUS.HSMMT; VAS.HUVEC; BLD.CD14.MONO; SKIN.NHEK;	IPSC.DF.6.9; BLD.CD3.PPC; SKIN.PEN.FRSK.MEL.01; HYM.FET; GI.STMC.FET; GI.L.INT.FET; MUS.HSMMT

	BLD.CD4.CD25M.CD45RO.MPC; BLD.CD4.MPC; BLD.CD8.MPC; BLD.CD4.NPC; BLD.CD8.NPC; BLD.CD14.PC; BLD.CD19.CPC; BLD.CD34.PC; BLD.MOB.CD34.PC.M; BLD.MOB.CD34.PC.F; BLD.CD34.CC; BLD.CD19.PPC; BLD.CD56.PC; BLD.CD15.PC; THYM; THYM.FET; FAT.ADIP.NUC; GI.DUO.SM.MUS; GI.S.INT.FET; GI.CLN.MUC; GI.RECT.MUC.29; GI.DUO.MUC; PLCNT.FET; SPLN)<br><br>2_PromU (BLD.CD3.CPC; STRM.MRW.MSC; MUS.SAT; SKIN.PEN.FRSK.KER.02; SKIN.PEN.FRSK.KER.03; BRST.MYO; MUS.SKLT.M; MUS.LEG.FET; GI.CLN.SM.MUS; GI.RECT.SM.MUS; GI.STMC.MUS; GI.L.INT.FET; GI.RECT.MUC.31; GI.STMC.MUC; LNG.FET; ADRL.GLND.FET; LIV.ADLT)		MUS.SKLT.F; MUS.SKLT.M; MUS.TRNK.FET; MUS.LEG.FET; HRT.FET; HRT.ATR.R; HRT.VENT.L; HRT.VNT.R; GI.DUO.SM.MUS; GI.CLN.SM.MUS; GI.RECT.SM.MUS; GI.STMC.MUS; GI.STMC.FET; GI.S.INT.FET; GI.L.INT.FET; GI.S.INT; GI.CLN.SIG; GI.CLN.MUC; GI.RECT.MUC.29; GI.RECT.MUC.31; GI.STMC.MUC; GI.DUO.MUC; GI.ESO; GI.STMC.GAST; PLCNT.AMN; KID.FET; LNG.FET; OVRY; PANC.ISLT; ADRL.GLND.FET; PLCNT.FET; LIV.ADLT; PANC; LNG; SPLN	K.FIB.02; SKIN.PEN.FRSK.KER.02; SKIN.PEN.FRSK.KER.03; BRST.MYO; THYM; THYM.FET; BRN.HIPP.MID; BRN.ANT.CAUD; BRN.DL.PRFRNTL.CRTX; FAT.ADIP.NUC; MUS.SKLT.F; MUS.SKLT.M; MUS.LEG.FET; GI.DUO.SM.MUS; GI.CLN.SM.MUS; GI.RECT.SM.MUS; GI.STMC.MUS; GI.CLN.SIG; GI.CLN.MUC; GI.RECT.MUC.29; GI.RECT.MUC.31; GI.STMC.MUC; GI.DUO.MUC; GI.ESO; PLCNT.AMN; LIV.ADLT; SPLN	GI.RECT.MUC.31; GI.ESO; GI.STMC.GAST; OVRY; PANC.ISLT; ADRL.GLND.FET; PLCNT.FET; LIV.ADLT; PANC; LNG; SPLN		SKIN.NHDFAD; SKIN.NHEK; LNG.NHLF; BONE.OSTEO	LNG.NHLF	
rs2248116	**17_EnhW2** (ESDR.H1.MSC; BLD.PER.MONUC.PC; BLD.CD4.CD25M.IL17P.PL.TPC; BLD.CD8.MPC; BLD.CD4.NPC; BLD.CD8.NPC; BLD.CD34.PC; STRM.MRW.MSC; STRM.CHON.MRW.DR.MSC; FAT.MSC.DR.ADIP; MUS.SAT; SKIN.PEN.FRSK.FIB.01;	**17_EnhW2** (BLD.DND41.CNCR; BRST.HMEC; MUS.HSMM; MUS.HSMMT;	LNG.IMR90; ESDR.CD56.MESO; BLD.PER.MONUC.PC; BLD.CD3.PPC; BLD.CD4.CD25I.CD127.TMEMPC; BLD.CD3.CPC; BLD.CD4.CD25.CD127M.TREGPC; BLD.CD4.CD25M.TPC; BLD.CD4.CD25M.CD45RA.NPC; BLD.CD4.CD25M.IL17M.PL.TPC;	BLD.CD4.CD25M.TPC; BLD.CD8.MPC; BLD.CD4.NPC; BLD.CD19.CPC; BLD.MOB.CD34.PC.M; BLD.CD19.PPC	ESDR.H1.MSC; BLD.PER.MONUC.PC; BLD.CD3.PPC; BLD.CD4.CD25I.CD127.TMEMPC; BLD.CD4.CD25.CD127M.TREGPC; BLD.CD4.CD25M.TPC; BLD.CD4.CD25M.CD45RA.NPC;	FAT.MSC.DR.ADIP; MUS.SAT; GI.RECT.MUC.29; GI.DUO.MUC	**H3K4me1** BLD.DND41.CNCR; BLD.GM12878; CRVX.HELAS3.CNCR; LIV.HEPG2	**BLD.DND41.CNCR; MUS.HSMMT**	

	SKIN.PEN.FRSK.FIB.02; BRST.HMEC.35; FAT.ADIP.NUC; GI.S.INT; GI.CLN.SIG; GI.CLN.MUC; GI.RECT.MUC.29; GI.RECT.MUC.31; GI.STMC.MUC; GI.DUO.MUC; SPLN)<br><br>**10_TxEnh5**<br>(BLD.CD3.PPC; BLD.CD4.CD25M.TPC; BLD.CD4.MPC; BLD.MOB.CD34.PC.F; BLD.CD19.PPC; BLD.CD56.PC; THYM.FET)<br><br>**12_TxEnhW**<br>(BLD.CD4.CD25I.CD127.TMEMPC; BLD.CD3.CPC; BLD.CD4.CD25.CD127M.TREGPC; BLD.CD4.CD25M.CD45RA.NPC; BLD.CD4.CD25M.IL17M.PL.TPC; BLD.CD4.CD25M.CD45RO.MPC; THYM)<br><br>**15_EnhAF**<br>(BLD.CD14.PC; BLD.CD19.CPC; BLD.MOB.CD34.PC.M; BLD.CD34.CC)<br><br>**9_TxReg**<br>(BLD.CD15.PC)	BLD.K562.CNCR; BRN.NHA; SKIN.NHDFAD; LNG.NHLF)<br><br>**12_TxEnhW**<br>(BLD.GM12878)<br><br>**10_TxEnh5**<br>(BLD.CD14.MONO)<br><br>**15_EnhAF**<br>(BONE.OSTEO)	BLD.CD4.CD25M.IL17P.PL.TPC; BLD.CD4.CD25M.CD45RO.MPC; BLD.CD4.MPC; BLD.CD8.MPC; BLD.CD4.NPC; BLD.CD8.NPC; BLD.CD14.PC; BLD.CD19.CPC; BLD.CD34.PC; BLD.MOB.CD34.PC.M; BLD.MOB.CD34.PC.F; BLD.CD34.CC; BLD.CD19.PPC; BLD.CD56.PC; BLD.CD15.PC; STRM.MRW.MSC; STRM.CHON.MRW.DR.MSC; FAT.ADIP.DR.MSC; FAT.MSC.DR.ADIP; MUS.SAT; SKIN.PEN.FRSK.FIB.01; SKIN.PEN.FRSK.FIB.02; SKIN.PEN.FRSK.KER.02; SKIN.PEN.FRSK.KER.03; BRST.HMEC.35; BRST.MYO; THYM.FET; BRN.DL.PRFRNTL.CRTX; FAT.ADIP.NUC; GI.DUO.SM.MUS; GI.CLN.SM.MUS; GI.STMC.MUS; GI.STMC.FET; GI.S.INT.FET; GI.L.INT.FET; GI.S.INT; GI.CLN.MUC; GI.RECT.MUC.29; GI.RECT.MUC.31; GI.STMC.MUC; GI.DUO.MUC; PLCNT.FET; LIV.ADLT	; BLD.CD15.PC; STRM.MRW.MSC; GI.DUO.SM.MUS; GI.CLN.SIG; GI.CLN.MUC; GI.DUO.MUC	BLD.CD4.CD25M.IL17M.PL.TPC; BLD.CD4.CD25M.IL17P.PL.TPC; BLD.CD4.CD25M.CD45RO.MPC; BLD.CD4.MPC; BLD.CD8.MPC; BLD.CD8.NPC; BLD.MOB.CD34.PC.F; BLD.CD19.PPC; BLD.CD56.PC; STRM.MRW.MSC; THYM; THYM.FET; FAT.ADIP.NUC; MUS.SKLT.F; MUS.TRNK.FET; GI.DUO.SM.MUS; GI.STMC.MUS; GI.L.INT.FET; GI.S.INT; GI.CLN.SIG; GI.CLN.MUC; GI.RECT.MUC.29; GI.ESO; ADRL.GLND.FET; PLCNT.FET; LIV.ADLT; PANC; SPLN		.CNCR; BRST.HMEC; MUS.HSMM; MUS.HSMMT; VAS.HUVEC; BLD.CD14.MONO; BRN.NHA; SKIN.NHDFAD; SKIN.NHEK; LNG.NHLF; BONE.OSTEO<br><br>**H3K27ac**<br>BLD.DND41.CNCR; BLD.GM12878; MUS.HSMM; MUS.HSMMT; BLD.CD14.MONO; LNG.NHLF; BONE.OSTEO		
rs11741255	**15_EnhAF**<br>(LNG.IMR90; IPSC.20B; IPSC.18; ESDR.H1.NEUR.PROG; ESDR.H9.NEUR; ESDR.H1.MSC; BLD.PER.MONUC.PC; BLD.CD3.CPC; BLD.CD4.NPC; BLD.CD14.PC; BLD.CD19.CPC; BLD.CD34.PC; BLD.MOB.CD34.PC.M; BLD.MOB.CD34.PC.F; BLD.CD19.PPC; STRM.MRW.MSC; STRM.CHON.MRW.DR.MSC; FAT.ADIP.DR.MSC;	**17_EnhW2**<br>(LNG.A549.ETOH002.CNCR; CRVX.HELAS3.CNCR; MUS.HSMM; MUS.HSMMT; VAS.HUVEC	LNG.IMR90; ESC.WA7; ESC.H9; ESC.I3; ESC.HUES6; ESC.HUES48; ESC.HUES64; ESC.H1; ESC.4STAR; IPSC.20B; IPSC.18; IPSC.15b; IPSC.DF.19.11; ESDR.H1.NEUR.PROG; ESDR.H9.NEUR.PROG; ESDR.H9.NEUR; ESDR.CD56.MESO; ESDR.CD56.ECTO; ESDR.CD184.ENDO;	BLD.CD4.CD25.CD127M.TREGPC; FAT.MSC.DR.ADIP; SKIN.PEN.FRSK.FIB.02; GI.DUO.SM.MUS; GI.CLN.MUC; GI.RECT.MUC.	ESC.H1; IPSC.20B; IPSC.DF.19.11; ESDR.H1.BMP4.TROP; BLD.PER.MONUC.PC; BLD.CD3.PPC; BLD.CD4.CD25I.CD127.TMEMPC; BLD.CD4.CD25.CD127M.TREGPC; BLD.CD4.CD25M.TPC; BLD.CD4.CD25M.CD45RA.NPC;	ESC.HUES6; ESC.H1; IPSC.20B; IPSC.15b; BLD.PER.MONUC.PC; BLD.CD4.NPC; BLD.CD8.NPC; STRM.MRW.MSC; STRM.CHON.M	**H3K4me1**<br>LNG.A549.ETOH002.CNCR; BLD.DND41.CNCR; LIV.HEPG2.CNCR; BRST.HMEC; BLD.CD14.	**H3K4me3**<br>LIV.HEPG2.CNCR<br><br>**H3K9ac**<br>BLD.DND41.CNCR	ROADMAP ESDR.H1.BMP4.MESO, ESDR.H1.MSC, BLD.CD3.PPC, BLD.MOB.CD34.PC.F, BLD.CD19.P

	SKIN.PEN.FRSK.FIB.01; SKIN.PEN.FRSK.MEL.01; SKIN.PEN.FRSK.MEL.03; SKIN.PEN.FRSK.KER.03; BRST.MYO; BRN.GANGEM.DR.NRSPHR; BRN.CRTX.DR.NRSPHR; THYM; BRN.SUB.NIG; BRN.ANT.CAUD; BRN.CING.GYR; BRN.INF.TMP; BRN.ANG.GYR; BRN.DL.PRFRNTL.CRTX; BRN.GRM.MTRX; BRN.FET.F; BRN.FET.M; MUS.PSOAS; HRT.ATR.R; VAS.AOR; GI.ESO; GI.STMC.GAST; PLCNT.AMN; KID.FET; LNG.FET; OVRY; PANC.ISLT; PANC; LNG; SPLN)<br><br>**17_EnhW2**<br>(ESC.WA7; ESC.H9; ESC.I3; ESC.HUES48; ESC.HUES64; ESC.H1; ESC.4STAR; IPSC.15b; PSC.DF.6.9; ESDR.H9.NEUR.PROG; ESDR.CD56.MESO; ESDR.CD56.ECTO; ESDR.CD184.ENDO; ESDR.H1.BMP4.MESO; MUS.SAT; SKIN.PEN.FRSK.KER.02; BRST.HMEC.35)<br><br>**14_EnhA2**<br>(ESC.HUES6; IPSC.DF.19.11; ESDR.H1.BMP4.TROP)<br><br>**9_TxReg**<br>(BLD.CD3.PPC; BLD.CD4.CD25.CD127M.TREGPC; BLD.CD4.CD25M.TPC; BLD.CD4.CD25M.IL17M.PL.TPC; BLD.CD4.CD25M.CD45RO.MPC; BLD.CD4.MPC; BLD.CD56.PC; THYM.FET; GI.S.INT.FET)<br><br>**13_EnhA1**<br>(BLD.CD4.CD25I.CD127.TMEMPC; BLD.CD4.CD25M.IL17P.PL.TPC; BLD.CD8.NPC; BLD.CD34.CC; BLD.CD15.PC; FAT.MSC.DR.ADIP)<br><br>**10_TxEnh5**	; BRN.NHA; SKIN.NHDFAD; LNG.NHLF)<br><br>**15_EnhAF**<br>(BLD.DND41.CNCR; BLD.GM12878; BRST.HMEC; BLD.K562.CNCR; BLD.CD14.MONO; BONE.OSTEO)<br><br>**13_EnhA1**<br>(LIV.HEPG2.CNCR)<br><br>**14_EnhA2**<br>(SKIN.NHEK)	ESDR.H1.BMP4.TROP; ESDR.H1.MSC; BLD.PER.MONUC.PC; BLD.CD4.CD25I.CD127.TMEMPC; BLD.CD3.CPC; BLD.CD4.CD25.CD127M.TREGPC; BLD.CD4.CD25M.TPC; BLD.CD4.CD25M.CD45RA.NPC; BLD.CD4.CD25M.IL17M.PL.TPC; BLD.CD4.CD25M.IL17P.PL.TPC; BLD.CD4.CD25M.CD45RO.MPC; BLD.CD4.MPC; BLD.CD8.MPC; BLD.CD4.NPC; BLD.CD8.NPC; BLD.CD34.PC; BLD.MOB.CD34.PC.M; BLD.MOB.CD34.PC.F; BLD.CD34.CC; BLD.CD19.PPC; BLD.CD56.PC; BLD.CD15.PC; STRM.MRW.MSC; STRM.CHON.MRW.DR.MSC; FAT.ADIP.DR.MSC; FAT.MSC.DR.ADIP; SKIN.PEN.FRSK.FIB.01; SKIN.PEN.FRSK.FIB.02; SKIN.PEN.FRSK.KER.02; SKIN.PEN.FRSK.KER.03; BRST.MYO; THYM; THYM.FET; BRN.HIPP.MID; BRN.SUB.NIG; BRN.ANT.CAUD; BRN.CING.GYR; BRN.INF.TMP; BRN.ANG.GYR; BRN.DL.PRFRNTL.CRTX; FAT.ADIP.NUC; MUS.PSOAS; MUS.SKLT.F; MUS.SKLT.M; MUS.TRNK.FET; MUS.LEG.FET; HRT.FET; HRT.ATR.R; HRT.VENT.L; HRT.VNT.R; GI.DUO.SM.MUS; GI.CLN.SM.MUS; GI.RECT.SM.MUS; GI.STMC.MUS; GI.STMC.FET; GI.S.INT.FET; GI.L.INT.FET; GI.S.INT; GI.CLN.SIG; GI.CLN.MUC; GI.RECT.MUC.29; GI.RECT.MUC.31; GI.STMC.MUC; GI.DUO.MUC; GI.ESO; GI.STMC.GAST; PLCNT.AMN; LNG.FET; OVRY; ADRL.GLND.FET; PLCNT.FET; LIV.ADLT; PANC; LNG; SPLN	31; GI.STMC.MUC; GI.DUO.MUC; SPLN	BLD.CD4.CD25M.IL17M.PL.TPC; BLD.CD4.CD25M.IL17P.PL.TPC; BLD.CD4.CD25M.CD45RO.MPC; BLD.CD4.MPC; BLD.CD8.MPC; BLD.CD4.NPC; BLD.CD8.NPC; BLD.CD14.PC; BLD.MOB.CD34.PC.F; BLD.CD19.PPC; BLD.CD56.PC; SKIN.PEN.FRSK.FIB.01; SKIN.PEN.FRSK.FIB.02; SKIN.PEN.FRSK.MEL.01; SKIN.PEN.FRSK.MEL.03; THYM; THYM.FET; BRN.HIPP.MID; BRN.SUB.NIG; BRN.ANT.CAUD; BRN.CING.GYR; BRN.INF.TMP; BRN.ANG.GYR; BRN.DL.PRFRNTL.CRTX; FAT.ADIP.NUC; MUS.PSOAS; MUS.SKLT.F; MUS.TRNK.FET; MUS.LEG.FET; HRT.ATR.R; HRT.VENT.L; HRT.VNT.R; GI.DUO.SM.MUS; GI.CLN.SM.MUS; GI.RECT.SM.MUS; GI.STMC.MUS; GI.STMC.FET; GI.S.INT.FET; GI.L.INT.FET; GI.S.INT; GI.CLN.SIG; GI.CLN.MUC; GI.RECT.MUC.29; GI.RECT.MUC.31; GI.ESO; GI.STMC.GAST; PLCNT.AMN; OVRY; ADRL.GLND.FET; PLCNT.FET; LIV.ADLT; PANC; LNG; SPLN	RW.DR.MSC; FAT.ADIP.DR.MSC; FAT.MSC.DR.ADIP; BRN.SUB.NIG; BRN.ANT.CAUD; BRN.CING.GYR; BRN.INF.TMP; BRN.ANG.GYR; BRN.DL.PRFRNTL.CRTX; FAT.ADIP.NUC; MUS.SKLT.F; MUS.SKLT.M; HRT.FET; GI.DUO.SM.MUS; GI.CLN.SM.MUS; GI.RECT.SM.MUS; GI.CLN.MUC; GI.RECT.MUC.29; GI.RECT.MUC.31; GI.STMC.MUC; GI.DUO.MUC; LNG.FET; LIV.ADLT	MONO; SKIN.NHDFAD; SKIN.NHEK; LNG.NHLF; BONE.OSTEO<br><br>**H3K27ac**<br>BLD.DND41.CNCR; BLD.GM12878; LIV.HEPG2.CNCR; BLD.CD14.MONO	; LIV.HEPG2.CNCR; SKIN.NHEK	PC, GI.L.INT.FET<br><br>ENCODE BLD.CD14.MONO

	(BLD.CD4.CD25M.CD45RA.NPC; BLD.CD8.MPC)<br><br>**14_EnhA2**<br>(SKIN.PEN.FRSK.FIB.02; MUS.LEG.FET; HRT.FET)<br><br>**13_EnhA1**<br>(BRN.HIPP.MID; FAT.ADIP.NUC; MUS.SKLT.F; MUS.SKLT.M; MUS.TRNK.FET; HRT.VENT.L; HRT.VNT.R; GI.DUO.SM.MUS; GI.CLN.SM.MUS; GI.RECT.SM.MUS; GI.STMC.MUS; GI.STMC.FET; GI.L.INT.FET; GI.S.INT; GI.CLN.SIG; GI.CLN.MUC; GI.RECT.MUC.29; GI.RECT.MUC.31; GI.STMC.MUC; GI.DUO.MUC; ADRL.GLND.FET; PLCNT.FET; LIV.ADLT)								
**rs17024629**	**14_EnhA2**<br>(FAT.ADIP.DR.MSC); 17_EnhW2 (STRM.CHON.MRW.DR.MSC; FAT.MSC.DR.ADIP; SKIN.PEN.FRSK.FIB.02; BRN.ANT.CAUD; FAT.ADIP.NUC; GI.DUO.SM.MUS;GI.STMC.MUC; GI.DUO.MUC)<br><br>**18_EnhAc**<br>(SKIN.PEN.FRSK.FIB.01)	**13_EnhA2**<br>(CRVX.HELAS3.CNCR); 14_EnhA2 (LNG.A549.ETOH002.CNCR; LIV.HEPG2.CNCR); 17EnhW2 (SKIN.NHDFAD; BONE.OSTEO)	LNG.IMR90; ESC.WA7; ESC.I3; ESC.HUES6; ESC.4STAR; IPSC.20B; IPSC.18; IPSC.15b; IPSC.DF.19.11; ESDR.CD56.ECTO; ESDR.CD184.ENDO; BLD.CD3.PPC; BLD.CD4.CD25I.CD127.TMEMPC; BLD.MOB.CD34.PC.F; STRM.MRW.MSC; FAT.ADIP.DR.MSC; FAT.MSC.DR.ADIP; MUS.SAT; SKIN.PEN.FRSK.FIB.01; SKIN.PEN.FRSK.FIB.02; BRN.HIPP.MID; BRN.ANT.CAUD; BRN.ANG.GYR; BRN.GRM.MTRX; FAT.ADIP.NUC; MUS.TRNK.FET; MUS.LEG.FET; HRT.ATR.R; HRT.VENT.L; HRT.VNT.R; GI.DUO.SM.MUS; GI.CLN.SM.MUS; GI.STMC.MUS; GI.STMC.FET; GI.S.INT.FET; GI.L.INT.FET; GI.S.INT; GI.CLN.MUC; GI.RECT.MUC.29; GI.STMC.MUC; GI.DUO.MUC; PLCNT.AMN	ESC.WA7; SKIN.PEN.FRSK.FIB.02; GI.STMC.MUS;	LNG.IMR90; ESC.HUES48; ESC.H1; IPSC.20B; IPSC.DF.19.11; ESDR.CD56.MESO; ESDR.H1.BMP4.MESO; ESDR.H1.MSC; BLD.CD4.CD25I.CD127.TMEMPC; SKIN.PEN.FRSK.FIB.02; BRN.ANT.CAUD; BRN.CING.GYR; BRN.INF.TMP; FAT.ADIP.NUC; HRT.ATR.R; GI.DUO.SM.MUS; GI.RECT.SM.MUS; GI.STMC.MUS; GI.S.INT; GI.CLN.SIG; LNG	ESC.HUES6; IPSC.15b; BLD.PER.MONUC.PC; BRN.CING.GYR; BRN.ANG.GYR; MUS.SKLT.M; GI.STMC.MUS; GI.STMC.MUC; GI.DUO.MUC	**H3K4me1**<br>(LNG.A549.ETOH002.CNCR; CRVX.HELAS3.CNCR; LIV.HEPG2.CNCR; MUS.HSMMT; SKIN.NHDFAD; LNG.NHLF; BONE.OSTEO)<br><br>**H3K27ac**<br>LNG.A549.ETOH002.CNCR; CRVX.HELAS3.CNCR; LIV.HEPG2.CNCR	**H3K4me3**<br>LNG.A549.ETOH002.CNCR; CRVX.HELAS3.CNCR; LIV.HEPG2.CNCR<br><br>**H3K9ac**<br>LNG.A549.ETOH002.CNCR; CRVX.HELAS3.CNCR; LIV.HEPG2.CNCR;	SKIN.PEN.FRSK.FIB.01

rs538388	**11_TxEnh3** (ESDR.H1.NEUR.PROG; SKIN.PEN.FRSK.FIB.01; SKIN.PEN.FRSK.MEL.01; SKIN.PEN.FRSK.MEL.03; SKIN.PEN.FRSK.KER.02; BRN.GANGEM.DR.NRSPHR; BRN.CRTX.DR.NRSPHR; BRN.HIPP.MID; BRN.SUB.NIG; BRN.ANT.CAUD; BRN.CING.GYR; BRN.ANG.GYR; BRN.DL.PRFRNTL.CRTX; BRN.GRM.MTRX; BRN.FET.F; BRN.FET.M; MUS.SKLT.F; MUS.SKLT.M; MUS.LEG.FET; GI.DUO.SM.MUS; GI.CLN.SM.MUS; GI.STMC.MUS; GI.STMC.FET; GI.S.INT.FET; GI.L.INT.FET; PLCNT.AMN; LNG.FET; ADRL.GLND.FET; PLCNT.FET; SPLN)<br><br>**9_TxReg** (SKIN.PEN.FRSK.FIB.02; BRN.INF.TMP; MUS.TRNK.FET)	**11_TxEnh3** (MUS.HSMM; MUS.HSMMT)	ESC.WA7; IPSC.DF.19.11; ESDR.H1.NEUR.PROG; ESDR.H1.BMP4.TROP; ESDR.H1.MSC; BLD.CD3.PPC; BLD.CD4.CD25I.CD127.TMEMPC; BLD.CD3.CPC; BLD.CD4.CD25. BLD.CD34.CC; SKIN.PEN.FRSK.FIB.01; SKIN.PEN.FRSK.FIB.02; SKIN.PEN.FRSK.MEL.01; BRST.MYO; BRN.GANGEM.DR.NRSPHR; BRN.CRTX.DR.NRSPHR; BRN.HIPP.MID; BRN.SUB.NIG; BRN.ANT.CAUD; BRN.CING.GYR; BRN.INF.TMP; BRN.ANG.GYR; BRN.DL.PRFRNTL.CRTX; BRN.GRM.MTRX; BRN.FET.M; MUS.SKLT.M; MUS.TRNK.FET; MUS.LEG.FET; HRT.ATR.R; HRT.VNT.R; GI.DUO.SM.MUS; GI.STMC.MUS; GI.STMC.FET; GI.S.INT.FET; GI.L.INT.FET; GI.CLN.MUC; GI.RECT.MUC.29; GI.RECT.MUC.31; GI.STMC.MUC; GI.DUO.MUC; GI.ESO; GI.STMC.GAST; ADRL.GLND.FET; PANC; LNG; SPLN	ESC.H9; IPSC.DF.19.11; ESDR.H1.NEUR.PROG; ESDR.H1.MSC; SKIN.PEN.FRSK.FIB.01; SKIN.PEN.FRSK.FIB.02; SKIN.PEN.FRSK.MEL.01; BRN.GANGEM.DR.NRSPHR; BRN.GRM.MTRX; BRN.FET.F; BRN.FET.M; FAT.ADIP.NUC; MUS.PSOAS; MUS.SKLT.F; MUS.SKLT.M; MUS.TRNK.FET; MUS.LEG.FET; HRT.FET; HRT.ATR.R; HRT.VENT.L; HRT.VNT.R; VAS.AOR; GI.DUO.SM.MUS; GI.CLN.SM.MUS; GI.RECT.SM.MUS; GI.STMC.MUS; GI.STMC.FET; GI.S.INT.FET; GI.L.INT.FET; GI.S.INT; GI.CLN.SIG; GI.CLN.MUC; GI.RECT.MUC.29; GI.RECT.MUC.31; GI.STMC.MUC;	ESC.H9; IPSC.20B; IPSC.DF.19.11; ESDR.H1.NEUR.PROG; ESDR.CD56.MESO; ESDR.CD56.ECTO; ESDR.CD184.ENDO; BLD.CD4.CD25I.CD127.TMEMPC; BLD.CD4.CD25M.CD45RA.NPC; BLD.CD4.CD25M.IL17M.PL.TPC; BLD.CD4.CD25M.IL17P.PL.TPC; BLD.CD4.CD25M.CD45RO.MPC; BLD.MOB.CD34.PC.F; SKIN.PEN.FRSK.FIB.01; SKIN.PEN.FRSK.FIB.02; SKIN.PEN.FRSK.MEL.01; BRN.HIPP.MID; BRN.SUB.NIG; BRN.ANT.CAUD; BRN.CING.GYR; BRN.INF.TMP; BRN.ANG.GYR; BRN.DL.PRFRNTL.CRTX; MUS.TRNK.FET; MUS.LEG.FET; GI.CLN.SM.MUS; GI.STMC.MUS; GI.STMC.FET; GI.S.INT.FET; GI.L.INT.FET; GI.S.INT; GI.ESO; ADRL.GLND.FET; LNG; SPLN	LNG.IMR90; ESC.HUES6; IPSC.15b; ESDR.H1.NEUR.PROG; SDR.H1.MSC; STRM.CHON.MRW.DR.MSC; BRN.SUB.NIG; BRN.ANT.CAUD; BRN.CING.GYR; BRN.INF.TMP; BRN.ANG.GYR; BRN.DL.PRFRNTL.CRTX; MUS.SKLT.M; GI.STMC.MUS; GI.RECT.MUC.31; GI.STMC.MUC; GI.DUO.MUC; PANC.ISLT;	**H3K4me1** LNG.A549.ETOH002.CNCR; MUS.HSMM; MUS.HSMMT;<br><br>**H3K27ac** MUS.HSMMT; BLD.CD14.MONO; BRN.NHA; BONE.OSTEO	**H3K4me3** LNG.A549.ETOH002.CNCR; BLD.DND41.CNCR; BLD.GM12878; CRVX.HELAS3.CNCR; LIV.HEPG2.CNCR; BRST.HMEC; MUS.HSMM; MUS.HSMMT; VAS.HUVEC; BLD.K562.CNCR; BLD.CD14.MONO; BRN.NHA; SKIN.NHDFAD; SKIN.NHEK; LNG.NHLF; BONE.OSTEO<br><br>**H3K9ac** CRVX.HELAS3.CNCR;	

				GI.DUO.MUC; GI.ESO; GI.STMC.GAST; PLCNT.AMN; KID.FET; LNG.FET; OVRY; PANC.ISLT; ADRL.GLND.FET; PLCNT.FET; LIV.ADLT; PANC; LNG; SPLN					
rs560674	**11_TxEnh3** (LNG.IMR90; ESDR.CD56.MESO; ESDR.H1.BMP4.TROP; SKIN.PEN.FRSK.MEL.01; BRST.HMEC.35)<br><br>**10_TxEnh5** (ESDR.H1.MSC; SKIN.PEN.FRSK.FIB.01; SKIN.PEN.FRSK.FIB.02; SKIN.PEN.FRSK.KER.03)	**11_TxEnh3** (LNG.A549.ETOH002.CNCR; VAS.HUVEC; SKIN.NHEK; LNG.NHLF; BONE.OSTEO)<br><br>**10_TxEnh5** (BRN.NHA)	LNG.IMR90; IPSC.15b; IPSC.DF.19.11; ESDR.H1.BMP4.TROP; ESDR.H1.MSC; BLD.CD3.PPC; SKIN.PEN.FRSK.FIB.01; SKIN.PEN.FRSK.FIB.02; SKIN.PEN.FRSK.MEL.01; SKIN.PEN.FRSK.KER.02; SKIN.PEN.FRSK.KER.03; BRST.HMEC.35; BRST.MYO; BRN.ANT.CAUD; BRN.DL.PRFRNTL.CRTX; MUS.TRNK.FET; HRT.VNT.R; GI.RECT.MUC.29; GI.STMC.GAST; PLCNT.AMN; PANC;	IPSC.DF.19.11; SKIN.PEN.FRSK.FIB.01; SKIN.PEN.FRSK.FIB.02; SKIN.PEN.FRSK.MEL.01; SPLN	ESC.HUES48; IPSC.20B; ESDR.CD184.ENDO; ESDR.H1.MSC; BLD.CD4.CD25I.CD127.TMEMPC; BLD.CD4.CD25M.CD45RO.MPC; BLD.MOB.CD34.PC.F; SKIN.PEN.FRSK.FIB.01; SKIN.PEN.FRSK.FIB.02; SKIN.PEN.FRSK.MEL.01; BRN.HIPP.MID; BRN.ANT.CAUD; BRN.CING.GYR; BRN.INF.TMP; BRN.ANG.GYR; BRN.DL.PRFRNTL.CRTX; HRT.VNT.R; GI.STMC.MUS; PANC.ISLT; LNG; SPLN	LNG.IMR90; ESC.HUES6; ESDR.H1.NEUR.PROG; STRM.CHON.MRW.DR.MSC; BRN.ANT.CAUD; BRN.CING.GYR; BRN.ANG.GYR; GI.STMC.MUS; PANC.ISLT;	**H3K4me1** BRST.HMEC; BRN.NHA;<br><br>**H3K27ac** BLD.CD14.MONO; BRN.NHA; BONE.OSTEO	**H3K4me3** IPSC.DF.19.11; SKIN.PEN.FRSK.FIB.01; SKIN.PEN.FRSK.FIB.02; SKIN.PEN.FRSK.MEL.01; SPLN<br><br>**H3K9ac**<br><br>CRVX.HELAS3.CNCR; BRN.NHA;	
rs568686	**11_TxEnh3**<br><br>(IPSC.15b; ESDR.H1.NEUR.PROG; SKIN.PEN.FRSK.FIB.01; SKIN.PEN.FRSK.FIB.02; SKIN.PEN.FRSK.MEL.01; SKIN.PEN.FRSK.KER.02; BRN.GANGEM.DR.NRSPHR; BRN.CRTX.DR.NRSPHR; BRN.HIPP.MID; BRN.SUB.NIG; BRN.ANT.CAUD; BRN.CING.GYR; BRN.INF.TMP;	None	ESC.WA7; ESC.4STAR; PSC.DF.6.9; IPSC.DF.19.11; ESDR.H1.NEUR.PROG; ESDR.H1.MSC; BLD.CD3.PPC; BLD.CD56.PC; SKIN.PEN.FRSK.FIB.01; SKIN.PEN.FRSK.FIB.02; SKIN.PEN.FRSK.MEL.01; BRST.MYO; BRN.GANGEM.DR.NRSPHR; BRN.CRTX.DR.NRSPHR;	ESC.H9; IPSC.DF.19.11; ESDR.H1.NEUR.PROG; ESDR.H1.MSC; SKIN.PEN.FRSK.FIB.01; SKIN.PEN.FRSK.FIB.02; SKIN.PEN.FRSK.MEL.01;	ESC.H9; IPSC.20B; IPSC.DF.19.11; ESDR.H1.NEUR.PROG; ESDR.CD56.ECTO; ESDR.CD184.ENDO; BLD.CD4.CD25I.CD127.TMEMPC; BLD.CD4.CD25M.CD45RO.MPC; BLD.MOB.CD34.PC.F; SKIN.PEN.FRSK.FIB.01; SKIN.PEN.FRSK.FIB.02;	LNG.IMR90; ESC.HUES6; IPSC.15b; ESDR.H1.NEUR.PROG; STRM.CHON.MRW.DR.MSC; BRN.SUB.NIG; BRN.ANT.CAUD; BRN.CING.GYR;	**H3K4me1** MUS.HSMM; MUS.HSMMT;<br><br>**H3K27ac** MUS.HSMMT; BLD.CD14.MONO;	**H3K4me3** None<br><br>**H3K9ac** None	

	BRN.ANG.GYR; BRN.DL.PRFRNTL.CRTX; BRN.GRM.MTRX; BRN.FET.F; MUS.TRNK.FET; MUS.LEG.FET; GI.STMC.FET; ADRL.GLND.FET; SPLN)		BRN.HIPP.MID; BRN.SUB.NIG; BRN.ANT.CAUD; BRN.INF.TMP; BRN.DL.PRFRNTL.CRTX; BRN.GRM.MTRX; MUS.SKLT.M; MUS.TRNK.FET; MUS.LEG.FET; HRT.ATR.R; HRT.VNT.R; GI.DUO.SM.MUS; GI.STMC.MUS; GI.STMC.FET; GI.S.INT.FET; GI.CLN.MUC; GI.RECT.MUC.29; GI.RECT.MUC.31; GI.STMC.MUC; GI.DUO.MUC; GI.ESO; GI.STMC.GAST; ADRL.GLND.FET; PANC; LNG; SPLN	BRN.GRM.MTRX; GI.STMC.MUS; GI.STMC.GAST; SPLN	SKIN.PEN.FRSK.MEL.01; BRN.HIPP.MID; BRN.SUB.NIG; BRN.ANT.CAUD; BRN.CING.GYR; BRN.INF.TMP; BRN.ANG.GYR; BRN.DL.PRFRNTL.CRTX; MUS.TRNK.FET; MUS.LEG.FET; HRT.VNT.R; GI.CLN.SM.MUS; GI.STMC.MUS; GI.STMC.FET; GI.ESO; ADRL.GLND.FET; LNG; SPLN	BRN.INF.TMP; BRN.ANG.GYR; BRN.DL.PRFRNTL.CRTX; MUS.SKLT.M; GI.STMC.MUS; GI.STMC.MUC; PANC.ISLT; ADRL.GLND.FET;	BRN.NHA; BONE.OSTEO		
rs604337	**17_EnhW2** (LNG.IMR90; ESDR.H1.MSC; STRM.MRW.MSC; STRM.CHON.MRW.DR.MSC; FAT.ADIP.DR.MSC; FAT.MSC.DR.ADIP; MUS.SAT; SKIN.PEN.FRSK.FIB.01; SKIN.PEN.FRSK.FIB.02)	**16_EnhW1** (LNG.A549.ETOH002.CNCR; BRN.NHA)<br><br>**14_EnhA2** (CRVX.HELAS3.CNCR; BONE.OSTEO)<br><br>**17_EnhW2** (MUS.HSMM; MUS.HSMMT; VAS.HUVEC; SKIN.NHDFAD; LNG.NHLF)	LNG.IMR90; ESDR.H9.NEUR; STRM.MRW.MSC; STRM.CHON.MRW.DR.MSC; FAT.ADIP.DR.MSC; MUS.SAT; SKIN.PEN.FRSK.FIB.01; SKIN.PEN.FRSK.FIB.02; BRN.CRTX.DR.NRSPHR; BRN.HIPP.MID; BRN.CING.GYR; BRN.DL.PRFRNTL.CRTX; BRN.GRM.MTRX; VAS.AOR; GI.DUO.SM.MUS; GI.S.INT.FET; GI.S.INT	FAT.ADIP.DR.MSC; MUS.SAT; BRN.HIPP.MID; BRN.CING.GYR; BRN.DL.PRFRNTL.CRTX; GI.STMC.MUS; LIV.ADLT; PANC; LNG; SPLN	BRN.SUB.NIG; BRN.ANT.CAUD; BRN.CING.GYR; BRN.INF.TMP; BRN.ANG.GYR; BRN.DL.PRFRNTL.CRTX; GI.CLN.SM.MUS;	BLD.PER.MONUC.PC; STRM.MRW.MSC; STRM.CHON.MRW.DR.MSC; FAT.ADIP.DR.MSC; FAT.MSC.DR.ADIP; MUS.SAT; BRN.SUB.NIG; BRN.ANT.CAUD; BRN.INF.TMP; BRN.DL.PRFRNTL.CRTX; MUS.SKLT.F; MUS.SKLT.M; GI.CLN.SM.MUS; GI.STMC.MUS; LIV.ADLT;	**H3K4me1**<br><br>LNG.A549.ETOH002.CNCR; CRVX.HELAS3.CNCR; MUS.HSMM; MUS.HSMMT; VAS.HUVEC; BRN.NHA; SKIN.NHDFAD; LNG.NHLF; BONE.OSTEO<br><br>**H3K27ac**<br>LNG.A549.ETOH002.CNCR;	**H3K4me3**<br>LNG.A549.ETOH002.CNCR; BRN.NHA; BONE.OSTEO<br><br>**H3K9ac**<br>LNG.A549.ETOH002.CNCR; MUS.HSMM; BLD.CD14.MONO; BRN.NHA;	
rs669426	**19_DNase** (MUS.PSOAS; VAS.AOR; GI.L.INT.FET; GI.CLN.SIG; GI.DUO.MUC; GI.STMC.GAST; VAS.HUVEC; BLD.K562.CNCR)	**19_DNase** (LNG.A549.ETOH002.CNCR; LIV.HEPG2.	SKIN.PEN.FRSK.KER.03; BRST.HMEC.35; BRN.ANT.CAUD; GI.DUO.SM.MUS; GI.S.INT.FET; GI.L.INT.FET; GI.S.INT; GI.RECT.MUC.31; GI.STMC.MUC; GI.DUO.MUC;	BRN.ANG.GYR; GI.RECT.MUC.29; GI.DUO.MUC;	GI.S.INT.FET; GI.L.INT.FET; GI.S.INT; OVRY;	BLD.PER.MONUC.PC; BLD.CD4.NPC; STRM.CHON.MRW.DR.MSC; FAT.MSC.DR.AD	**H3K4me1**<br>CRVX.HELAS3.CNCR;<br><br>**H3K27ac**	**H3K4me3**<br>None<br><br>**H3K9ac**<br>None	

	**17_EhW2**<br>(GI.S.INT.FET; GI.S.INT; GI.STMC.MUC)	CNCR; SKIN.NHEK)<br><br>**18_EnhAc** (CRVX.HELAS3.CNCR)				IP; BRST.MYO; BRN.ANT.CAUD; BRN.CING.GYR; BRN.INF.TMP; BRN.ANG.GYR; MUS.SKLT.F; GI.CLN.SM.MUS; GI.STMC.MUS; GI.DUO.MUC;	None		
rs865774	**11_TxEnh3**<br>(SKIN.PEN.FRSK.FIB.01; MUS.LEG.FET)<br><br>**10_TxEnh5**<br>(SKIN.PEN.FRSK.FIB.02; GI.STMC.FET)	None	ESC.I3; IPSC.DF.19.11; ESDR.H1.MSC; BLD.CD3.PPC; BLD.CD8.MPC; BLD.CD56.PC; SKIN.PEN.FRSK.FIB.01; SKIN.PEN.FRSK.FIB.02; SKIN.PEN.FRSK.MEL.01; SKIN.PEN.FRSK.KER.03; THYM.FET; BRN.ANG.GYR; BRN.DL.PRFRNTL.CRTX; MUS.TRNK.FET; MUS.LEG.FET; GI.STMC.MUS; GI.STMC.FET; GI.RECT.MUC.29; GI.STMC.GAST; PLCNT.AMN; ADRL.GLND.FET; PLCNT.FET;	ESDR.H1.MSC; SKIN.PEN.FRSK.FIB.01; SKIN.PEN.FRSK.FIB.02; SKIN.PEN.FRSK.MEL.01;<br><br>BRN.DL.PRFRNTL.CRTX; GI.STMC.GAST; PLCNT.AMN;	LNG.IMR90; IPSC.20B; ESDR.CD184.ENDO; BLD.CD3.PPC; BLD.CD4.CD25I.CD127.TMEMPC; BLD.CD4.CD25M.TPC; BLD.CD4.CD25M.CD45RA.NPC; BLD.CD4.CD25M.IL17M.PL.TPC; BLD.CD4.CD25M.CD45RO.MPC; BLD.MOB.CD34.PC.F; BLD.CD19.PPC; SKIN.PEN.FRSK.FIB.01; SKIN.PEN.FRSK.FIB.02; SKIN.PEN.FRSK.MEL.01; BRN.HIPP.MID; BRN.SUB.NIG; BRN.ANT.CAUD; BRN.CING.GYR; BRN.ANG.GYR; BRN.DL.PRFRNTL.CRTX; MUS.LEG.FET; HRT.VNT.R; GI.CLN.SM.MUS; GI.STMC.MUS; GI.STMC.FET; GI.RECT.MUC.29; GI.ESO; GI.STMC.GAST; PLCNT.AMN; KID.FET; LNG.FET; OVRY; PANC.ISLT; SPLN	ESC.HUES64; IPSC.18; IPSC.15b; ESDR.H1.NEUR.PROG; BLD.CD4.NPC; STRM.CHON.MRW.DR.MSC; BRN.SUB.NIG; BRN.INF.TMP; FAT.ADIP.NUC; GI.CLN.MUC;	**H3K4me1**<br>BLD.K562.CNCR<br><br>**H3K27ac**<br>BLD.DND41.CNCR; CRVX.HELAS3.CNCR; BLD.CD14.MONO; BRN.NHA; BONE.OSTEO	**H3K4me3**<br>None<br><br>**H3K9ac**<br>CRVX.HELAS3.CNCR; BRN.NHA;	
rs2781814	None	None	BLD.CD4.CD25M.IL17P.PL.TPC; BLD.CD8.NPC; BRN.CRTX.DR.NRSPHR; BRN.HIPP.MID; BRN.ANT.CAUD; BRN.CING.GYR; BRN.DL.PRFRNTL.CRTX; MUS.SKLT.F GI.DUO.SM.MUS; GI.CLN.SM.MUS; GI.S.INT.FET;	SKIN.PEN.FRSK.FIB.02; BRN.ANG.GYR; MUS.SKLT.F; GI.DUO.SM.MUS; GI.DUO.MUC;	BRN.INF.TMP; BRN.DL.PRFRNTL.CRTX; GI.DUO.SM.MUS; OVRY;	BLD.CD4.NPC;<br><br>BRN.ANT.CAUD; BRN.CING.GYR; BRN.INF.TMP; BRN.ANG.GYR;	**H3K4me1**<br>CRVX.HELAS3.CNCR;<br><br>**H3K27ac**<br>None	**H3K4me3**<br>None<br><br>**H3K9ac**<br>none	

			GI.L.INT.FET; GI.S.INT;<br>GI.RECT.MUC.29;<br>GI.RECT.MUC.31; GI.STMC.MUC;<br>GI.DUO.MUC; GI.ESO;			MUS.SKLT.M;<br>GI.CLN.SM.MUS; GI.STMC.MUS;<br>GI.STMC.MUC;<br>GI.DUO.MUC;<br>LNG.FET;<br>LIV.ADLT;			
rs2781815	None	None	BLD.CD4.CD25M.IL17P.PL.TPC;<br>BLD.CD8.NPC;<br>BRN.CRTX.DR.NRSPHR;<br>BRN.HIPP.MID; BRN.ANT.CAUD;<br>BRN.CING.GYR;<br>BRN.DL.PRFRNTL.CRTX;<br>MUS.SKLT.F; GI.DUO.SM.MUS;<br>GI.CLN.SM.MUS; GI.S.INT.FET;<br>GI.L.INT.FET; GI.S.INT;<br>GI.RECT.MUC.29;<br>GI.RECT.MUC.31; GI.STMC.MUC;<br>GI.DUO.MUC; GI.ESO;	SKIN.PEN.FRSK.FIB.02;<br>BRN.ANG.GYR; MUS.SKLT.F;<br>GI.DUO.SM.MUS;<br>GI.DUO.MUC;	BRN.INF.TMP;<br>BRN.DL.PRFRNTL.CRTX;<br>GI.DUO.SM.MUS; OVRY;	BLD.CD4.NPC;<br>BRN.ANT.CAUD;<br>BRN.CING.GYR;<br>BRN.INF.TMP;<br>BRN.ANG.GYR;<br>MUS.SKLT.M;<br>GI.CLN.SM.MUS; GI.STMC.MUS;<br>GI.STMC.MUC;<br>GI.DUO.MUC;<br>LNG.FET;<br>LIV.ADLT;	**H3K4me1**<br>CRVX.HELAS3.CNCR;<br><br>**H3K27ac**<br>None	**H3K4me3**<br>None<br><br>**H3K9ac**<br>none	
rs3850616	**19_DNase**<br>(LNG.IMR90; ESC.WA7; ESC.H9; ESC.I3; ESC.HUES6; ESC.HUES48; ESC.HUES64; ESC.H1; ESC.4STAR; IPSC.20B; IPSC.18; IPSC.15b; PSC.DF.6.9; IPSC.DF.19.11; ESDR.H1.NEUR.PROG; ESDR.H9.NEUR.PROG; ESDR.H9.NEUR; ESDR.CD56.MESO; ESDR.CD56.ECTO; ESDR.CD184.ENDO; ESDR.H1.BMP4.MESO; ESDR.H1.BMP4.TROP; ESDR.H1.MSC; STRM.MRW.MSC; STRM.CHON.MRW.DR.MSC; FAT.ADIP.DR.MSC; FAT.MSC.DR.ADIP; MUS.SAT; SKIN.PEN.FRSK.MEL.03; SKIN.PEN.FRSK.KER.03; BRST.HMEC.35; BRN.GANGEM.DR.NRSPHR; BRN.CRTX.DR.NRSPHR; BRN.GRM.MTRX; BRN.FET.F; BRN.FET.M; FAT.ADIP.NUC; MUS.PSOAS; MUS.TRNK.FET; HRT.FET; HRT.ATR.R; HRT.VENT.L; VAS.AOR; GI.CLN.SM.MUS; GI.STMC.FET; GI.S.INT.FET; GI.S.INT; GI.CLN.SIG; GI.ESO; GI.STMC.GAST; PLCNT.AMN; KID.FET; OVRY; PANC.ISLT; LIV.ADLT; PANC)	**19_DNase**<br>(LNG.A549.ETOH002.CNCR;<br>CRVX.HELAS3.CNCR;<br>BRST.HMEC;<br>MUS.HSMM;<br>MUS.HSMMT;<br>VAS.HUVEC; BRN.NHA;<br>SKIN.NHDFAD;<br>SKIN.NHEK;<br>LNG.NHLF;<br>BONE.OSTEO)<br><br>**16_EnhW1**<br>(BLD.DND41.CNCR;<br>BLD.GM12878;	LNG.IMR90; ESC.WA7;<br>ESC.I3; ESC.HUES6; ESC.HUES48;<br>ESC.H1; IPSC.15b; IPSC.DF.19.11;<br>ESDR.H9.NEUR.PROG;<br>ESDR.CD56.ECTO;<br>ESDR.H1.BMP4.TROP;<br>ESDR.H1.MSC; BLD.CD3.PPC;<br>BLD.CD4.CD25.CD127M.TREGPC;<br>BLD.CD4.CD25M.TPC;<br>BLD.CD4.CD25M.CD45RA.NPC;<br>BLD.CD4.CD25M.IL17M.PL.TPC;<br>BLD.CD4.CD25M.IL17P.PL.TPC;<br>BLD.CD4.MPC; BLD.CD8.MPC;<br>BLD.CD4.NPC; BLD.CD14.PC;<br>BLD.CD34.PC;<br>BLD.MOB.CD34.PC.M;<br>BLD.MOB.CD34.PC.F;<br>BLD.CD34.CC; BLD.CD19.PPC;<br>BLD.CD56.PC; BLD.CD15.PC;<br>FAT.ADIP.DR.MSC;<br>SKIN.PEN.FRSK.FIB.01;<br>SKIN.PEN.FRSK.FIB.02;<br>SKIN.PEN.FRSK.MEL.03;<br>BRST.MYO; THYM.FET;<br>BRN.HIPP.MID; BRN.SUB.NIG;<br>BRN.ANT.CAUD; BRN.CING.GYR;	ESDR.H1.NEUR.PROG;<br>BLD.CD4.MPC;<br>BLD.MOB.CD34.PC.M;<br>BLD.MOB.CD34.PC.F;<br>SKIN.PEN.FRSK.FIB.01;<br>SKIN.PEN.FRSK.FIB.02;<br>SKIN.PEN.FRSK.MEL.01;<br>BRN.HIPP.MID; SPLN	BLD.CD8.MPC;<br>SKIN.PEN.FRSK.FIB.02;<br>BRN.ANT.CAUD; HRT.VNT.R;<br>GI.RECT.MUC.31;	ESDR.H1.NEUR.PROG;<br>BLD.CD4.NPC;<br>STRM.CHON.MRW.DR.MSC;<br>FAT.ADIP.DR.MSC;<br>BRN.SUB.NIG;<br>BRN.ANT.CAUD;<br>BRN.CING.GYR;<br>BRN.INF.TMP;<br>BRN.DL.PRFRNTL.CRTX;<br>MUS.SKLT.M;<br>GI.DUO.MUC;	**H3K4me1**<br>BLD.DND41.CNCR;<br>BLD.GM12878;<br>CRVX.HELAS3.CNCR;<br>BLD.K562.CNCR;<br>BLD.CD14.MONO;<br><br>**H3K27ac**<br>None	**H3K4me3**<br>LNG.A549.ETOH002.CNCR;<br>BLD.DND41.CNCR;<br>BLD.GM12878;<br>CRVX.HELAS3.CNCR;<br>LIV.HEPG2.CNCR;<br>BRST.HMEC;<br>MUS.HSMM;<br>MUS.HSMMT;<br>VAS.HUVEC;<br>BLD.K56	ROADMAP<br>LNG.IMR90; ESC.H9;<br>ESC.H1;<br>PSC.DF.6.9;<br>IPSC.DF.19.11;<br>ESDR.H1.NEUR.PROG;<br>ESDR.H1.BMP4.MESO;<br>ESDR.H1.MSC;<br>BLD.CD3.PPC;<br>BLD.CD3.CPC;<br>BLD.CD14.PC;<br>BLD.MOB.CD34.PC.M;<br>BLD.MOB.CD34.PC.F;<br>BLD.CD19.PPC;

	**16_EnhW1**<br>(BLD.PER.MONUC.PC;<br>BLD.CD4.CD25I.CD127.TMEMPC;<br>BLD.CD4.CD25.CD127M.TREGPC;<br>BLD.CD4.CD25M.TPC;<br>BLD.CD4.CD25M.CD45RA.NPC;<br>BLD.CD4.CD25M.IL17M.PL.TPC;<br>BLD.CD4.CD25M.IL17P.PL.TPC;<br>BLD.CD4.CD25M.CD45RO.MPC;<br>BLD.CD4.MPC; BLD.CD8.MPC;<br>BLD.CD4.NPC; BLD.CD14.PC;<br>BLD.CD19.CPC; BLD.CD34.CC;<br>BLD.CD19.PPC; BLD.CD56.PC;<br>BLD.CD15.PC; SKIN.PEN.FRSK.FIB.01;<br>SKIN.PEN.FRSK.FIB.02;<br>SKIN.PEN.FRSK.MEL.01; BRST.MYO;<br>THYM.FET; BRN.HIPP.MID;<br>BRN.SUB.NIG; BRN.ANT.CAUD;<br>BRN.INF.TMP; BRN.DL.PRFRNTL.CRTX;<br>MUS.SKLT.F; MUS.SKLT.M;<br>MUS.LEG.FET; HRT.VNT.R;<br>GI.DUO.SM.MUS; GI.STMC.MUS;<br>GI.L.INT.FET; GI.CLN.MUC;<br>GI.RECT.MUC.29; GI.STMC.MUC;<br>PLCNT.FET; LNG; SPLN)<br><br>**14_EnhW1**<br>(BLD.CD3.PPC; BLD.MOB.CD34.PC.F;<br>BRN.CING.GYR; GI.DUO.MUC)<br><br>**17_EnhW2**<br>(BLD.CD3.CPC; BLD.CD8.NPC;<br>BLD.CD34.PC; SKIN.PEN.FRSK.KER.02;<br>THYM; BRN.ANG.GYR; GI.RECT.SM.MUS;<br>GI.RECT.MUC.31; LNG.FET;<br>ADRL.GLND.FET)<br><br>**13_EnhA1**<br>(BLD.MOB.CD34.PC.M)	BLD.K562.CNCR;<br>BLD.CD14.MONO)<br><br>**17_EnhW2**<br>(LIV.HEPG2.CNCR)	BRN.INF.TMP;<br>BRN.DL.PRFRNTL.CRTX;<br>FAT.ADIP.NUC;<br>MUS.SKLT.F; MUS.SKLT.M;<br>MUS.TRNK.FET; MUS.LEG.FET;<br>HRT.VNT.R; GI.STMC.MUS;<br>GI.STMC.FET; GI.S.INT.FET;<br>GI.L.INT.FET; GI.CLN.MUC;<br>GI.RECT.MUC.29;<br>GI.RECT.MUC.31; GI.STMC.MUC;<br>GI.DUO.MUC; GI.ESO;<br>GI.STMC.GAST; PLCNT.FET;<br>LIV.ADLT; LNG; SPLN					2.CNCR;<br>BLD.CD14.MONO;<br>BRN.NHA;<br>SKIN.NHDFAD;<br>SKIN.NHEK;<br>LNG.NHLF;<br>BONE.OSTEO<br><br>**H3K9ac**<br>CRVX.HELAS3.CNCR;	BLD.CD56.PC;<br>SKIN.PEN.FRSK.FIB.01;<br>SKIN.PEN.FRSK.FIB.02;<br>SKIN.PEN.FRSK.MEL.01;<br>BRST.HMEC.35;<br>THYM.FET;<br>BRN.FET.F;<br>BRN.FET.M;<br>MUS.PSOAS;<br>MUS.TRNK.FET;<br>MUS.LEG.FET;<br>HRT.FET;<br>GI.STMC.FET;<br>GI.S.INT.FET; GI.S.INT;<br>GI.STMC.GAST;<br>KID.FET;<br>LNG.FET;<br>OVRY;<br>ADRL.GLND.FET;<br>PLCNT.FET;<br>PANC;<br>ENCODE<br>LNG.A549.ETOH002.CNCR;<br>BLD.GM12878;<br>CRVX.HELAS3.CNCR;<br>BRST.HMEC;<br>MUS.HSMM;<br>MUS.HSM

									MT;<br>VAS.HUVEC;<br>BLD.K562.CNCR;<br>BLD.CD14.MONO;<br>SKIN.NHDFAD;<br>SKIN.NHEK;<br>LNG.NHLF;
rs17024628	**15_EnhAF**<br>(LNG.IMR90; ESC.HUES6; BRN.INF.TMP; BRN.ANG.GYR; GI.DUO.SM.MUS)<br><br>**17_EnhW2**<br>(ESC.WA7; ESC.H9; ESC.I3; ESC.HUES48; ESC.HUES64; ESC.H1; IPSC.20B; IPSC.18; IPSC.15b; PSC.DF.6.9; IPSC.DF.19.11; ESDR.H1.NEUR.PROG; ESDR.CD56.MESO; ESDR.CD56.ECTO; ESDR.CD184.ENDO; ESDR.H1.BMP4.MESO; BLD.CD15.PC; STRM.MRW.MSC; STRM.CHON.MRW.DR.MSC; FAT.MSC.DR.ADIP; SKIN.PEN.FRSK.MEL.01; SKIN.PEN.FRSK.MEL.03; SKIN.PEN.FRSK.KER.02; SKIN.PEN.FRSK.KER.03; BRST.HMEC.35; BRST.MYO; BRN.HIPP.MID; BRN.ANT.CAUD; BRN.CING.GYR; BRN.DL.PRFRNTL.CRTX; MUS.SKLT.F; MUS.SKLT.M; MUS.TRNK.FET; MUS.LEG.FET; HRT.FET; GI.CLN.SM.MUS; GI.RECT.SM.MUS; GI.STMC.FET; GI.L.INT.FET; GI.S.INT; GI.CLN.SIG; GI.CLN.MUC; GI.RECT.MUC.29; GI.RECT.MUC.31; PLCNT.AMN; LNG.FET; OVRY; ADRL.GLND.FET; LIV.ADLT; LNG)<br><br>**16_EnhW1**<br>(ESC.4STAR; ESDR.H1.MSC; GI.DUO.MUC)<br><br>**13_EnhA1**<br>(FAT.ADIP.DR.MSC; GI.STMC.MUC)	**2_PromU**<br>(LNG.A549.ETOH002.CNCR; CRVX.HELAS3.CNCR)<br><br>**17_EnhW2**<br>(BRST.HMEC; MUS.HSMM; MUS.HSMMT; VAS.HUVEC; BRN.NHA; SKIN.NHDFAD; LNG.NHLF;<br><br>16_EnhW1<br>SKIN.NHEK)<br><br>**13_EnhA1**<br>(LIV.HEPG2.CNCR)<br><br>**14_EnhA2**<br>(BLD.K562.CNCR; BONE.OSTEO)	LNG.IMR90; ESC.WA7; ESC.I3; ESC.HUES6; ESC.HUES48; ESC.HUES64; ESC.H1; ESC.4STAR; IPSC.20B; IPSC.18; IPSC.15b; IPSC.DF.19.11; ESDR.H1.NEUR.PROG; ESDR.H9.NEUR.PROG; ESDR.CD56.ECTO; ESDR.CD184.ENDO; ESDR.H1.BMP4.MESO; ESDR.H1.BMP4.TROP; ESDR.H1.MSC; BLD.CD3.PPC; BLD.CD4.CD25I.CD127.TMEMPC; BLD.CD14.PC; BLD.MOB.CD34.PC.F; STRM.MRW.MSC; STRM.CHON.MRW.DR.MSC; FAT.ADIP.DR.MSC; FAT.MSC.DR.ADIP; MUS.SAT; SKIN.PEN.FRSK.FIB.01; SKIN.PEN.FRSK.FIB.02; SKIN.PEN.FRSK.KER.03; BRST.HMEC.35; BRN.HIPP.MID; BRN.ANT.CAUD; BRN.INF.TMP; BRN.ANG.GYR; BRN.GRM.MTRX; FAT.ADIP.NUC; MUS.SKLT.F; MUS.SKLT.M; MUS.TRNK.FET; MUS.LEG.FET; HRT.ATR.R; HRT.VENT.L; HRT.VNT.R; GI.DUO.SM.MUS; GI.CLN.SM.MUS;	ESC.WA7; ESDR.H1.MSC; SKIN.PEN.FRSK.FIB.02; GI.STMC.MUS;	LNG.IMR90;<br><br>ESC.HUES48; ESC.H1; IPSC.20B; IPSC.DF.19.11; ESDR.CD56.MESO; ESDR.CD184.ENDO; ESDR.H1.BMP4.MESO; ESDR.H1.MSC; BLD.CD4.CD25I.CD127.TMEMPC; SKIN.PEN.FRSK.FIB.02; SKIN.PEN.FRSK.MEL.03; BRN.ANT.CAUD; BRN.CING.GYR; BRN.INF.TMP; BRN.ANG.GYR; BRN.DL.PRFRNTL.CRTX; FAT.ADIP.NUC; HRT.ATR.R; GI.DUO.SM.MUS; GI.RECT.SM.MUS; GI.STMC.MUS; GI.S.INT; GI.CLN.SIG; GI.CLN.MUC; OVRY; LNG;	ESC.I3; ESC.HUES6; IPSC.20B; IPSC.15b; BLD.PER.MONUC.PC; BLD.CD8.NPC; BRN.CING.GYR; BRN.ANG.GYR; MUS.SKLT.M; GI.STMC.MUS; GI.STMC.MUC; GI.DUO.MUC;	**H3K4me1**<br>LNG.A549.ETOH002.CNCR; CRVX.HELAS3.CNCR; LIV.HEPG2.CNCR; MUS.HSMMT; BLD.K562.CNCR; BLD.CD14.MONO; BRN.NHA; SKIN.NHDFAD; LNG.NHLF; BONE.OSTEO<br><br>**H3K27ac**<br>LNG.A549.ETOH002.CNCR; CRVX.HELAS3.CNCR; LIV.HEPG2.CNCR; BONE.OSTEO	**H3K4me3**<br>LNG.A549.ETOH002.CNCR; CRVX.HELAS3.CNCR; LIV.HEPG2.CNCR; BONE.OSTEO<br><br>**H3K9ac**<br>LNG.A549.ETOH002.CNCR; CRVX.HELAS3.CNCR; LIV.HEPG2.CNCR;	ENCODE<br><br>CRVX.HELAS3.CNCR

	**14_EnhA2**<br>(MUS.SAT; SKIN.PEN.FRSK.FIB.01; SKIN.PEN.FRSK.FIB.02; FAT.ADIP.NUC; GI.STMC.MUS)		GI.STMC.MUS; GI.STMC.FET; GI.S.INT.FET; GI.L.INT.FET; GI.S.INT; GI.CLN.MUC; GI.RECT.MUC.29; GI.RECT.MUC.31; GI.STMC.MUC; GI.DUO.MUC; GI.ESO; PLCNT.AMN; OVRY; LIV.ADLT; SPLN						
rs58434753	None	None	SKIN.PEN.FRSK.FIB.02; MUS.TRNK.FET; SPLN	None	SKIN.PEN.FRSK.FIB.02; MUS.TRNK.FET; SPLN	GI.STMC.MUS;	**H3K4me1**<br>None<br><br>**H3K27ac**<br>None	**H3K4me3**<br>None<br><br>**H3K9ac**<br>CRVX.HELAS3.CNCR;	
rs148898127	None	None	SKIN.PEN.FRSK.FIB.02;<br><br>MUS.TRNK.FET; SPLN	None	SKIN.PEN.FRSK.FIB.02;	None	**H3K4me1**<br>None<br><br>**H3K27ac**<br>none	**H3K4me3**<br>None<br><br>**H3K9ac**<br>CRVX.HELAS3.CNCR;	
rs201520834	None	None	BLD.CD4.CD25M.IL17P.PL.TPC; BLD.CD8.NPC; BRN.CRTX.DR.NRSPHR; BRN.HIPP.MID; BRN.ANT.CAUD; BRN.CING.GYR; BRN.DL.PRFRNTL.CRTX; MUS.SKLT.F; GI.DUO.SM.MUS; GI.CLN.SM.MUS; GI.S.INT.FET; GI.L.INT.FET; GI.S.INT; GI.RECT.MUC.29; I.RECT.MUC.31; GI.STMC.MUC; GI.DUO.MUC; GI.ESO;	SKIN.PEN.FRSK.FIB.02; BRN.ANG.GYR; BRN.DL.PRFRNTL.CRTX; MUS.SKLT.F; GI.DUO.SM.MUS; GI.DUO.MUC;	BRN.INF.TMP; BRN.DL.PRFRNTL.CRTX; GI.DUO.SM.MUS; GI.RECT.MUC.31; OVRY;	BLD.CD4.NPC; BRN.ANT.CAUD; BRN.CING.GYR; BRN.INF.TMP; BRN.ANG.GYR; MUS.SKLT.M; GI.CLN.SM.MUS; GI.STMC.MUS; GI.STMC.MUC; GI.DUO.MUC; LNG.FET; LIV.ADLT;	**H3K4me1**<br>CRVX.HELAS3.CNCR;<br><br>**H3K27ac**<br>None	**H3K4me3**<br>None<br><br>**H3K9ac**<br>None	
**rs1869959**	**10_TxEnh5**<br>(MUS.SKLT.F; MUS.LEG.FET;<br>11_TxEnh3<br>MUS.TRNK.FET;<br>18_EnhAc<br>GI.STMC.FET)	None	IPSC.15b; BLD.CD3.PPC; BLD.CD4.CD25.CD127M.TREGPC; BLD.CD4.CD25M.TPC; BLD.CD4.CD25M.CD45RA.NPC; BLD.CD4.CD25M.IL17M.PL.TPC;	None	LNG.IMR90; ESDR.CD56.ECTO; ESDR.CD184.ENDO; BLD.CD4.CD25I.CD127.TMEMPC; BLD.CD4.CD25.CD127M.TREGPC; BLD.CD4.CD25M.TPC;	ESC.H1; BRST.MYO; BRN.ANG.GYR; MUS.SKLT.M; HRT.FET;	**H3K4me1**<br>MUS.HSMMT;<br><br>**H3K27ac**<br>MUS.HSMMT;	**H3K4me3**<br><br>**None**<br><br><br>**H3K9ac**	

			BLD.CD4.NPC; BRN.HIPP.MID; BRN.SUB.NIG; BRN.ANT.CAUD; BRN.ANG.GYR; MUS.SKLT.F; MUS.TRNK.FET; MUS.LEG.FET; HRT.VNT.R; GI.S.INT.FET; GI.CLN.MUC; GI.STMC.MUC; PANC;		BLD.CD4.CD25M.CD45RA.NPC; BLD.CD4.CD25M.IL17M.PL.TPC; BLD.CD4.CD25M.IL17P.PL.TPC; BLD.CD4.CD25M.CD45RO.MPC; BLD.CD4.MPC; BLD.CD4.NPC; BLD.MOB.CD34.PC.F; SKIN.PEN.FRSK.FIB.02; BRN.HIPP.MID; BRN.SUB.NIG; BRN.CING.GYR; MUS.SKLT.F; MUS.TRNK.FET; MUS.LEG.FET; HRT.VNT.R;		BLD.CD14.MONO	CRVX.HELAS3.CNCR; MUS.HSMMT;	
rs4886613	None	None	BLD.CD4.CD25M.CD45RA.NPC; BLD.CD8.NPC; MUS.TRNK.FET; GI.L.INT.FET; GI.CLN.MUC; GI.STMC.MUC; PANC.ISLT;	None	BLD.CD3.PPC; BLD.CD4.CD25.CD127M.TREGPC; BLD.CD4.CD25M.TPC; BLD.CD4.CD25M.CD45RA.NPC; BLD.CD4.CD25M.IL17M.PL.TPC; BLD.CD4.NPC; BLD.MOB.CD34.PC.F; SKIN.PEN.FRSK.FIB.02; BRN.HIPP.MID; BRN.CING.GYR; MUS.SKLT.F; HRT.VNT.R; GI.CLN.MUC; GI.RECT.MUC.29; PANC.ISLT;	BLD.PER.MONUC.PC; BLD.CD4.NPC; STRM.CHON.MRW.DR.MSC; BRST.MYO; BRN.INF.TMP; BRN.ANG.GYR; FAT.ADIP.NUC; GI.CLN.MUC; PANC.ISLT;	**H3K4me1**<br>BLD.GM12878;<br><br>**H3K27ac**<br>None	**H3K4me3**<br>None<br><br>**H3K9ac**<br>None	
rs936230	**11_TxEnh3** (SKIN.PEN.FRSK.FIB.02)	None	IPSC.DF.19.11; ESDR.H1.BMP4.TROP; ESDR.H1.MSC; BLD.PER.MONUC.PC; BLD.CD3.PPC; BLD.CD4.CD25M.CD45RA.NPC; SKIN.PEN.FRSK.FIB.01; SKIN.PEN.FRSK.KER.03; THYM.FET; MUS.SKLT.F; MUS.TRNK.FET; MUS.LEG.FET; GI.S.INT.FET; GI.L.INT.FET; GI.RECT.MUC.29;	IPSC.DF.19.11; ESDR.H1.NEUR.PROG; ESDR.H1.MSC; SKIN.PEN.FRSK.FIB.02; SKIN.PEN.FRSK.MEL.01; SPLN	ESDR.CD184.ENDO; BLD.CD3.PPC; BLD.CD4.CD25I.CD127.TMEMPC; BLD.CD4.CD25.CD127M.TREGPC; BLD.CD4.CD25M.TPC; BLD.CD4.CD25M.CD45RA.NPC; BLD.CD4.CD25M.IL17M.PL.TPC; BLD.CD4.CD25M.IL17P.PL.TPC;	IPSC.15b; ESDR.H1.NEUR.PROG; ESDR.H1.MSC; BLD.PER.MONUC.PC; BLD.CD4.NPC; STRM.CHON.MRW.DR.MSC; BRST.MYO; BRN.ANT.CAUD;	**H3K4me1**<br>SKIN.NHEK;<br><br>**H3K27ac**<br>MUS.HSMMT; BLD.CD14.MONO;	**H3K4me3**<br>None<br><br>**H3K9ac**<br>CRVX.HELAS3.CNCR;	Roadmap MUS.PSOAS<br><br>ENCODE LIV.HEPG2.CNCR

			GI.STMC.MUC; GI.DUO.MUC;<br>GI.ESO; GI.STMC.GAST;<br>SPLN		BLD.CD4.CD25M.CD45RO.MPC; BLD.CD4.MPC;<br>BLD.CD4.NPC;<br>BLD.MOB.CD34.PC.F;<br>SKIN.PEN.FRSK.FIB.01;<br>SKIN.PEN.FRSK.FIB.02;<br>SKIN.PEN.FRSK.MEL.01;<br>SKIN.PEN.FRSK.MEL.03;<br>SKIN.PEN.FRSK.KER.03;<br>THYM; BRN.HIPP.MID;<br>BRN.SUB.NIG;<br>BRN.CING.GYR;<br>MUS.SKLT.F;<br>MUS.TRNK.FET;<br>HRT.VNT.R;<br>GI.CLN.SM.MUS;<br>GI.S.INT; GI.RECT.MUC.29;<br>LNG;	BRN.CING.GYR;<br>BRN.INF.TMP;<br>BRN.ANG.GYR;<br>MUS.SKLT.F;<br>MUS.SKLT.M;<br>GI.STMC.MUS;<br>GI.RECT.MUC.29;<br>GI.DUO.MUC;			
rs7180432	**15_EnhAF**<br>(MUS.TRNK.FET;<br>17_EnhW2<br>MUS.LEG.FET;<br>12_TxEnhW2<br>GI.STMC.FET)	**17_EnhW2**<br>(BLD.GM12878)	ESDR.CD56.ECTO;<br>ESDR.H1.BMP4.TROP;<br>ESDR.H1.MSC;<br>BLD.PER.MONUC.PC;<br>BLD.CD3.PPC;<br>BLD.CD4.CD25I.CD127.TMEMPC;<br>BLD.CD4.CD25.CD127M.TREGPC;<br>BLD.CD4.CD25M.TPC;<br>BLD.CD4.CD25M.CD45RA.NPC;<br>BLD.CD4.CD25M.IL17M.PL.TPC;<br>BLD.CD4.CD25M.IL17P.PL.TPC;<br>BLD.CD4.CD25M.CD45RO.MPC;<br>BLD.CD4.MPC; BLD.CD8.MPC;<br>BLD.CD4.NPC; BLD.CD8.NPC;<br>BLD.MOB.CD34.PC.M;<br>BLD.MOB.CD34.PC.F;<br>BLD.CD34.CC; BLD.CD19.PPC;<br>BLD.CD56.PC;<br>SKIN.PEN.FRSK.FIB.02;<br>SKIN.PEN.FRSK.MEL.01;<br>SKIN.PEN.FRSK.KER.03;<br>BRST.HMEC.35;<br>THYM.FET; BRN.HIPP.MID;<br>BRN.SUB.NIG; BRN.ANT.CAUD;<br>BRN.CING.GYR; BRN.ANG.GYR;<br>BRN.DL.PRFRNTL.CRTX;<br>BRN.FET.M; FAT.ADIP.NUC;<br>MUS.PSOAS; MUS.SKLT.F;<br>MUS.SKLT.M; MUS.TRNK.FET;	BRN.ANT.CAUD;<br>HRT.FET;<br>GI.RECT.SM.MUS;	ESDR.CD56.ECTO;<br>ESDR.CD184.ENDO;<br>BLD.PER.MONUC.PC;<br>BLD.CD4.CD25I.CD127.TMEMPC;<br>BLD.CD4.CD25.CD127M.TREGPC;<br>BLD.CD4.CD25M.CD45RA.NPC;<br>BLD.CD4.CD25M.IL17M.PL.TPC;<br>BLD.CD4.CD25M.IL17P.PL.TPC;<br><br>BLD.CD4.MPC;<br>BLD.CD4.NPC;<br>BLD.MOB.CD34.PC.F;<br>BLD.CD19.PPC;<br>BLD.CD56.PC;<br>SKIN.PEN.FRSK.FIB.01;<br>SKIN.PEN.FRSK.MEL.01;<br>SKIN.PEN.FRSK.MEL.03;<br>SKIN.PEN.FRSK.KER.03;<br>BRN.HIPP.MID;<br>BRN.SUB.NIG;<br>BRN.ANT.CAUD;<br>BRN.CING.GYR;<br>BRN.INF.TMP;	ESC.HUES64;<br>IPSC.15b;<br>BLD.PER.MONUC.PC;<br>BLD.CD4.NPC;<br>BRN.ANT.CAUD;<br>BRN.ANG.GYR;<br>BRN.DL.PRFRNTL.CRTX;<br>FAT.ADIP.NUC;<br>MUS.SKLT.F;<br>MUS.SKLT.M;<br>HRT.FET;<br>GI.RECT.SM.MUS;<br>GI.STMC.MUS;<br>GI.RECT.MUC.29;<br>GI.RECT.MUC.31;<br>GI.STMC.MUC;	**H3K4me1**<br>LNG.A549.ETOH002.CNCR;<br>BLD.GM12878;<br>MUS.HSMMT;<br>SKIN.NHEK;<br><br>**H3K27ac**<br>BLD.DND41.CNCR;<br>BLD.GM12878;<br>CRVX.HELAS3.CNCR;<br>MUS.HSMMT;<br>BLD.CD14.MONO;	**H3K4me3**<br>BLD.GM12878;<br><br>**H3K9ac**<br><br>BLD.GM12878;<br>CRVX.HELAS3.CNCR;<br>MUS.HSMMT;<br>SKIN.NHEK;<br>LNG.NHLF;<br>BONE.OSTEO	

			MUS.LEG.FET; HRT.FET; HRT.ATR.R; HRT.VENT.L; HRT.VNT.R; GI.DUO.SM.MUS; GI.CLN.SM.MUS; GI.STMC.MUS; GI.S.INT.FET; GI.L.INT.FET; GI.S.INT; GI.CLN.MUC; GI.RECT.MUC.29; GI.RECT.MUC.31; GI.STMC.MUC; GI.DUO.MUC; GI.ESO; GI.STMC.GAST; PLCNT.AMN; PLCNT.FET; LIV.ADLT; PANC; LNG; SPLN		BRN.ANG.GYR; BRN.DL.PRFRNTL.CRTX;<br><br>MUS.PSOAS; MUS.SKLT.F;<br><br>MUS.TRNK.FET; MUS.LEG.FET; HRT.ATR.R; HRT.VENT.L; HRT.VNT.R; GI.S.INT; GI.CLN.MUC; GI.RECT.MUC.29; GI.RECT.MUC.31; GI.ESO; ADRL.GLND.FET; SPLN				
**rs67931591**	None	None	None	None	None	None	**H3K4me1** BRN.NHA; BONE.OSTEO<br><br>**H3K27ac** None	**H3K4me3** None<br><br>**H3K9ac** None	
**rs1637365**	None	None	None	None	None	None	**H3K4me1**<br><br>**H3K27ac**	**H3K4me3**<br><br>**H3K9ac**	
**rs181337095**	None	None	None	None	None	None	**H3K4me1** BRN.NHA; BONE.OSTEO<br><br>**H3K27ac**	**H3K4me3**<br><br>**H3K9ac**	
**rs60381548**	None	None	BLD.CD3.PPC; BLD.CD4.CD25I.CD127.TMEMPC; BLD.CD4.CD25.CD127M.TREGPC; BLD.CD4.CD25M.TPC; BLD.CD4.CD25M.CD45RA.NPC; BLD.CD4.CD25M.IL17M.PL.TPC;	GI.S.INT;	ESC.H9; ESC.HUES6; BLD.CD4.CD25.CD127M.TREGPC; BLD.CD4.CD25M.IL17M.PL.TPC; BLD.CD8.MPC; GI.STMC.GAST; LNG;	ESDR.H1.NEUR.PROG; ESDR.H1.MSC;	**H3K4me1** None<br><br>**H3K27ac** BLD.CD14.MONO;	**H3K4me3** None<br><br>**H3K9ac** None	

			BLD.CD4.CD25M.IL17P.PL.TPC; BLD.CD4.CD25M.CD45RO.MPC; BLD.CD4.MPC; BLD.CD8.MPC; BLD.CD4.NPC; BLD.CD8.NPC; BLD.CD56.PC; SKIN.PEN.FRSK.KER.03; BRST.HMEC.35; BRST.MYO; BRN.DL.PRFRNTL.CRTX; MUS.PSOAS; MUS.SKLT.F; HRT.VENT.L; HRT.VNT.R; VAS.AOR; GI.DUO.SM.MUS; GI.STMC.MUS; GI.ESO; GI.STMC.GAST; LNG.FET; OVRY; PANC; LNG;				SKIN.NHEK; LNG.NHLF; BONE.OSTEO		

*Highlighted in yellow are breast tissues; in red are the 25-state model active enhancers and promoters and DNase for the breast tissues

Supplementary table 7B: Detailed functional annotation for novel loci and correlated SNPs (r^2 >0.8) - Africans

SNP	Roadmap 25-state model using 12 imputed marks	ENCODE 25-state model using 12 imputed marks	Roadmap H3K4me1	Roadmap H3K4me3	Roadmap H3K27ac	Roadmap H3K9ac	ENCODE Enhancers H3K4me1 and H3K27ac	ENCODE Promoters H3K4me3 and H3K9ac	DNase
**rs2522057**	**22_PromP** (ESDR.H9.NEUR.PROG; ESDR.CD56.MESO; BRN.CRTX.DR.NRSPHR; BRN.GRM.MTRX)<br><br>**19_DNase** (ESDR.H9.NEUR)<br><br>17_EnhW2 ESDR.H1.BMP4.TROP; SKIN.PEN.FRSK.FIB.02; SKIN.PEN.FRSK.MEL.03; OVRY; PANC.ISLT;<br><br>**15_EnhAF** (ESDR.H1.MSC; FAT.ADIP.DR.MSC; FAT.MSC.DR.ADIP; SKIN.PEN.FRSK.FIB.01; SKIN.PEN.FRSK.MEL.01; HRT.ATR.R; HRT.VENT.L; HRT.VNT.R; GI.STMC.GAST; PANC; LNG)<br><br>**13_EnhA1** (BLD.PER.MONUC.PC; BLD.CD4.CD25I.CD127.TMEMPC; BLD.CD3.CPC; BLDBLD.CD4.CD25M.CD45RA.NPC; BLD.CD4.CD25M.IL17P.PL.TPC; BLD.CD8.NPC; BLD.CD34.PC; STRM.MRW.MSC; THYM; FAT.ADIP.NUC; GI.DUO.SM.MUS; GI.S.INT; GI.CLN.SIG; GI.RECT.MUC.31; LIV.ADLT;<br><br>3_PromD1 (BLD.CD3.PPC; BLD.CD4.CD25.CD127M.TREGPC; BLD.CD4.CD25M.TPC;	**2_PromU** (BLD.GM12878; BLD.CD14.MONO); 13_EnhA1 (LIV.ADLT; BLD.DND41.CNCR; MUS.HSMMT; BLD.K562.CNCR)<br><br>**14_EnhA2** (LIV.HEPG2.CNCR; BRST.HMEC; MUS.HSMM; VAS.HUVEC; SKIN.NHEK; BONE.OSTEO); 15_EnhAF (LNG.A549.ETOH002.CNCR; BRN.NHA; SKIN.NHDFAD; LNG.NHLF); **17_EnhW2** (CRVX.HELAS3.CNCR)	LNG.IMR90; ESC.WA7; ESC.H9; ESC.I3; ESC.HUES6; ESC.HUES48; ESC.H1; IPSC.20B; IPSC.15b; IPSC.DF.19.11; ESDR.CD56.MESO; ESDR.H1.BMP4.TROP; ESDR.H1.MSC; BLD.PER.MONUC.PC; BLD.CD3.PPC; BLD.CD4.CD25I.CD127.TMEMPC; BLD.CD3.CPC; BLD.CD4.CD25.CD127M.TREGPC; BLD.CD4.CD25M.TPC; BLD.CD4.CD25M.CD45RA.NPC; BLD.CD4.CD25M.IL17M.PL.TPC; BLD.CD4.CD25M.IL17P.PL.TPC; BLD.CD4.CD25M.CD45RO.MPC; BLD.CD4.MPC; BLD.CD8.MPC; BLD.CD4.NPC; BLD.CD8.NPC; BLD.CD14.PC; BLD.CD19.CPC; BLD.CD34.PC; BLD.MOB.CD34.PC.M; BLD.MOB.CD34.PC.F; BLD.CD34.CC; BLD.CD19.PPC; BLD.CD56.PC; BLD.CD15.PC; STRM.MRW.MSC; STRM.CHON.MRW.DR.MSC; MUS.SAT; SKIN.PEN.FRSK.FIB.01; SKIN.PEN.FRSK.FIB.02; SKIN.PEN.FRSK.MEL.01; SKIN.PEN.FRSK.MEL.03; SKIN.PEN.FRSK.KER.02; SKIN.PEN.FRSK.KER.03; BRST.HMEC.35; BRST.MYO; BRN.GANGEM.DR.NRSPHR; BRN.CRTX.DR.NRSPHR; THYM; THYM.FET; BRN.HIPP.MID; BRN.SUB.NIG; BRN.ANT.CAUD; BRN.CING.GYR; BRN.DL.PRFRNTL.CRTX;	BLD.PER.MONUC.PC; BLD.CD3.PPC; BLD.CD4.CD25I.CD127.TMEMPC; BLD.CD3.CPC; BLD.CD4.CD25.CD127M.TREGPC; BLD.CD4.CD25M.TPC; BLD.CD4.CD25M.CD45RA.NPC; BLD.CD4.CD25M.IL17M.PL.TPC; BLD.CD4.CD25M.IL17P.PL.TPC; BLD.CD4.CD25M.CD45RO.MPC; BLD.CD4.MPC; BLD.CD8.MPC; BLD.CD4.NPC; BLD.CD8.NPC; BLD.CD14.PC; BLD.CD19.CPC; BLD.CD34.PC; BLD.MOB.CD34.PC.M; BLD.MOB.CD34.PC.F; BLD.CD34.CC; BLD.CD19.PPC;	IPSC.DF.19.11; BLD.PER.MONUC.PC; BLD.CD3.PPC; BLD.CD4.CD25I.CD127.TMEMPC; BLD.CD3.CPC; BLD.CD4.CD25.CD127M.TREGPC; BLD.CD4.CD25M.TPC; BLD.CD4.CD25M.CD45RA.NPC; BLD.CD4.CD25M.IL17M.PL.TPC; BLD.CD4.CD25M.IL17P.PL.TPC; BLD.CD4.CD25M.CD45RO.MPC; BLD.CD4.MPC; BLD.CD8.MPC; BLD.CD4.NPC; BLD.CD8.NPC; BLD.CD14.PC; BLD.CD19.CPC; BLD.CD34.PC; BLD.MOB.CD34.PC.M; BLD.MOB.CD34.PC.F; BLD.CD34.CC; BLD.CD19.PPC; BLD.CD56.PC; BLD.CD15.PC; STRM.MRW.MSC; MUS.SAT; SKIN.PEN.FRSK.FIB.02; SKIN.PEN.FRSK.KER.02; THYM; THYM.FET; BRN.HIPP.MID; BRN.ANT.CAUD; FAT.ADIP.NUC; MUS.SKLT.F; GI.DUO.SM.MUS; GI.CLN.SM.MUS; GI.RECT.SM.MUS; GI.STMC.MUS; GI.CLN.SIG; GI.CLN.MUC; GI.RECT.MUC.29; GI.RECT.MUC.31;	ESC.HUES6; BLD.PER.MONUC.PC; BLD.CD4.NPC; BLD.CD8.NPC; STRM.MRW.MSC; FAT.MSC.DR.ADIP; MUS.SAT; BRN.ANT.CAUD; BRN.INF.TMP; BRN.ANG.GYR; BRN.DL.PRFRNTL.CRTX; BRN.DL.PRFRNTL.CRTX; FAT.ADIP.NUC; MUS.SKLT.F; MUS.SKLT.M ; GI.CLN.SM.MUS; GI.RECT.SM.MUS; GI.STMC.MUS; GI.CLN.MUC; GI.RECT.MUC.29; GI.RECT.MUC.31; GI.STMC.MUC; GI.DUO.MUC; PANC.ISLT; LIV.ADLT	**H3K4me1**<br><br>LNG.A549.ETOH002.CNCR; BLD.DND41.CNCR; BLD.GM12878; CRVX.HELAS3.CNCR; LIV.HEPG2.CNCR; BRST.HMEC; MUS.HSMM; MUS.HSMMT; VAS.HUVEC; BLD.K562.CNCR; BLD.CD14.MONO; BRN.NHA; SKIN.NHDFAD; SKIN.NHEK; LNG.NHLF; BONE.OSTEO	**H3K4me3**<br><br>BLD.DND41.CNCR; BLD.GM12878; MUS.HSMMT; BLD.CD14.MONO; SKIN.NHEK; BONE.OSTEO.<br><br>**H3K9ac**<br><br>LNG.A549.ETOH002.CNCR;	ROADMAP BLD.CD3.PPC; BLD.CD3.CPC; BLD.MOB.CD34.PC.M; BLD.MOB.CD34.PC.F; BLD.CD19.PPC; BLD.CD56.PC; SKIN.PEN.FRSK.KER.02; THYM.FET;<br><br>ENCODE BLD.GM12878; SKIN.NHEK

	BLD.CD4.CD25M.IL17M.PL.TPC; BLD.CD4.CD25M.CD45RO.MPC; BLD.CD4.MPC; BLD.CD8.MPC; BLD.CD4.NPC; BLD.CD19.CPC; BLD.MOB.CD34.PC.M; BLD.MOB.CD34.PC.F; BLD.CD34.CC; BLD.CD19.PPC; BLD.CD56.PC; BLD.CD15.PC; THYM.FET)<br><br>**2_PromU**<br>(BLD.CD14.PC)<br><br>**9_TxReg**<br>(GI.RECT.MUC.29; GI.DUO.MUC; SPLN)<br><br>**14_EnhA2**<br>(STRM.CHON.MRW.DR.MSC; MUS.SAT; SKIN.PEN.FRSK.KER.02; SKIN.PEN.FRSK.KER.03; BRST.HMEC.35; BRST.MYO; BRN.HIPP.MID; BRN.DL.PRFRNTL.CRTX; MUS.SKLT.F; MUS.SKLT.M; MUS.TRNK.FET; MUS.LEG.FET; GI.CLN.SM.MUS; GI.RECT.SM.MUS; GI.STMC.MUS; GI.S.INT.FET; GI.L.INT.FET; GI.CLN.MUC; GI.STMC.MUC; GI.DUO.MUC; KID.FET; LNG.FET; ADRL.GLND.FET; PLCNT.FET)<br><br>**16_EnhW1**<br>(BRN.SUB.NIG; BRN.ANT.CAUD; BRN.CING.GYR; BRN.INF.TMP; BRN.ANG.GYR; BRN.FET.M; MUS.PSOAS; GI.STMC.FET; PLCNT.AMN)		FAT.ADIP.NUC; MUS.PSOAS; MUS.SKLT.F; MUS.TRNK.FET; MUS.LEG.FET; HRT.FET; HRT.ATR.R; HRT.VENT.L; HRT.VNT.R; GI.DUO.SM.MUS; GI.CLN.SM.MUS; GI.RECT.SM.MUS; GI.STMC.MUS; GI.STMC.FET; GI.S.INT.FET; GI.L.INT.FET; GI.S.INT; GI.CLN.SIG; GI.CLN.MUC; GI.RECT.MUC.29; GI.RECT.MUC.31; GI.STMC.MUC; GI.DUO.MUC; GI.ESO; GI.STMC.GAST; PLCNT.AMN; LNG.FET; OVRY; ADRL.GLND.FET; PLCNT.FET; LIV.ADLT; PANC; LNG; SPLN	BLD.CD56.PC; BLD.CD15.PC; STRM.MRW.MSC; MUS.SAT; SKIN.PEN.FRSK.FIB.02; SKIN.PEN.FRSK.KER.02; THYM; THYM.FET; BRN.HIPP.MID; BRN.ANT.CAUD; FAT.ADIP.NUC; MUS.SKLT.F; GI.DUO.SM.MUS; GI.CLN.SM.MUS; GI.RECT.SM.MUS; GI.STMC.MUS; GI.CLN.SIG; GI.CLN.MUC; GI.RECT.MUC.29; GI.RECT.MUC.31; GI.STMC.MUC; GI.DUO.MUC; GI.ESO; LIV.ADLT.	GI.STMC.MUC; GI.DUO.MUC; GI.ESO; LIV.ADLT.		**H3K27ac**<br>BLD.DND41.CNCR; BLD.GM12878; MUS.HSMMT; BLD.K562.CNCR; BLD.CD14.MONO; SKIN.NHEK; BONE.OSTEO.	BLD.DND41.CNCR; BLD.GM12878; CRVX.HELAS3.CNCR; LIV.HEPG2.CNCR; BRST.HMEC; MUS.HSMM; MUS.HSMMT; VAS.HUVEC; BLD.K562.CNCR; BLD.CD14.MONO; BRN.NHA; SKIN.NHDFAD; SKIN.NHEK; LNG.NHLF; BONE.OSTEO	
rs2706396	**12_TxEnhW**<br>(BLD.PER.MONUC.PC; BLD.CD3.CPC; THYM; BRN.HIPP.MID)<br><br>**9_TxReg**<br>(BLD.CD3.PPC; BLD.CD56.PC; BLD.CD15.PC; GI.DUO.MUC; LIV.ADLT;<br><br>10_TxEnh5<br>BLD.CD4.CD25I.CD127.TMEMPC; BLD.CD4.CD25.CD127M.TREGPC; BLD.CD4.CD25M.TPC;	**13_EnhA1**<br>(BLD.DND41.CNCR)<br><br>**10_TxEnhW2**<br>(BLD.GM12878)<br><br>**17_EnhW2**	LNG.IMR90; IPSC.DF.19.11; ESDR.CD56.MESO; ESDR.CD56.ECTO; ESDR.H1.BMP4.TROP; ESDR.H1.MSC; BLD.PER.MONUC.PC; BLD.CD3.PPC; BLD.CD4.CD25I.CD127.TMEMPC; BLD.CD3.CPC; BLD.CD4.CD25.CD127M.TREGPC; BLD.CD4.CD25M.TPC; BLD.CD4.CD25M.CD45RA.NPC;	BLD.CD4.CD25I.CD127.TMEMPC; BLD.CD4.CD25M.IL17P.PL.TPC; BLD.CD4.CD25M.CD45RO.MPC; BLD.CD4.MPC; BLD.CD8.MPC; BLD.MOB.CD3	ESDR.H1.MSC; BLD.PER.MONUC.PC; BLD.CD3.PPC; BLD.CD4.CD25I.CD127.TMEMPC; BLD.CD4.CD25.CD127M.TREGPC; BLD.CD4.CD25M.TPC; BLD.CD4.CD25M.CD45RA.NPC; BLD.CD4.CD25M.IL17M.PL.TPC; BLD.CD4.CD25M.IL17P.PL.T	ESDR.H1.NEUR.PROG; BLD.PER.MONUC.PC; BLD.CD4.NPC; BRN.ANG.GYR; FAT.ADIP.NUC; MUS.SKLT.M; HRT.FET; GI.CLN.SM.MUS; GI.STMC.MUS; GI.CLN.MUC;	**H3K4me1**<br>LNG.A549.ETOH002.CNCR; BLD.DND41.CNCR; BLD.GM12878; CRVX.HELAS3.CNCR; LIV.HEPG2	**H3K4me3**<br><br>BLD.CD14.MONO.<br><br>**H3K9ac**<br>BLD.DND41.CNCR; BLD.CD1	

	BLD.CD4.CD25M.CD45RA.NPC; BLD.CD4.CD25M.IL17M.PL.TPC; BLD.CD4.CD25M.IL17P.PL.TPC; BLD.CD4.CD25M.CD45RO.MPC; BLD.CD4.MPC; BLD.CD8.MPC; BLD.CD4.NPC; BLD.CD8.NPC; BLD.CD14.PC; BLD.CD19.CPC; BLD.CD34.PC; BLD.MOB.CD34.PC.M; BLD.MOB.CD34.PC.F; BLD.CD34.CC; BLD.CD19.PPC; THYM.FET; GI.DUO.SM.MUS; GI.S.INT.FET; GI.S.INT; GI.RECT.MUC.29)<br><br>**17_EmhW2**<br>(BRST.MYO; BRN.SUB.NIG; BRN.ANT.CAUD; BRN.CING.GYR; BRN.INF.TMP; MUS.SKLT.F; MUS.SKLT.M; MUS.LEG.FET; HRT.VNT.R; GI.RECT.SM.MUS; GI.STMC.MUS; GI.CLN.MUC; GI.ESO; GI.STMC.GAST; KID.FET; LNG.FET; ADRL.GLND.FET; PANC; LNG; SPLN).<br><br>**15_EnhAF**<br>(FAT.ADIP.NUC; GI.CLN.SM.MUS; GI.L.INT.FET; GI.CLN.SIG; GI.RECT.MUC.31; GI.STMC.MUC)<br><br>**11_TxEnh3**<br>(GI.STMC.FET; PLCNT.FET)	(CRVX.HELAS3.CNCR; BLD.K562.CNCR)<br><br>**15_EnhAF**<br>(LIV.HEPG2.CNCR)<br><br>**9_TxReg**<br>(BLD.CD14.MONO)	BLD.CD4.CD25M.IL17M.PL.TPC; BLD.CD4.CD25M.IL17P.PL.TPC; BLD.CD4.CD25M.CD45RO.MPC; BLD.CD4.MPC; BLD.CD8.MPC; BLD.CD4.NPC; BLD.CD8.NPC; BLD.CD14.PC; BLD.CD19.CPC; BLD.CD34.PC; BLD.MOB.CD34.PC.M; BLD.MOB.CD34.PC.F; BLD.CD34.CC; BLD.CD19.PPC; BLD.CD56.PC; BLD.CD15.PC; STRM.MRW.MSC; SKIN.PEN.FRSK.FIB.01; SKIN.PEN.FRSK.FIB.02; SKIN.PEN.FRSK.KER.03; BRST.MYO; BRN.GANGEM.DR.NRSPHR; THYM; THYM.FET; BRN.HIPP.MID; BRN.SUB.NIG; BRN.ANT.CAUD; BRN.CING.GYR; BRN.DL.PRFRNTL.CRTX; FAT.ADIP.NUC; MUS.SKLT.F; MUS.SKLT.M; MUS.TRNK.FET; MUS.LEG.FET; HRT.FET; HRT.ATR.R; HRT.VENT.L; HRT.VNT.R; GI.DUO.SM.MUS; GI.CLN.SM.MUS; GI.RECT.SM.MUS; GI.STMC.MUS; GI.STMC.FET; GI.S.INT.FET; GI.L.INT.FET; GI.S.INT; GI.CLN.SIG; GI.CLN.MUC; GI.RECT.MUC.29; GI.RECT.MUC.31; GI.STMC.MUC; GI.DUO.MUC; GI.ESO; GI.STMC.GAST; LNG.FET; ADRL.GLND.FET; PLCNT.FET; LIV.ADLT; PANC; LNG; SPLN	4.PC.M; BLD.MOB.CD34.PC.F; BLD.CD15.PC; GI.DUO.SM.MUS; GI.CLN.SM.MUS; GI.RECT.SM.MUS; GI.STMC.MUS; GI.RECT.MUC.29; GI.STMC.MUC; GI.DUO.MUC; LIV.ADLT; PANC.	PC; BLD.CD4.CD25M.CD45RO.MPC; BLD.CD4.MPC; BLD.CD8.MPC; BLD.CD4.NPC; BLD.CD8.NPC; BLD.CD14.PC; BLD.MOB.CD34.PC.F; BLD.CD19.PPC; BLD.CD56.PC; SKIN.PEN.FRSK.FIB.01; THYM; THYM.FET; BRN.HIPP.MID; BRN.ANT.CAUD; BRN.CING.GYR; FAT.ADIP.NUC; MUS.PSOAS; MUS.SKLT.F; HRT.ATR.R; HRT.VNT.R; GI.DUO.SM.MUS; GI.RECT.SM.MUS; GI.STMC.MUS; GI.STMC.FET; GI.S.INT.FET; GI.L.INT.FET; GI.S.INT; GI.CLN.SIG; GI.CLN.MUC; GI.RECT.MUC.29; GI.RECT.MUC.31; GI.ESO; GI.STMC.GAST; PLCNT.FET; LIV.ADLT; LNG.	GI.RECT.MUC.29; GI.RECT.MUC.31; GI.STMC.MUC; GI.DUO.MUC; LNG.FET; LIV.ADLT.	.CNCR; BRST.HMEC; MUS.HSMMT; BLD.CD14.MONO; LNG.NHLF.<br><br>**H3K27ac**<br>BLD.DND41.CNCR; BLD.CD14.MONO; BONE.OSTEO	4.MONO.	
rs2522052	**17_EnhW2**<br>(ESDR.H1.BMP4.TROP; SKIN.PEN.FRSK.FIB.02; SKIN.PEN.FRSK.MEL.01; BRST.MYO; BRN.CRTX.DR.NRSPHR; BRN.SUB.NIG; BRN.ANT.CAUD; BRN.CING.GYR; BRN.INF.TMP; BRN.ANG.GYR; BRN.DL.PRFRNTL.CRTX; BRN.GRM.MTRX; BRN.FET.M; MUS.PSOAS; HRT.ATR.R;	**9_TxReg**<br>(BLD.DND41.CNCR)<br><br>**10_TxEnh5**<br>(BLD.GM12878)<br><br>**15_EnhAF**	ESC.4STAR; IPSC.20B; IPSC.15b; IPSC.DF.19.11; ESDR.CD56.ECTO; ESDR.H1.MSC; BLD.PER.MONUC.PC; BLD.CD3.PPC; BLD.CD4.CD25I.CD127.TMEMPC; BLD.CD3.CPC; BLD.CD4.CD25.CD127M.TREGPC; BLD.CD4.CD25M.TPC; BLD.CD4.CD25M.CD45RA.NPC;	BLD.CD4.CD25I.CD127.TMEMPC; BLD.CD4.CD25.CD127M.TREGPC; BLD.CD4.CD25M.IL17M.PL.TPC; BLD.CD4.CD25	BLD.PER.MONUC.PC; BLD.CD3.PPC; BLD.CD4.CD25I.CD127.TMEMPC; BLD.CD4.CD25.CD127M.TREGPC; BLD.CD4.CD25M.TPC; BLD.CD4.CD25M.CD45RA.NPC; BLD.CD4.CD25M.IL17M.PL.TPC;	ESC.HUES6; BRN.SUB.NIG; BRN.ANT.CAUD; BRN.INF.TMP; BRN.ANG.GYR; FAT.ADIP.NUC; GI.CLN.SM.MUS; GI.RECT.SM.MUS; GI.CLN.MUC;	**H3K4me1**<br>BLD.DND41.CNCR; BLD.GM12878; LIV.HEPG2.CNCR; MUS.HSMM; MUS.HSM	**H3K4me3**<br>BLD.DND41.CNCR; BLD.K562.CNCR; BLD.CD14.MONO;	

	GI.RECT.SM.MUS; GI.ESO; PLCNT.AMN;<br>KID.FET; LNG.FET; OVRY; PANC.ISLT)<br><br>**10_TxEnh5**<br>(BLD.PER.MONUC.PC; BLD.CD3.PPC;<br>BLD.CD4.CD25I.CD127.TMEMPC;<br>BLD.CD4.CD25.CD127M.TREGPC;<br>BLD.CD4.CD25M.TPC;<br>BLD.CD4.CD25M.CD45RA.NPC;<br>BLD.CD4.CD25M.IL17M.PL.TPC;<br>BLD.CD4.CD25M.IL17P.PL.TPC;<br>BLD.CD4.CD25M.CD45RO.MPC;<br>BLD.CD4.MPC; BLD.CD8.MPC;<br>BLD.CD4.NPC; BLD.CD8.NPC;<br>BLD.CD14.PC; BLD.CD19.CPC;<br>BLD.CD34.PC; BLD.CD19.PPC;<br>BLD.CD56.PC; THYM; THYM.FET;<br>MUS.LEG.FET; HRT.VNT.R;<br>GI.DUO.SM.MUS; GI.CLN.SM.MUS;<br>GI.STMC.FET; GI.CLN.SIG;<br>GI.RECT.MUC.29; SPLN)<br><br>**12_TxEnhW**<br>(BLD.CD3.CPC; SKIN.PEN.FRSK.FIB.01;<br>BRN.HIPP.MID; MUS.SKLT.M)<br><br>**9_TxReg**<br>(BLD.MOB.CD34.PC.M;<br>BLD.MOB.CD34.PC.F; BLD.CD34.CC;<br>BLD.CD15.PC; GI.S.INT.FET;<br>GI.DUO.MUC)<br><br>**15_EnhW2**<br>(FAT.ADIP.NUC; MUS.SKLT.F;<br>MUS.TRNK.FET; HRT.VENT.L; GI.S.INT;<br>GI.RECT.MUC.31; GI.STMC.MUC;<br>ADRL.GLND.FET; PANC; LNG)<br><br>**11_TxEnh3**<br>(GI.STMC.MUS; GI.STMC.GAST;<br>PLCNT.FET;<br><br>**13_EnhA1**<br>(GI.L.INT.FET; LIV.ADLT)<br><br>**14_EnhA2**<br>(GI.CLN.MUC)	(LIV.HEPG2.<br>CNCR)<br><br>**15_EnhAF**<br>(BLD.K562.<br>CNCR)<br><br>**10_TxEnhA<br>F**<br>(BLD.CD14.<br>MONO)	BLD.CD4.CD25M.IL17M.PL.TPC;<br>BLD.CD4.CD25M.IL17P.PL.TPC;<br>BLD.CD4.CD25M.CD45RO.MPC;<br>BLD.CD4.MPC; BLD.CD8.MPC;<br>BLD.CD4.NPC; BLD.CD8.NPC;<br>BLD.CD14.PC; BLD.CD19.CPC;<br>BLD.CD34.PC;<br>BLD.MOB.CD34.PC.M;<br>BLD.MOB.CD34.PC.F;<br>BLD.CD34.CC; BLD.CD19.PPC;<br>BLD.CD56.PC; BLD.CD15.PC;<br>SKIN.PEN.FRSK.FIB.02;<br>BRST.MYO; THYM; THYM.FET;<br>BRN.HIPP.MID; BRN.SUB.NIG;<br>BRN.ANT.CAUD; BRN.INF.TMP;<br>BRN.ANG.GYR;<br>BRN.DL.PRFRNTL.CRTX;<br>BRN.FET.F; BRN.FET.M;<br>FAT.ADIP.NUC; MUS.SKLT.F;<br>MUS.SKLT.M; MUS.TRNK.FET;<br>MUS.LEG.FET; HRT.ATR.R;<br>HRT.VENT.L; HRT.VNT.R;<br>GI.DUO.SM.MUS;<br>GI.CLN.SM.MUS;<br>GI.RECT.SM.MUS; GI.STMC.MUS;<br>GI.STMC.FET; GI.S.INT.FET;<br>GI.L.INT.FET; GI.S.INT; GI.CLN.SIG;<br>GI.CLN.MUC; GI.RECT.MUC.29;<br>GI.RECT.MUC.31; GI.STMC.MUC;<br>GI.DUO.MUC; GI.ESO;<br>GI.STMC.GAST; KID.FET; LNG.FET;<br>PANC.ISLT; PLCNT.FET; LIV.ADLT;<br>PANC; LNG; SPLN	M.IL17P.PL.TP<br>C;<br>BLD.CD4.MPC;<br>BLD.MOB.CD3<br>4.PC.F;<br>BLD.CD15.PC;<br>SKIN.PEN.FRS<br>K.FIB.01;<br>SKIN.PEN.FRS<br>K.FIB.02;<br>GI.DUO.SM.M<br>US;<br>GI.CLN.SM.M<br>US;<br>GI.RECT.SM.M<br>US;<br>GI.CLN.MUC;<br>GI.DUO.MUC;<br>LIV.ADLT;<br>SPLN	BLD.CD4.CD25M.IL17P.PL.T<br>PC;<br>BLD.CD4.CD25M.CD45RO.M<br>PC; BLD.CD4.MPC;<br>BLD.CD8.MPC;<br>BLD.CD4.NPC;<br>BLD.CD8.NPC;<br>BLD.MOB.CD34.PC.F;<br>BLD.CD19.PPC;<br>BLD.CD56.PC;<br>SKIN.PEN.FRSK.FIB.02;<br>THYM; THYM.FET;<br>BRN.HIPP.MID;<br>BRN.SUB.NIG;<br>BRN.ANT.CAUD;<br>BRN.CING.GYR;<br>BRN.INF.TMP;<br>BRN.DL.PRFRNTL.CRTX;<br>FAT.ADIP.NUC; MUS.PSOAS;<br>MUS.SKLT.F; MUS.LEG.FET;<br>HRT.VENT.L; HRT.VNT.R;<br>GI.DUO.SM.MUS;<br>GI.CLN.SM.MUS;<br>GI.STMC.MUS;<br>GI.STMC.FET; GI.S.INT.FET;<br>GI.L.INT.FET; GI.S.INT;<br>GI.CLN.SIG; GI.CLN.MUC;<br>GI.RECT.MUC.29;<br>GI.RECT.MUC.31; GI.ESO;<br>GI.STMC.GAST; PLCNT.FET;<br>LIV.ADLT; LNG; SPLN	GI.RECT.MUC.2<br>9;<br>GI.RECT.MUC.3<br>1;<br>GI.STMC.MUC;<br>GI.DUO.MUC;<br>GI.ESO;<br>LNG.FET;<br>PANC.ISLT;<br>LIV.ADLT.	MT;<br>BLD.CD14.<br>MONO.<br><br><br><br>**H3K27ac**<br>BLD.DND4<br>1.CNCR;<br>BLD.GM1<br>2878;<br>BLD.CD14.<br>MONO.	BRN.NH<br>A;<br>SKIN.NH<br>DFAD;<br>SKIN.NH<br>EK;<br>LNG.NHL<br>F;<br>BONE.O<br>STEO<br><br>**H3K9ac**<br>BLD.DND<br>41.CNCR<br>.	

rs2706336	**12_TxEnhW** (BLD.PER.MONUC.PC; BLD.CD3.CPC; THYM; BRN.HIPP.MID)<br><br>**9_TxReg** (BLD.CD3.PPC; BLD.CD56.PC; BLD.CD15.PC; GI.DUO.MUC; LIV.ADLT)<br><br>**10_TxEnhW** (BLD.CD4.CD25I.CD127.TMEMPC; BLD.CD4.CD25.CD127M.TREGPC; BLD.CD4.CD25M.TPC; BLD.CD4.CD25M.CD45RA.NPC; BLD.CD4.CD25M.IL17M.PL.TPC; BLD.CD4.CD25M.IL17P.PL.TPC; BLD.CD4.CD25M.CD45RO.MPC; BLD.CD4.MPC; BLD.CD8.MPC; BLD.CD4.NPC; BLD.CD8.NPC; BLD.CD14.PC; BLD.CD19.CPC; BLD.CD34.PC; BLD.MOB.CD34.PC.M; BLD.MOB.CD34.PC.F; BLD.CD34.CC; BLD.CD19.PPC; THYM.FET; GI.DUO.SM.MUS; GI.S.INT.FET; GI.S.INT; GI.RECT.MUC.29)<br><br>**17_EnhW2** (BRST.MYO; BRN.SUB.NIG; BRN.ANT.CAUD; BRN.CING.GYR; BRN.INF.TMP; MUS.SKLT.F; MUS.LEG.FET; MUS.SKLT.M; HRT.VNT.R; GI.RECT.SM.MUS; GI.STMC.MUS; GI.CLN.MUC; GI.ESO; GI.STMC.GAST; KID.FET; LNG.FET; ADRL.GLND.FET; PANC; LNG; SPLN)<br><br>**15_EnhAF** (FAT.ADIP.NUC; GI.CLN.SM.MUS; GI.L.INT.FET; GI.CLN.SIG; GI.RECT.MUC.31; GI.STMC.MUC;<br><br>**11_TxEnh3** GI.STMC.FET; PLCNT.FET)	**13_EnhA1** (BLD.DND41.CNCR)<br><br>**10_TxEnh5** (BLD.GM12878)<br><br>**17_EnhW2** (CRVX.HELAS3.CNCR; BLD.K562.CNCR)<br><br>**15_EnhAF** (LIV.HEPG2.CNCR)<br><br>**9_TxReg** (BLD.CD14.MONO)	LNG.IMR90; IPSC.DF.19.11; ESDR.CD56.MESO; ESDR.CD56.ECTO; ESDR.H1.BMP4.TROP; ESDR.H1.MSC; BLD.PER.MONUC.PC; BLD.CD3.PPC; BLD.CD4.CD25I.CD127.TMEMPC; BLD.CD3.CPC; BLD.CD4.CD25.CD127M.TREGPC; BLD.CD4.CD25M.TPC; BLD.CD4.CD25M.CD45RA.NPC; BLD.CD4.CD25M.IL17M.PL.TPC; BLD.CD4.CD25M.IL17P.PL.TPC; BLD.CD4.CD25M.CD45RO.MPC; BLD.CD4.MPC; BLD.CD8.MPC; BLD.CD4.NPC; BLD.CD8.NPC; BLD.CD14.PC; BLD.CD19.CPC; BLD.CD34.PC; BLD.MOB.CD34.PC.M; BLD.MOB.CD34.PC.F; BLD.CD34.CC; BLD.CD19.PPC; BLD.CD56.PC; BLD.CD15.PC; STRM.MRW.MSC; SKIN.PEN.FRSK.FIB.01; SKIN.PEN.FRSK.FIB.02; SKIN.PEN.FRSK.KER.03; BRST.MYO; BRN.GANGEM.DR.NRSPHR; THYM; THYM.FET; BRN.HIPP.MID; BRN.SUB.NIG; BRN.ANT.CAUD; BRN.CING.GYR; BRN.DL.PRFRNTL.CRTX; FAT.ADIP.NUC; MUS.SKLT.F; MUS.SKLT.M; MUS.TRNK.FET; MUS.LEG.FET; HRT.FET; HRT.ATR.R; HRT.VENT.L; HRT.VNT.R; GI.DUO.SM.MUS; GI.CLN.SM.MUS; GI.RECT.SM.MUS; GI.STMC.MUS; GI.STMC.FET; GI.S.INT.FET; GI.L.INT.FET; GI.S.INT; GI.CLN.SIG; GI.CLN.MUC; GI.RECT.MUC.29; GI.RECT.MUC.31; GI.STMC.MUC; GI.DUO.MUC; GI.ESO; GI.STMC.GAST; LNG.FET;	BLD.CD4.CD25I.CD127.TMEMPC; BLD.CD4.CD25M.IL17P.PL.TPC; BLD.CD4.CD25M.CD45RO.MPC; BLD.CD4.MPC; BLD.CD8.MPC; BLD.MOB.CD34.PC.M; BLD.MOB.CD34.PC.F; BLD.CD15.PC; GI.DUO.SM.MUS; GI.CLN.SM.MUS; GI.RECT.SM.MUS; GI.RECT.MUC.29; GI.STMC.MUC; GI.DUO.MUC; LIV.ADLT; PANC	ESDR.H1.MSC; BLD.PER.MONUC.PC; BLD.CD3.PPC; BLD.CD4.CD25I.CD127.TMEMPC; BLD.CD4.CD25.CD127M.TREGPC; BLD.CD4.CD25M.TPC; BLD.CD4.CD25M.CD45RA.NPC; BLD.CD4.CD25M.IL17M.PL.TPC; BLD.CD4.CD25M.IL17P.PL.TPC; BLD.CD4.CD25M.CD45RO.MPC; BLD.CD4.MPC; BLD.CD8.MPC; BLD.CD4.NPC; BLD.CD8.NPC; BLD.CD14.PC; BLD.MOB.CD34.PC.F; BLD.CD19.PPC; BLD.CD56.PC; SKIN.PEN.FRSK.FIB.01; THYM; THYM.FET; BRN.HIPP.MID; BRN.ANT.CAUD; BRN.CING.GYR; FAT.ADIP.NUC; MUS.PSOAS; MUS.SKLT.F; HRT.ATR.R; HRT.VNT.R; GI.DUO.SM.MUS; GI.RECT.SM.MUS; GI.STMC.MUS; GI.STMC.FET; GI.S.INT.FET; GI.L.INT.FET; GI.S.INT; GI.CLN.SIG; GI.CLN.MUC; GI.RECT.MUC.29; GI.RECT.MUC.31; GI.ESO; GI.STMC.GAST; PLCNT.FET; LIV.ADLT; LNG;	ESDR.H1.NEUR.PROG; BLD.PER.MONUC.PC; BLD.CD4.NPC; BRN.ANG.GYR; FAT.ADIP.NUC; MUS.SKLT.M; HRT.FET; GI.CLN.SM.MUS; GI.STMC.MUS; GI.CLN.MUC; GI.RECT.MUC.29; GI.RECT.MUC.31; GI.STMC.MUC; GI.DUO.MUC; LNG.FET; LIV.ADLT	**H3K4me1** LNG.A549.ETOH002.CNCR; BLD.DND41.CNCR; BLD.GM12878; CRVX.HELAS3.CNCR; LIV.HEPG2.CNCR; BRST.HMEC; MUS.HSMMT; BLD.CD14.MONO; LNG.NHLF<br><br>**H3K27ac** BLD.DND41.CNCR; BLD.CD14.MONO; BONE.OSTEO	**H3K4me3** BLD.CD14.MONO<br><br>**H3K9ac** BLD.DND41.CNCR; BLD.CD14.MONO	DNase ROADMAP GI.S.INT.FET, GI.L.INT.FET<br><br>ENCODE

			ADRL.GLND.FET; PLCNT.FET; LIV.ADLT; PANC; LNG; SPLN						
rs2248116	**17_EnhW2**<br>(ESDR.H1.MSC; BLD.PER.MONUC.PC; BLD.CD4.CD25M.IL17P.PL.TPC; BLD.CD8.MPC; BLD.CD4.NPC; BLD.CD8.NPC; BLD.CD34.PC; STRM.MRW.MSC; STRM.CHON.MRW.DR.MSC; FAT.MSC.DR.ADIP; MUS.SAT; SKIN.PEN.FRSK.FIB.01; SKIN.PEN.FRSK.FIB.02; BRST.HMEC.35; FAT.ADIP.NUC; GI.S.INT; GI.CLN.SIG; GI.CLN.MUC; GI.RECT.MUC.29; GI.RECT.MUC.31; GI.STMC.MUC; GI.DUO.MUC; SPLN)<br><br>**10_TxEnh5**<br>(BLD.CD3.PPC; BLD.CD4.CD25M.TPC; BLD.CD4.MPC; BLD.MOB.CD34.PC.F; BLD.CD19.PPC; BLD.CD56.PC; THYM.FET)<br><br>**12_TxEnhW**<br>(BLD.CD4.CD25I.CD127.TMEMPC; BLD.CD3.CPC; BLD.CD4.CD25.CD127M.TREGPC; BLD.CD4.CD25M.CD45RA.NPC; BLD.CD4.CD25M.IL17M.PL.TPC; BLD.CD4.CD25M.CD45RO.MPC; THYM)<br><br>**15_EnhAF**<br>(BLD.CD14.PC; BLD.CD19.CPC; BLD.MOB.CD34.PC.M; BLD.CD34.CC)<br><br>**9_TxReg**<br>(BLD.CD15.PC)	**17_EnhW2**<br>(BLD.DND41.CNCR; BRST.HMEC; MUS.HSMM; MUS.HSMMT; BLD.K562.CNCR; BRN.NHA; SKIN.NHDFAD; LNG.NHLF)<br><br>**12_TxEnhW**<br>(BLD.GM12878)<br><br>**10_TxEnh5**<br>(BLD.CD14.MONO)<br><br>**15_EnhAF**<br>(BONE.OSTEO)	LNG.IMR90; ESDR.CD56.MESO; BLD.PER.MONUC.PC; BLD.CD3.PPC; BLD.CD4.CD25I.CD127.TMEMPC; BLD.CD3.CPC; BLD.CD4.CD25.CD127M.TREGPC; BLD.CD4.CD25M.TPC; BLD.CD4.CD25M.CD45RA.NPC; BLD.CD4.CD25M.IL17M.PL.TPC; BLD.CD4.CD25M.IL17P.PL.TPC; BLD.CD4.CD25M.CD45RO.MPC; BLD.CD4.MPC; BLD.CD8.MPC; BLD.CD4.NPC; BLD.CD8.NPC; BLD.CD14.PC; BLD.CD19.CPC; BLD.CD34.PC; BLD.MOB.CD34.PC.M; BLD.MOB.CD34.PC.F; BLD.CD34.CC; BLD.CD19.PPC; BLD.CD56.PC; BLD.CD15.PC; STRM.MRW.MSC; STRM.CHON.MRW.DR.MSC; FAT.ADIP.DR.MSC; FAT.MSC.DR.ADIP; MUS.SAT; SKIN.PEN.FRSK.FIB.01; SKIN.PEN.FRSK.FIB.02; SKIN.PEN.FRSK.KER.02; SKIN.PEN.FRSK.KER.03; BRST.HMEC.35; BRST.MYO; THYM.FET; BRN.DL.PRFRNTL.CRTX; FAT.ADIP.NUC; GI.DUO.SM.MUS; GI.CLN.SM.MUS; GI.STMC.MUS; GI.STMC.FET; GI.S.INT.FET; GI.L.INT.FET; GI.S.INT; GI.CLN.MUC; GI.RECT.MUC.29; GI.RECT.MUC.31; GI.STMC.MUC; GI.DUO.MUC; PLCNT.FET; LIV.ADLT	BLD.CD4.CD25M.TPC; BLD.CD8.MPC; BLD.CD4.NPC; BLD.CD19.CPC; BLD.MOB.CD34.PC.M; BLD.CD19.PPC; BLD.CD15.PC; STRM.MRW.MSC; GI.DUO.SM.MUS; GI.CLN.SIG; GI.CLN.MUC; GI.DUO.MUC	ESDR.H1.MSC; BLD.PER.MONUC.PC; BLD.CD3.PPC; BLD.CD4.CD25I.CD127.TMEMPC; BLD.CD4.CD25.CD127M.TREGPC; BLD.CD4.CD25M.TPC; BLD.CD4.CD25M.CD45RA.NPC; BLD.CD4.CD25M.IL17M.PL.TPC; BLD.CD4.CD25M.IL17P.PL.TPC; BLD.CD4.CD25M.CD45RO.MPC; BLD.CD4.MPC; BLD.CD8.MPC; BLD.CD8.NPC; BLD.MOB.CD34.PC.F; BLD.CD19.PPC; BLD.CD56.PC; STRM.MRW.MSC; THYM; THYM.FET; FAT.ADIP.NUC; MUS.SKLT.F; MUS.TRNK.FET; GI.DUO.SM.MUS; GI.STMC.MUS; GI.L.INT.FET; GI.S.INT; GI.CLN.SIG; GI.CLN.MUC; GI.RECT.MUC.29; GI.ESO; ADRL.GLND.FET; PLCNT.FET; LIV.ADLT; PANC; SPLN	FAT.MSC.DR.ADIP; MUS.SAT; GI.RECT.MUC.29; GI.DUO.MUC	**H3K4me1**<br>BLD.DND41.CNCR; BLD.GM12878; CRVX.HELAS3.CNCR; LIV.HEPG2.CNCR; BRST.HMEC; MUS.HSMM; MUS.HSMMT; VAS.HUVEC; BLD.CD14.MONO; BRN.NHA; SKIN.NHDFAD; SKIN.NHEK; LNG.NHLF; BONE.OSTEO<br><br>**H3K27ac**<br>BLD.DND41.CNCR; BLD.GM12878; MUS.HSMM; MUS.HSMMT; BLD.CD14.MONO; LNG.NHLF;	**BLD.DND41.CNCR; MUS.HSMMT**	

							BONE.OSTEO		
**rs17024629**	**14_EnhA2** (FAT.ADIP.DR.MSC); 17_EnhW2 (STRM.CHON.MRW.DR.MSC; FAT.MSC.DR.ADIP; SKIN.PEN.FRSK.FIB.02; BRN.ANT.CAUD; FAT.ADIP.NUC; GI.DUO.SM.MUS;GI.STMC.MUC; GI.DUO.MUC) ; 18_EnhAc (SKIN.PEN.FRSK.FIB.01)	**13_EnhA2** (CRVX.HELAS3.CNCR); 14_EnhA2 (LNG.A549.ETOH002.CNCR; LIV.HEPG2.CNCR); 17EnhW2 (SKIN.NHDFAD; BONE.OSTEO)	LNG.IMR90; ESC.WA7; ESC.I3; ESC.HUES6; ESC.4STAR; IPSC.20B; IPSC.18; IPSC.15b; IPSC.DF.19.11; ESDR.CD56.ECTO; ESDR.CD184.ENDO; BLD.CD3.PPC; BLD.CD4.CD25I.CD127.TMEMPC; BLD.MOB.CD34.PC.F; STRM.MRW.MSC; FAT.ADIP.DR.MSC; FAT.MSC.DR.ADIP; MUS.SAT; SKIN.PEN.FRSK.FIB.01; SKIN.PEN.FRSK.FIB.02; BRN.HIPP.MID; BRN.ANT.CAUD; BRN.ANG.GYR; BRN.GRM.MTRX; FAT.ADIP.NUC; MUS.TRNK.FET; MUS.LEG.FET; HRT.ATR.R; HRT.VENT.L; HRT.VNT.R; GI.DUO.SM.MUS; GI.CLN.SM.MUS; GI.STMC.MUS; GI.STMC.FET; GI.S.INT.FET; GI.L.INT.FET; GI.S.INT; GI.CLN.MUC; GI.RECT.MUC.29; GI.STMC.MUC; GI.DUO.MUC; PLCNT.AMN	ESC.WA7; SKIN.PEN.FRSK.FIB.02; GI.STMC.MUS;	LNG.IMR90; ESC.HUES48; ESC.H1; IPSC.20B; IPSC.DF.19.11; ESDR.CD56.MESO; ESDR.H1.BMP4.MESO; ESDR.H1.MSC; BLD.CD4.CD25I.CD127.TMEMPC; SKIN.PEN.FRSK.FIB.02; BRN.ANT.CAUD; BRN.CING.GYR; BRN.INF.TMP; FAT.ADIP.NUC; HRT.ATR.R; GI.DUO.SM.MUS; GI.RECT.SM.MUS; GI.STMC.MUS; GI.S.INT; GI.CLN.SIG; LNG	ESC.HUES6; IPSC.15b; BLD.PER.MONUC.PC; BRN.CING.GYR; BRN.ANG.GYR; MUS.SKLT.M; GI.STMC.MUS; GI.STMC.MUC; GI.DUO.MUC	**H3K4me1** (LNG.A549.ETOH002.CNCR; CRVX.HELAS3.CNCR; LIV.HEPG2.CNCR; MUS.HSMMT; SKIN.NHDFAD; LNG.NHLF; BONE.OSTEO) **H3K27ac** LNG.A549.ETOH002.CNCR; CRVX.HELAS3.CNCR; LIV.HEPG2.CNCR	**H3K4me3** LNG.A549.ETOH002.CNCR; CRVX.HELAS3.CNCR; LIV.HEPG2.CNCR **H3K9ac** LNG.A549.ETOH002.CNCR; CRVX.HELAS3.CNCR; LIV.HEPG2.CNCR;	SKIN.PEN.FRSK.FIB.01
**rs58434753**	None	None	SKIN.PEN.FRSK.FIB.02; MUS.TRNK.FET; SPLN	None	SKIN.PEN.FRSK.FIB.02; MUS.TRNK.FET; SPLN	GI.STMC.MUS;	**H3K4me1** None **H3K27ac** None	**H3K4me3** None **H3K9ac** CRVX.HELAS3.CNCR;	
**rs148898127**	None	None	SKIN.PEN.FRSK.FIB.02; MUS.TRNK.FET; SPLN	None	SKIN.PEN.FRSK.FIB.02;	None	**H3K4me1** None **H3K27ac** none	**H3K4me3** None **H3K9ac** CRVX.HELAS3.CNCR;	

**rs1869959**	**10_TxEnh5** (MUS.SKLT.F; MUS.LEG.FET; 11_TxEnh3 MUS.TRNK.FET; 18_EnhAc GI.STMC.FET)	None	IPSC.15b; BLD.CD3.PPC; BLD.CD4.CD25.CD127M.TREGPC; BLD.CD4.CD25M.TPC; BLD.CD4.CD25M.CD45RA.NPC; BLD.CD4.CD25M.IL17M.PL.TPC; BLD.CD4.NPC; BRN.HIPP.MID; BRN.SUB.NIG; BRN.ANT.CAUD; BRN.ANG.GYR; MUS.SKLT.F; MUS.TRNK.FET; MUS.LEG.FET; HRT.VNT.R; GI.S.INT.FET; GI.CLN.MUC; GI.STMC.MUC; PANC;	None	LNG.IMR90; ESDR.CD56.ECTO; ESDR.CD184.ENDO; BLD.CD4.CD25I.CD127.TMEMPC; BLD.CD4.CD25.CD127M.TREGPC; BLD.CD4.CD25M.TPC; BLD.CD4.CD25M.CD45RA.NPC; BLD.CD4.CD25M.IL17M.PL.TPC; BLD.CD4.CD25M.IL17P.PL.TPC; BLD.CD4.CD25M.CD45RO.MPC; BLD.CD4.MPC; BLD.CD4.NPC; BLD.MOB.CD34.PC.F; SKIN.PEN.FRSK.FIB.02; BRN.HIPP.MID; BRN.SUB.NIG; BRN.CING.GYR; MUS.SKLT.F; MUS.TRNK.FET; MUS.LEG.FET; HRT.VNT.R;	ESC.H1; BRST.MYO; BRN.ANG.GYR; MUS.SKLT.M; HRT.FET;	**H3K4me1** MUS.HSMMT;<br><br>**H3K27ac** MUS.HSMMT; BLD.CD14.MONO	**H3K4me3**<br><br>**None**<br><br>**H3K9ac** CRVX.HELAS3.CNCR; MUS.HSMMT;	
**rs936230**	**11_TxEnh3** (SKIN.PEN.FRSK.FIB.02)	None	IPSC.DF.19.11; ESDR.H1.BMP4.TROP; ESDR.H1.MSC; BLD.PER.MONUC.PC; BLD.CD3.PPC; BLD.CD4.CD25M.CD45RA.NPC; SKIN.PEN.FRSK.FIB.01; SKIN.PEN.FRSK.KER.03; THYM.FET; MUS.SKLT.F; MUS.TRNK.FET; MUS.LEG.FET; GI.S.INT.FET; GI.L.INT.FET; GI.RECT.MUC.29; GI.STMC.MUC; GI.DUO.MUC; GI.ESO; GI.STMC.GAST; SPLN	IPSC.DF.19.11; ESDR.H1.NEUR.PROG; ESDR.H1.MSC; SKIN.PEN.FRSK.FIB.02; SKIN.PEN.FRSK.MEL.01; SPLN	ESDR.CD184.ENDO; BLD.CD3.PPC; BLD.CD4.CD25I.CD127.TMEMPC; BLD.CD4.CD25.CD127M.TREGPC; BLD.CD4.CD25M.TPC; BLD.CD4.CD25M.CD45RA.NPC; BLD.CD4.CD25M.IL17M.PL.TPC; BLD.CD4.CD25M.IL17P.PL.TPC; BLD.CD4.CD25M.CD45RO.MPC; BLD.CD4.MPC; BLD.CD4.NPC; BLD.MOB.CD34.PC.F; SKIN.PEN.FRSK.FIB.01; SKIN.PEN.FRSK.FIB.02; SKIN.PEN.FRSK.MEL.01; SKIN.PEN.FRSK.MEL.03; SKIN.PEN.FRSK.KER.03;	IPSC.15b; ESDR.H1.NEUR.PROG; ESDR.H1.MSC; BLD.PER.MONUC.PC; BLD.CD4.NPC; STRM.CHON.MRW.DR.MSC; BRST.MYO; BRN.ANT.CAUD; BRN.CING.GYR; BRN.INF.TMP; BRN.ANG.GYR; MUS.SKLT.F; MUS.SKLT.M; GI.STMC.MUS; GI.RECT.MUC.29; GI.DUO.MUC;	**H3K4me1** SKIN.NHEK;<br><br>**H3K27ac** MUS.HSMMT; BLD.CD14.MONO;	**H3K4me3** None<br><br>**H3K9ac** CRVX.HELAS3.CNCR;	Roadmap MUS.PSOAS<br><br>ENCODE LIV.HEPG2.CNCR

					THYM; BRN.HIPP.MID; BRN.SUB.NIG; BRN.CING.GYR; MUS.SKLT.F; MUS.TRNK.FET; HRT.VNT.R; GI.CLN.SM.MUS; GI.S.INT; GI.RECT.MUC.29; LNG;				
**rs1637365**	None	None	None	None	None	None	**H3K4me1**<br><br>**H3K27ac**	**H3K4me3**<br><br>**H3K9ac**	
**rs181337095**	None	None	None	None	None	None	**H3K4me1**<br>BRN.NHA; BONE.OSTEO<br><br>**H3K27ac**	**H3K4me3**<br><br>**H3K9ac**	
**rs60381548**	None	None	BLD.CD3.PPC; BLD.CD4.CD25I.CD127.TMEMPC; BLD.CD4.CD25.CD127M.TREGPC; BLD.CD4.CD25M.TPC; BLD.CD4.CD25M.CD45RA.NPC; BLD.CD4.CD25M.IL17M.PL.TPC; BLD.CD4.CD25M.IL17P.PL.TPC; BLD.CD4.CD25M.CD45RO.MPC; BLD.CD4.MPC; BLD.CD8.MPC; BLD.CD4.NPC; BLD.CD8.NPC; BLD.CD56.PC; SKIN.PEN.FRSK.KER.03; BRST.HMEC.35; BRST.MYO; BRN.DL.PRFRNTL.CRTX; MUS.PSOAS; MUS.SKLT.F; HRT.VENT.L; HRT.VNT.R; VAS.AOR; GI.DUO.SM.MUS; GI.STMC.MUS; GI.ESO;	GI.S.INT;	ESC.H9; ESC.HUES6; BLD.CD4.CD25.CD127M.TREGPC; BLD.CD4.CD25M.IL17M.PL.TPC; BLD.CD8.MPC; GI.STMC.GAST; LNG;	ESDR.H1.NEUR.PROG; ESDR.H1.MSC;	**H3K4me1**<br>None<br><br>**H3K27ac**<br>BLD.CD14.MONO; SKIN.NHEK; LNG.NHLF; BONE.OSTEO	**H3K4me3**<br>None<br><br>**H3K9ac**<br>None	

			GI.STMC.GAST; LNG.FET; OVRY; PANC; LNG;						

*Highlighted in yellow are breast tissues; in red are the 25-state model active enhancers and promoters and DNase for the breast tissues

Roadmap tissues

LNG.IMR90 IMR90 fetal lung fibroblasts Cell Line; ESC.WA7 ES-WA7 Cells; ESC.H9 H9 Cells; ESC.I3 ES-I3 Cells; ESC.HUES6 HUES6 Cells; ESC.HUES48 HUES48 Cells; ESC.HUES64 HUES64 Cells; ESC.H1 H1 Cells; ESC.4STAR ES-UCSF4 Cells; IPSC.20B iPS-20b Cells; IPSC.18 iPS-18 Cells; IPSC.15b iPS-15b Cells; IPSC.DF.6.9 iPS DF 6.9 Cells; IPSC.DF.19.11 iPS DF 19.11 Cells; ESDR.H1.NEUR.PROG H1 Derived Neuronal Progenitor Cultured Cells; ESDR.H9.NEUR.PROG H9 Derived Neuronal Progenitor Cultured Cells; ESDR.H9.NEUR H9 Derived Neuron Cultured Cells; ESDR.CD56.MESO hESC Derived CD56+ Mesoderm Cultured Cells; ESDR.CD56.ECTO hESC Derived CD56+ Ectoderm Cultured Cells; ESDR.CD184.ENDO hESC Derived CD184+ Endoderm Cultured Cells; ESDR.H1.BMP4.MESO H1 BMP4 Derived Mesendoderm Cultured Cells; ESDR.H1.BMP4.TROP H1 BMP4 Derived Trophoblast Cultured Cells; ESDR.H1.MSC H1 Derived Mesenchymal Stem Cells; BLD.PER.MONUC.PC Primary mononuclear cells from peripheral blood; BLD.CD3.PPC Primary T cells from peripheral blood; BLD.CD4.CD25I.CD127.TMEMPC Primary T cells effector/memory enriched from peripheral blood; BLD.CD3.CPC Primary T cells from cord blood; BLD.CD4.CD25.CD127M.TREGPC Primary T regulatory cells from peripheral blood; BLD.CD4.CD25M.TPC Primary T helper cells from peripheral blood; LD.CD4.CD25M.CD45RA.NPC Primary T helper naive cells from peripheral blood; BLD.CD4.CD25M.IL17M.PL.TPC Primary T helper cells PMA-I stimulated; BLD.CD4.CD25M.IL17P.PL.TPC Primary T helper 17 cells PMA-I stimulated; BLD.CD4.CD25M.CD45RO.MPC Primary T helper memory cells from peripheral blood 1; BLD.CD4.MPC Primary T helper memory cells from peripheral blood 2; BLD.CD8.MPC Primary T CD8+ memory cells from peripheral blood; BLD.CD4.NPC Primary T helper naive cells from peripheral blood; LD.CD8.NPC Primary T CD8+ naive cells from peripheral blood; BLD.CD14.PC Primary monocytes from peripheral blood; BLD.CD19.CPC Primary B cells from cord blood; BLD.CD34.PC Primary hematopoietic stem cells; BLD.MOB.CD34.PC.M Primary hematopoietic stem cells G-CSF-mobilized Male; BLD.MOB.CD34.PC.F Primary hematopoietic stem cells G-CSF-mobilized Female; BLD.CD34.CC Primary hematopoietic stem cells short term culture; BLD.CD19.PPC Primary B cells from peripheral blood; BLD.CD56.PC Primary Natural Killer cells from peripheral blood; BLD.CD15.PC Primary neutrophils from peripheral blood; STRM.MRW.MSC Bone Marrow Derived Cultured Mesenchymal Stem Cells; STRM.CHON.MRW.DR.MSC Mesenchymal Stem Cell Derived Chondrocyte Cultured Cells; FAT.ADIP.DR.MSC Adipose Derived Mesenchymal Stem Cell Cultured Cells; FAT.MSC.DR.ADIP Mesenchymal Stem Cell Derived Adipocyte Cultured Cells; MUS.SAT Muscle Satellite Cultured Cells; SKIN.PEN.FRSK.FIB.01 Foreskin Fibroblast Primary Cells skin01; SKIN.PEN.FRSK.FIB.02 Foreskin Fibroblast Primary Cells skin02; SKIN.PEN.FRSK.MEL.01 Foreskin Melanocyte Primary Cells skin01; SKIN.PEN.FRSK.MEL.03 Foreskin Melanocyte Primary Cells skin03; SKIN.PEN.FRSK.KER.02 Foreskin Keratinocyte Primary Cells skin02; SKIN.PEN.FRSK.KER.03 Foreskin Keratinocyte Primary Cells skin03; BRST.HMEC.35 Breast variant Human Mammary Epithelial Cells (vHMEC); BRST.MYO Breast Myoepithelial Primary Cells; BRN.GANGEM.DR.NRSPHR Ganglion Eminence derived primary cultured neurospheres; BRN.CRTX.DR.NRSPHR Cortex derived primary cultured neurospheres; THYM Thymus; THYM.FET Fetal Thymus; BRN.HIPP.MID Brain Hippocampus Middle; BRN.SUB.NIG Brain Substantia Nigra; BRN.ANT.CAUD Brain Anterior Caudate; BRN.CING.GYR Brain Cingulate Gyrus; BRN.INF.TMP Brain Inferior Temporal Lobe; BRN.ANG.GYR Brain Angular Gyrus; BRN.DL.PRFRNTL.CRTX Brain_Dorsolateral_Prefrontal_Cortex; BRN.GRM.MTRX Brain Germinal Matrix; BRN.FET.F Fetal Brain Female; BRN.FET.M Fetal Brain Male; FAT.ADIP.NUC Adipose Nuclei; MUS.PSOAS Psoas Muscle; MUS.SKLT.F Skeletal Muscle Female; MUS.SKLT.M Skeletal Muscle Male; MUS.TRNK.FET Fetal Muscle Trunk; MUS.LEG.FET Fetal Muscle Leg; HRT.FET Fetal Heart; HRT.ATR.R Right Atrium; HRT.VENT.L Left Ventricle; HRT.VNT.R Right Ventricle; VAS.AOR Aorta; GI.DUO.SM.MUS; Duodenum Smooth Muscle; GI.CLN.SM.MUS Colon Smooth Muscle; GI.RECT.SM.MUS Rectal Smooth Muscle; GI.STMC.MUS Stomach Smooth Muscle; GI.STMC.FET Fetal Stomach; GI.S.INT.FET Fetal Intestine Small; GI.L.INT.FET Fetal Intestine Large; GI.S.INT Small Intestine; GI.CLN.SIG Sigmoid Colon; GI.CLN.MUC Colonic Mucosa; GI.RECT.MUC.29 Rectal Mucosa Donor 29; GI.RECT.MUC.31 Rectal Mucosa Donor 31; GI.STMC.MUC Stomach Mucosa; GI.DUO.MUC Duodenum Mucosa; GI.ESO Esophagus; GI.STMC.GAST Gastric; PLCNT.AMN Placenta Amnion; KID.FET Fetal Kidney; LNG.FET Fetal Lung; OVRY Ovary; PANC.ISLT Pancreatic Islets; ADRL.GLND.FET Fetal Adrenal Gland; PLCNT.FET Placenta; LIV.ADLT Liver; PANC Pancreas; LNG Lung; SPLN Spleen

ENCODE tissues

LNG.A549.ETOH002.CNCR A549 EtOH 0.02pct Lung Carcinoma Cell Line; BLD.DND41.CNCR Dnd41 TCell Leukemia Cell Line; BLD.GM12878 GM12878 Lymphoblastoid Cells; CRVX.HELAS3.CNCR HeLa-S3 Cervical Carcinoma Cell Line; LIV.HEPG2.CNCR HepG2 Hepatocellular Carcinoma Cell Line; BRST.HMEC HMEC Mammary Epithelial Primary Cells; MUS.HSMM HSMM Skeletal Muscle Myoblasts Cells; MUS.HSMMT HSMM cell derived Skeletal Muscle Myotubes Cells; VAS.HUVEC HUVEC Umbilical Vein Endothelial Primary Cells; BLD.K562.CNCR K562 Leukemia Cells; BLD.CD14.MONO Monocytes-

CD14+ RO01746 Primary Cells; BRN.NHA NH-A Astrocytes Primary Cells; SKIN.NHDFAD NHDF-Ad Adult Dermal Fibroblast Primary Cells; SKIN.NHEK NHEK-Epidermal Keratinocyte Primary Cells; LNG.NHLF NHLF Lung Fibroblast Primary Cells; BONE.OSTEO Osteoblast Primary Cells

Description of the 25-state model using 12 imputed marks

TssA Active TSS; PromU Promoter Upstream TSS; PromD1 Promoter Downstream TSS 1; PromD2 Promoter Downstream TSS 2; Tx5′ Transcribed - 5' preferential; Tx Strong transcription; Tx3′ Transcribed - 3' preferential; TxWk Weak transcription; TxReg Transcribed & regulatory (Prom/Enh); TxEnh5′ Transcribed 5' preferential and Enh; TxEnh3′ Transcribed 3' preferential and Enh; TxEnhW Transcribed and Weak Enhancer; EnhA1 Active Enhancer 1; EnhA2 Active Enhancer 2; EnhAF Active Enhancer Flank; EnhW1 Weak Enhancer 1; EnhW2 Weak Enhancer 2; EnhAc Primary H3K27ac possible Enhancer; DNase Primary DNase; ZNF/Rpts ZNF genes & repeats; Het Heterochromatin; PromP Poised Promoter; PromBiv Bivalent Promoter; ReprPC Repressed Polycomb; Quies Quiescent/Low.

Supplementary Table S8. Evaluation of identified SNPs in Latino GWAS

							Overall breast cancer risk		ER+ breast cancer		ER- breast cancer		GWA in discovery study
**Index SNPs**	**Chr**	**Position**	**Test**	**Other**	**Locus**	**TAF* in Latino**	**OR (95% CI)**	**P**	**OR (95% CI)**	**P**	**OR (95% CI)**	**P**	
rs17024629	1	110,179,756	T	C	1p13.3	0.094	0.99 (0.87 - 1.13)	0.91	1.04 (0.87 - 1.24)	0.68	0.90 (0.67 - 1.22)	0.51	Overall
rs67931591	1	215,330,292	G	GCTGAGG-CAGGAGA	1q41	0.637	1.00 (0.93 - 1.08)	0.98	1.02 (0.91 - 1.13)	0.77	0.93 (0.78 - 1.10)	0.41	ER negative
rs2522057	5	131,801,947	C	G	5q31.1	0.728	0.98 (0.90 - 1.07)	0.63	1.00 (0.88 - 1.12)	0.94	1.05 (0.87 - 1.27)	0.62	Overall
rs1637365	7	74,359,358	T	C	7q11.23	0.225	1.06 (0.97 - 1.16)	0.19	1.10 (0.98 - 1.25)	0.11	0.96 (0.78 - 1.18)	0.67	ER negative
rs1869959	15	75,147,332	A	C	15q24.1	0.311	0.96 (0.88 - 1.03)	0.26	0.92 (0.82 - 1.03)	0.13	0.96 (0.80 - 1.15)	0.65	Overall
rs60381548	15	75,728,474	CA	C	15q24.2	0.266	0.97 (0.89 - 1.06)	0.52	0.99 (0.88 - 1.11)	0.85	0.86 (0.71 - 1.04)	0.11	Overall
rs181337095	15	100,907,094	A	G	15q26.3	0.866	0.92 (0.82 - 1.03)	0.13	0.94 (0.81 - 1.09)	0.41	0.80 (0.64 - 1.01)	0.06	ER negative
**SNPs in same loci as index SNPs and independently associated with breast cancer**													
rs5780828	1	215,416,434	TA	T	1q41	0.135	1.09 (0.98 - 1.22)	0.11	1.16 (1.00 - 1.34)	0.05	1.01 (0.80 - 1.29)	0.91	ER negative
rs116363925	1	109,926,599	T	G	1p13.3	0.028	0.82 (0.61 - 1.11)	0.20	0.89 (0.61 - 1.29)	0.53	0.72 (0.36 - 1.45)	0.36	Overall
5:132149322:-G:GGCCGC-CGCC	5	132,149,322	GGCCG-CCGCC	G	5q31.1	0.143	1.04 (0.93 - 1.16)	0.53	1.02 (0.88 - 1.18)	0.83	1.07 (0.84 - 1.35)	0.60	Overall
rs117793215	15	100,535,681	T	C	15q26.3	0.022	0.83 (0.62 - 1.12)	0.23	0.69 (0.46 - 1.05)	0.09	1.41 (0.84 - 2.36)	0.19	Overall
rs113939578	15	75,479,704	T	C	15q24.2	0.092	1.19 (1.04 - 1.36)	0.01	1.19 (0.99 - 1.42)	0.06	1.34 (1.02 - 1.77)	0.04	Overall

*TAF – Allele frequency

Suppl Fig 1a :GWAS meta-analysis Manhattan plot for overall breast cancer risk

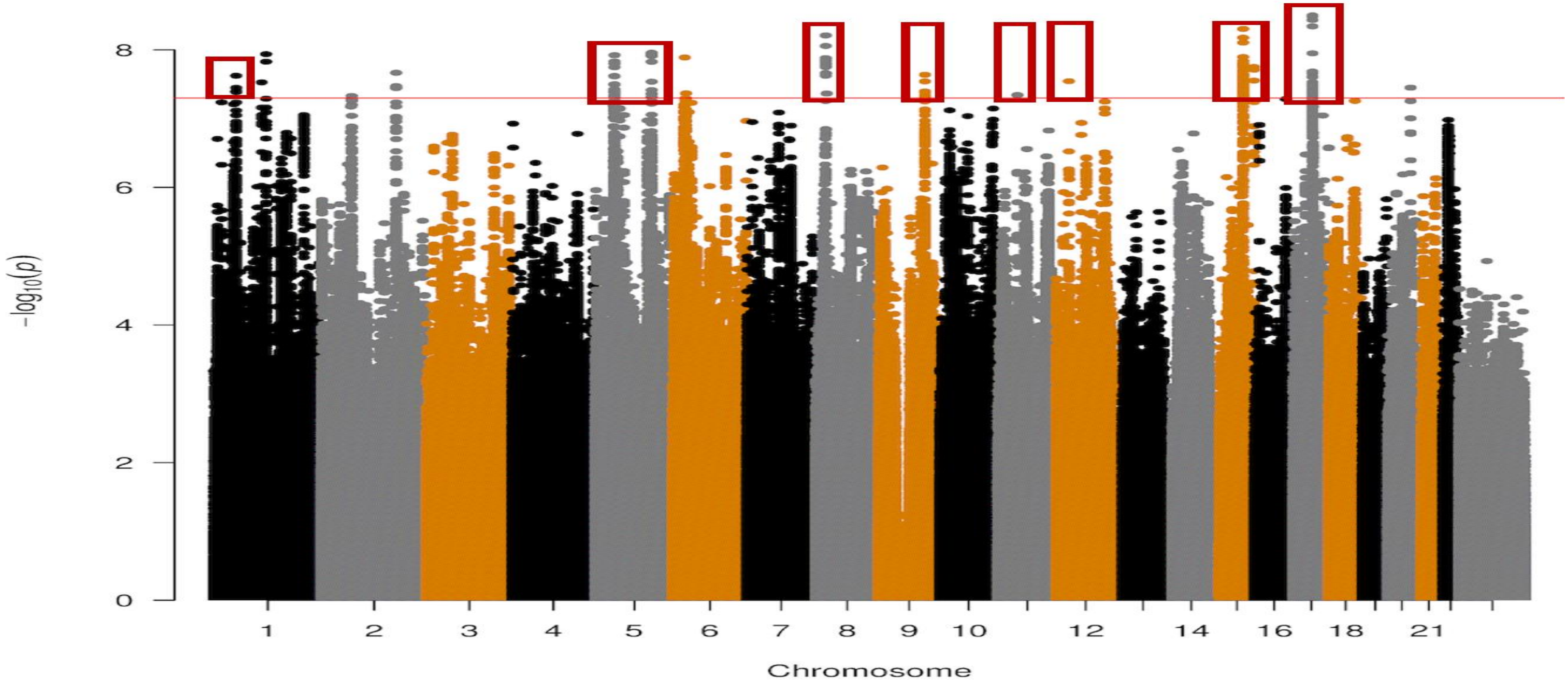

The plot shows the loci significant after removing SNPs within 500kb flank of the index SNPs at previously known breast cancer risk loci and SNPs with BCAC P-value < 5*10-8.
The red rectangles show the regions with AA P value<0.05.

Suppl Fig 1b: GWAS meta-analysis Manhattan plot for ER negative breast cancer

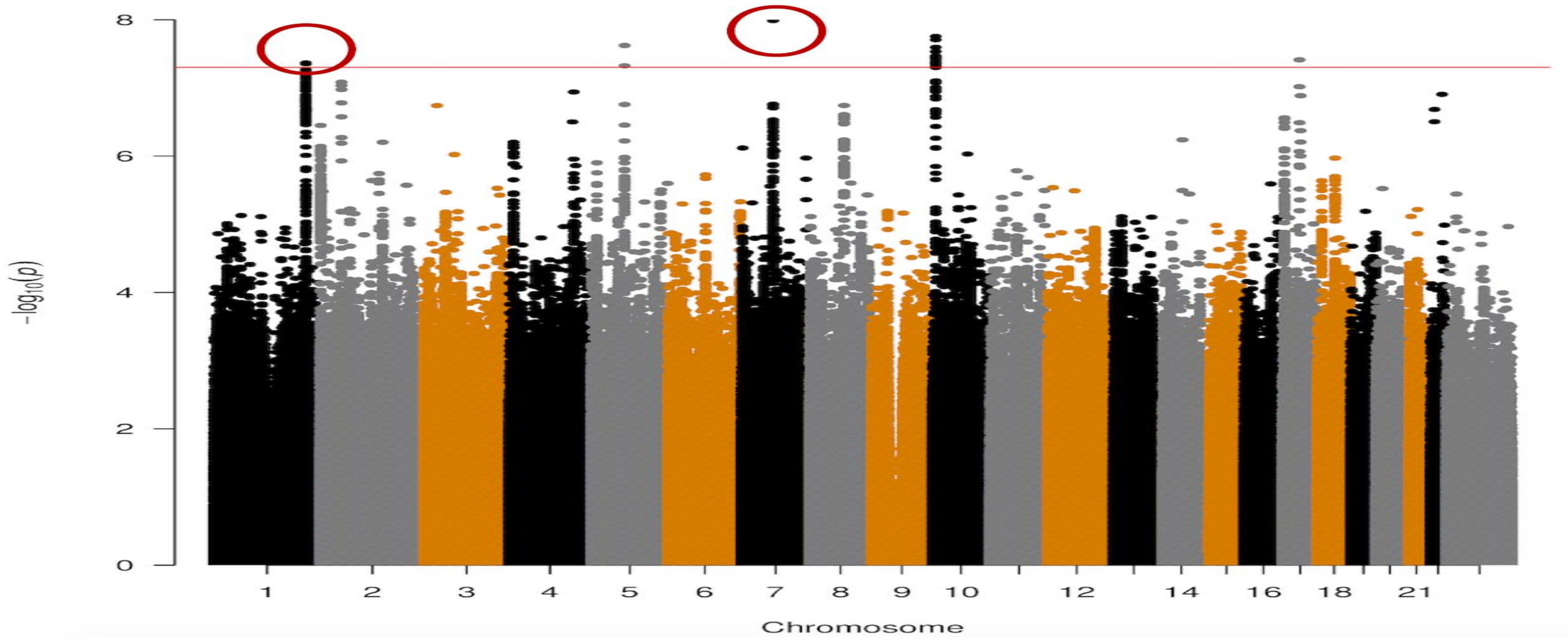

The plot shows the loci significant after removing SNPs within 500kb flank of the index SNPs at previously known breast cancer risk loci and SNPs with BCAC P-value < 5*10-8.
The red circles show the regions with AA P value<0.05.

Supplementary Fig 2a

15q24 region - European

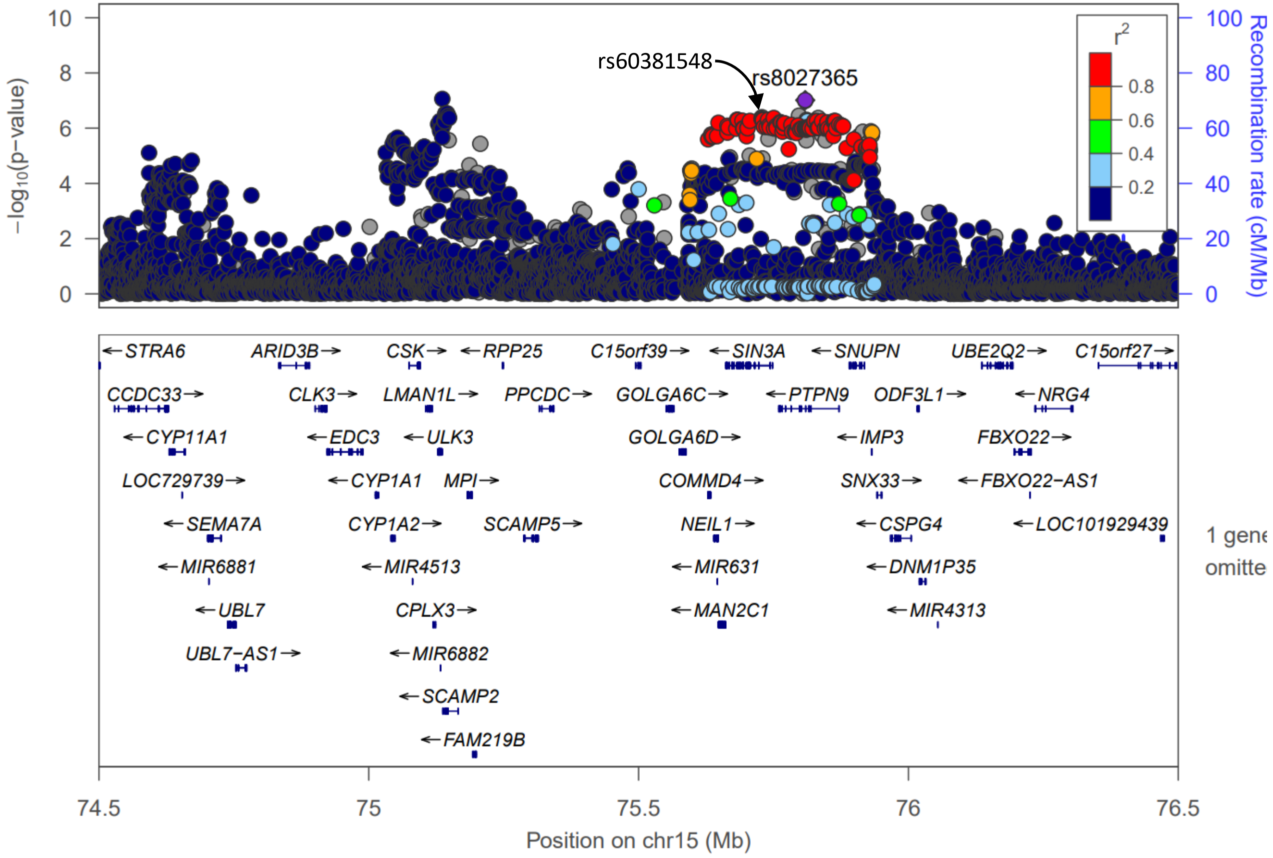

15q24 region - African

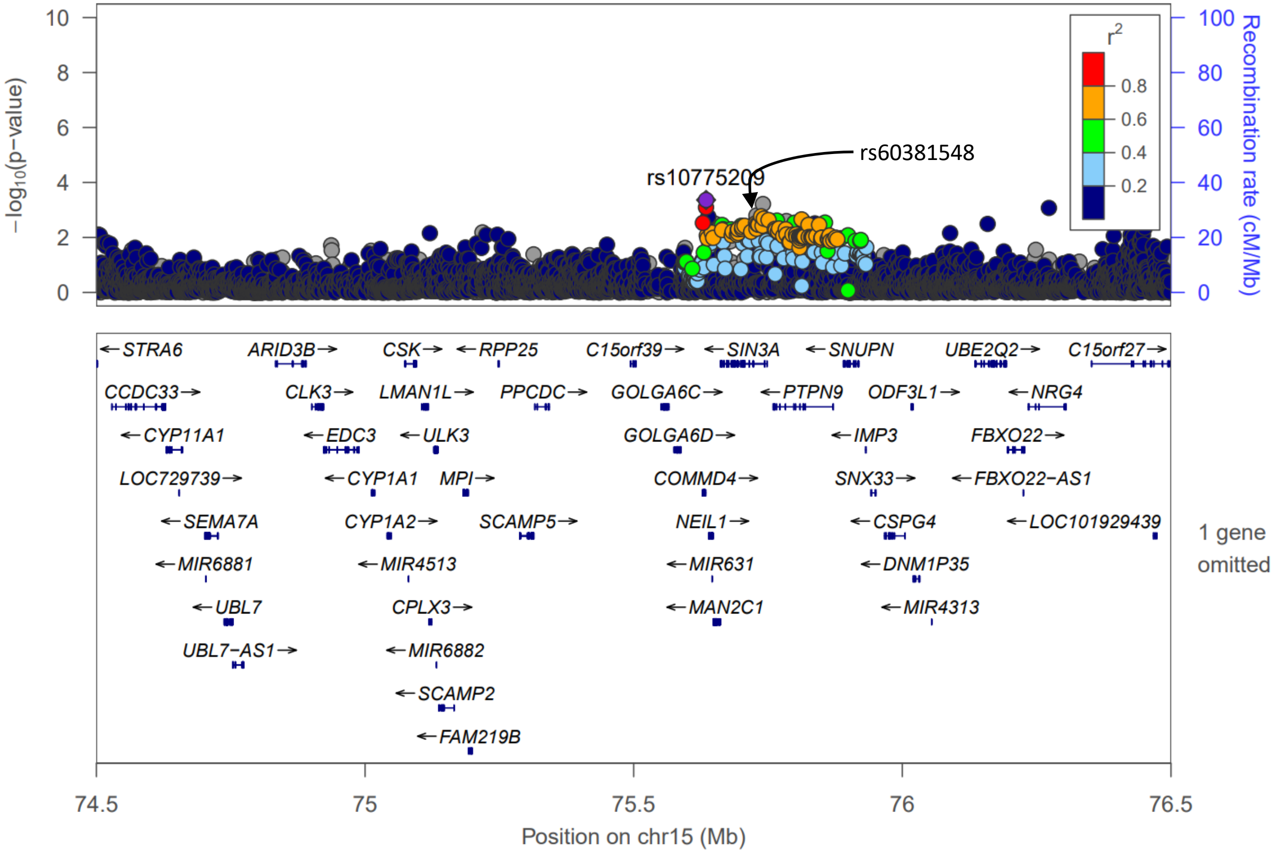

Supplementary Fig 2b

rs60381548 - European

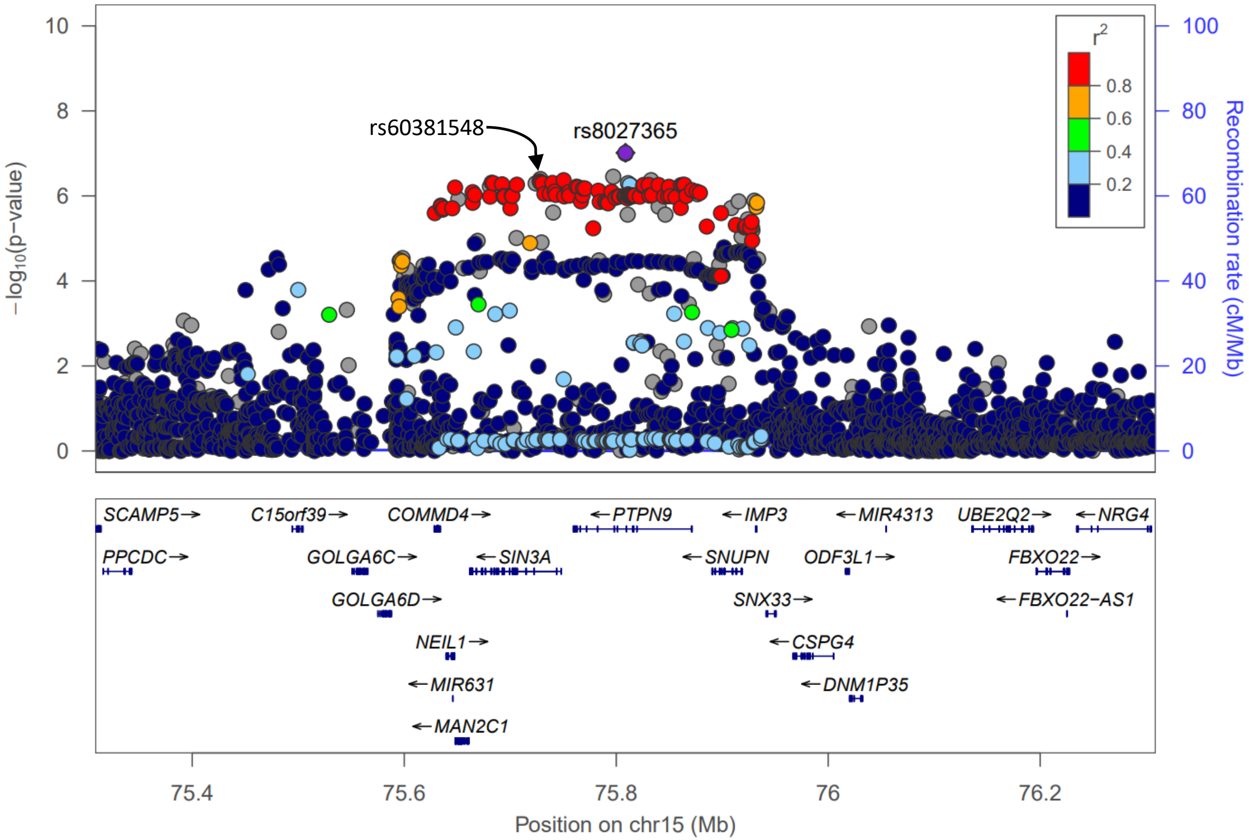

rs60381548 - African

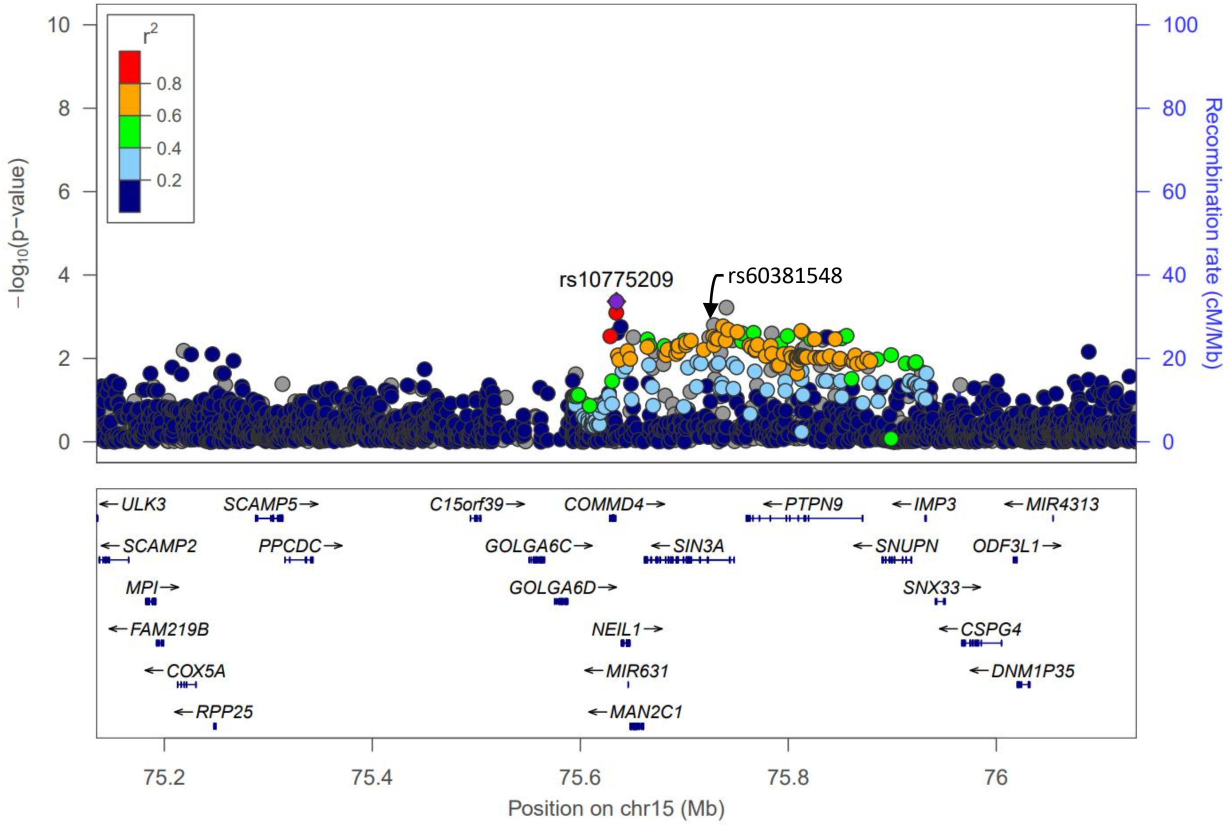

Supplementary Fig 2c

rs1869959 - European

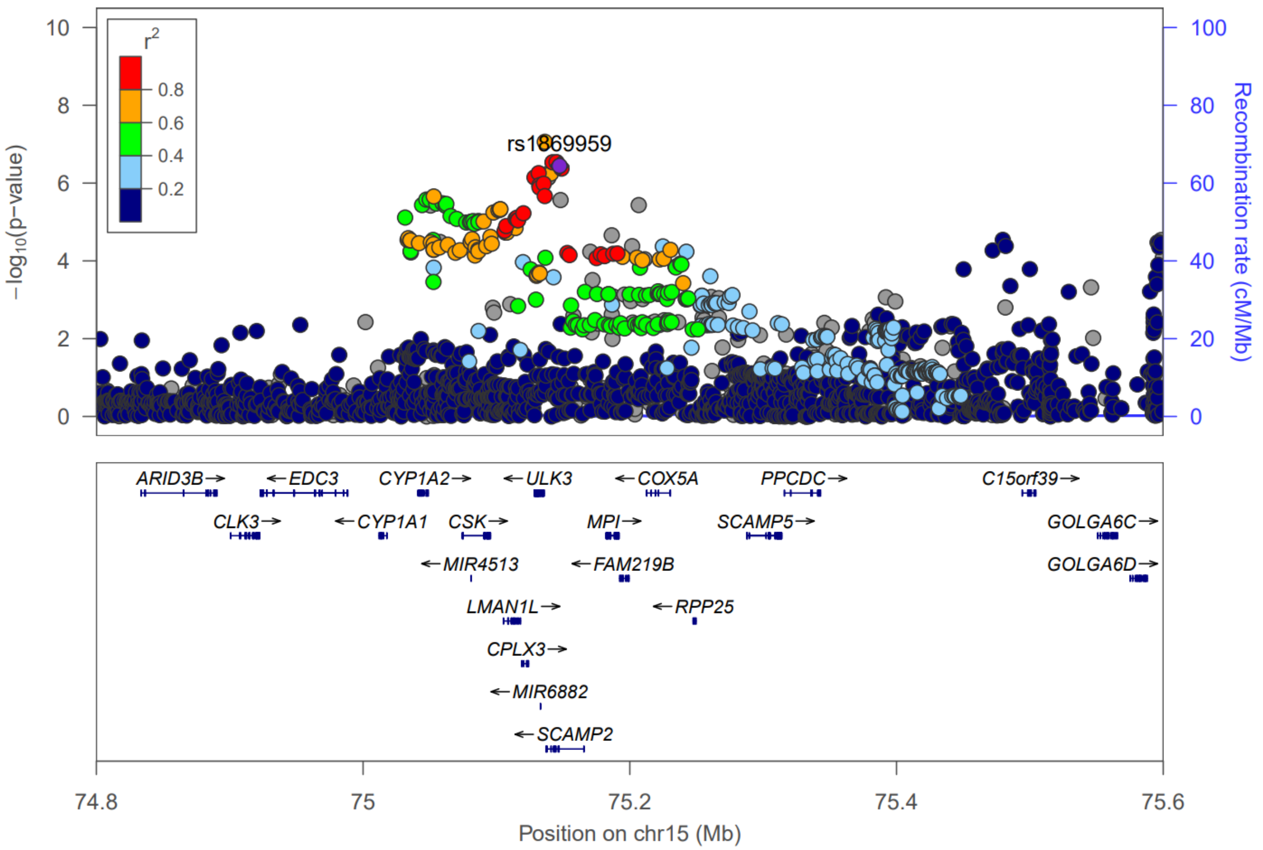

rs1869959 - African

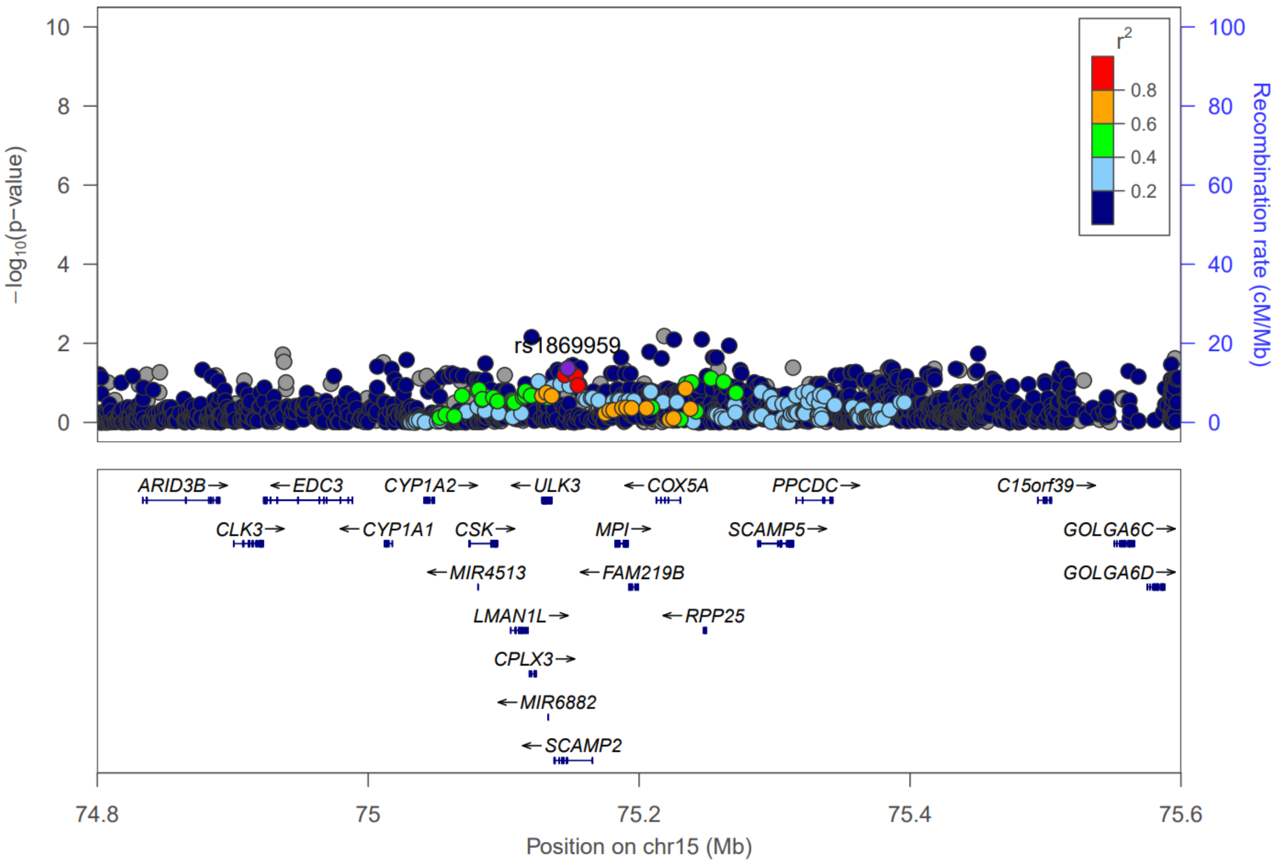

Supplementary Fig 2d

rs2522057 - European

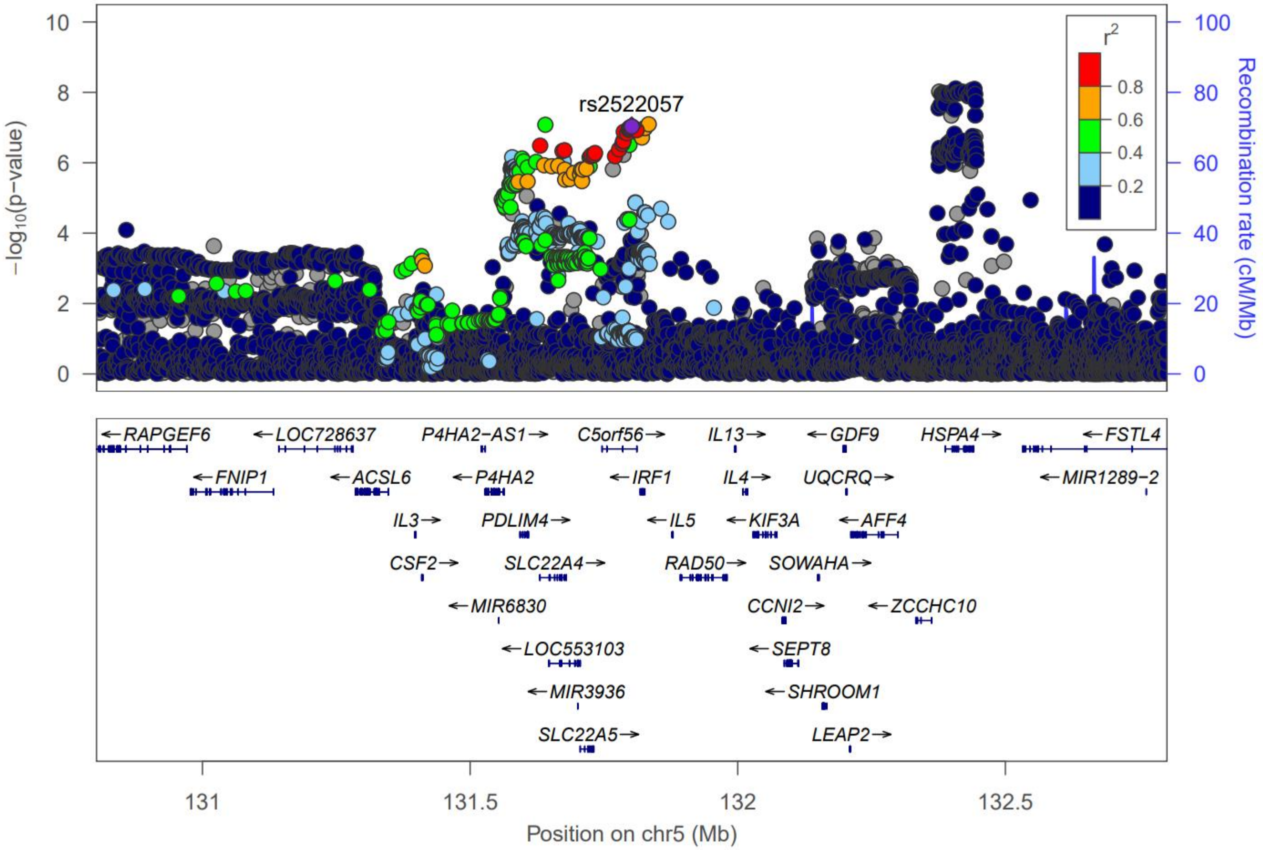

rs2522057 - African

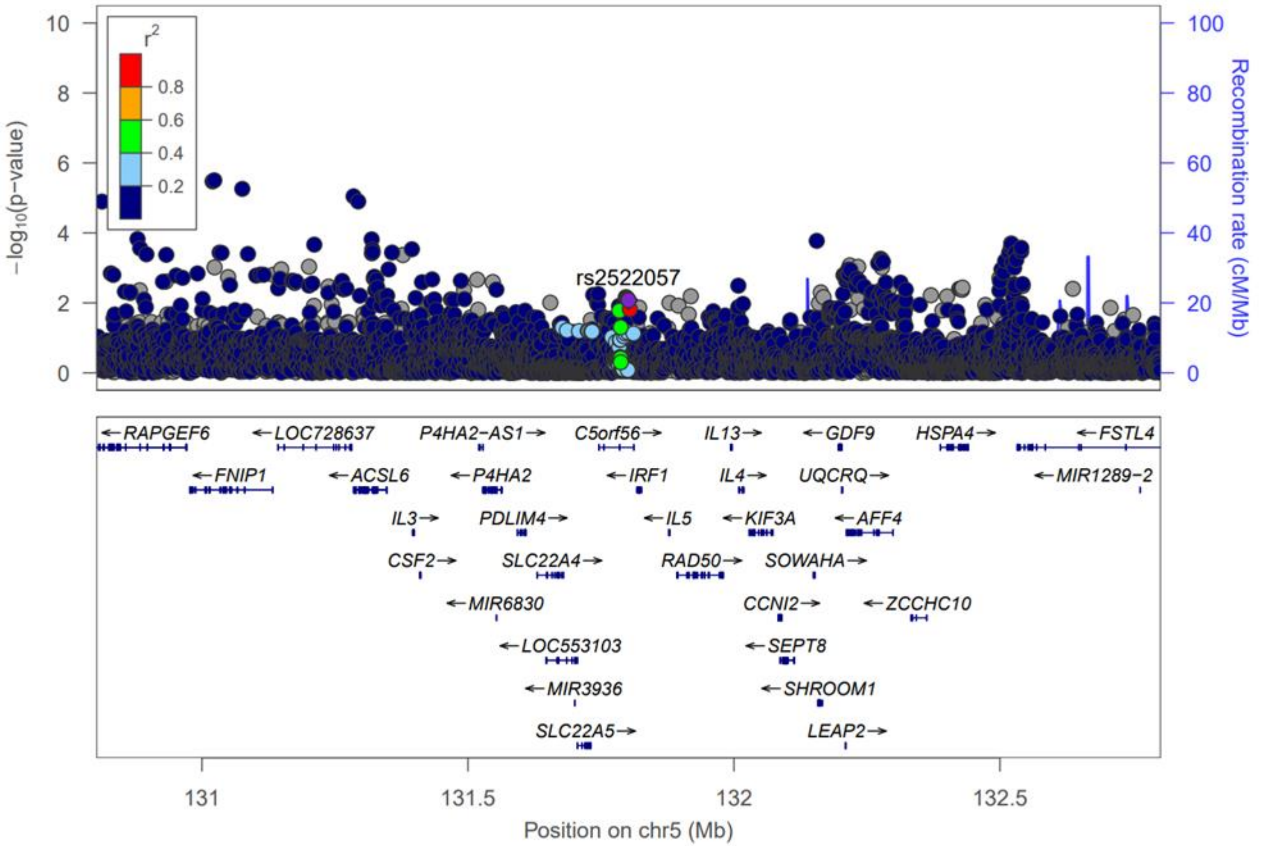

Supplementary Fig 2e

rs17024629 - European

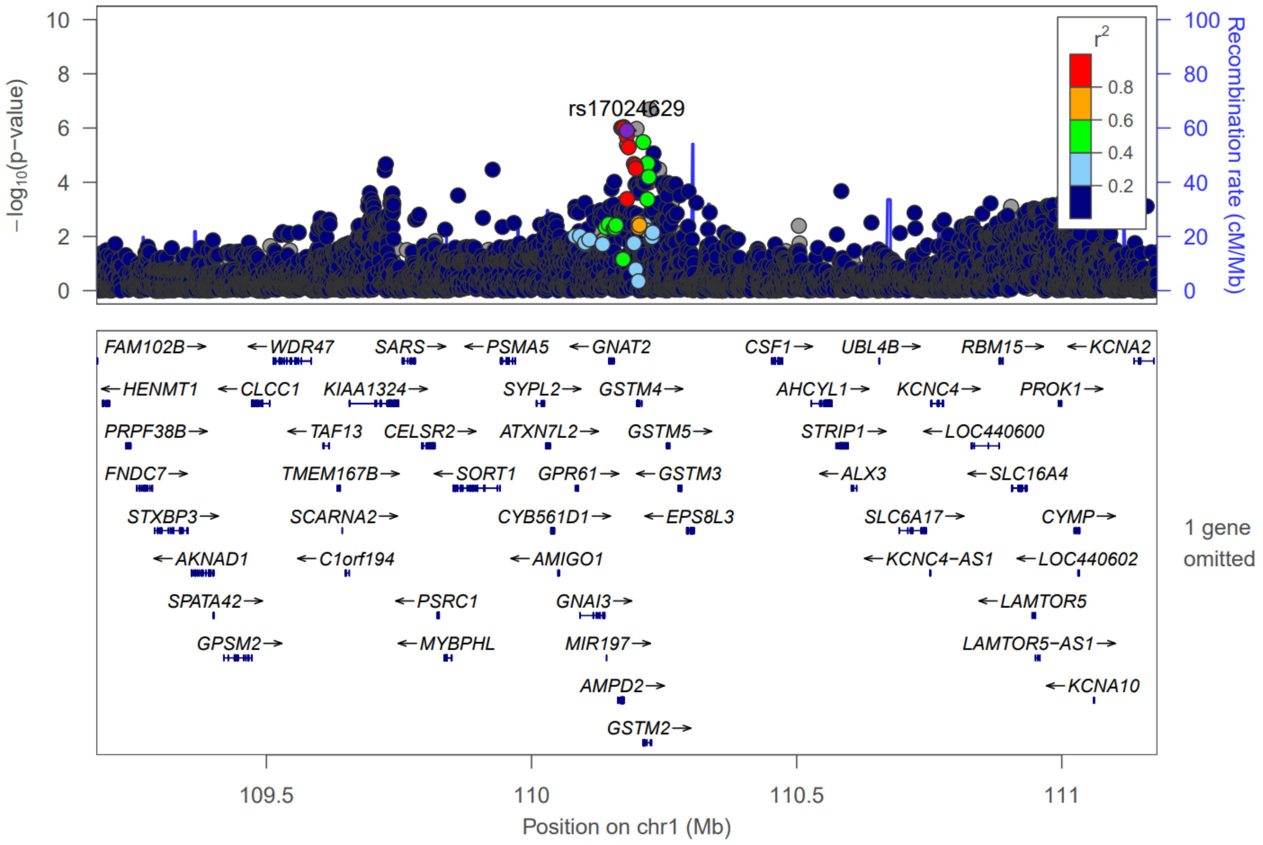

rs17024629 - African

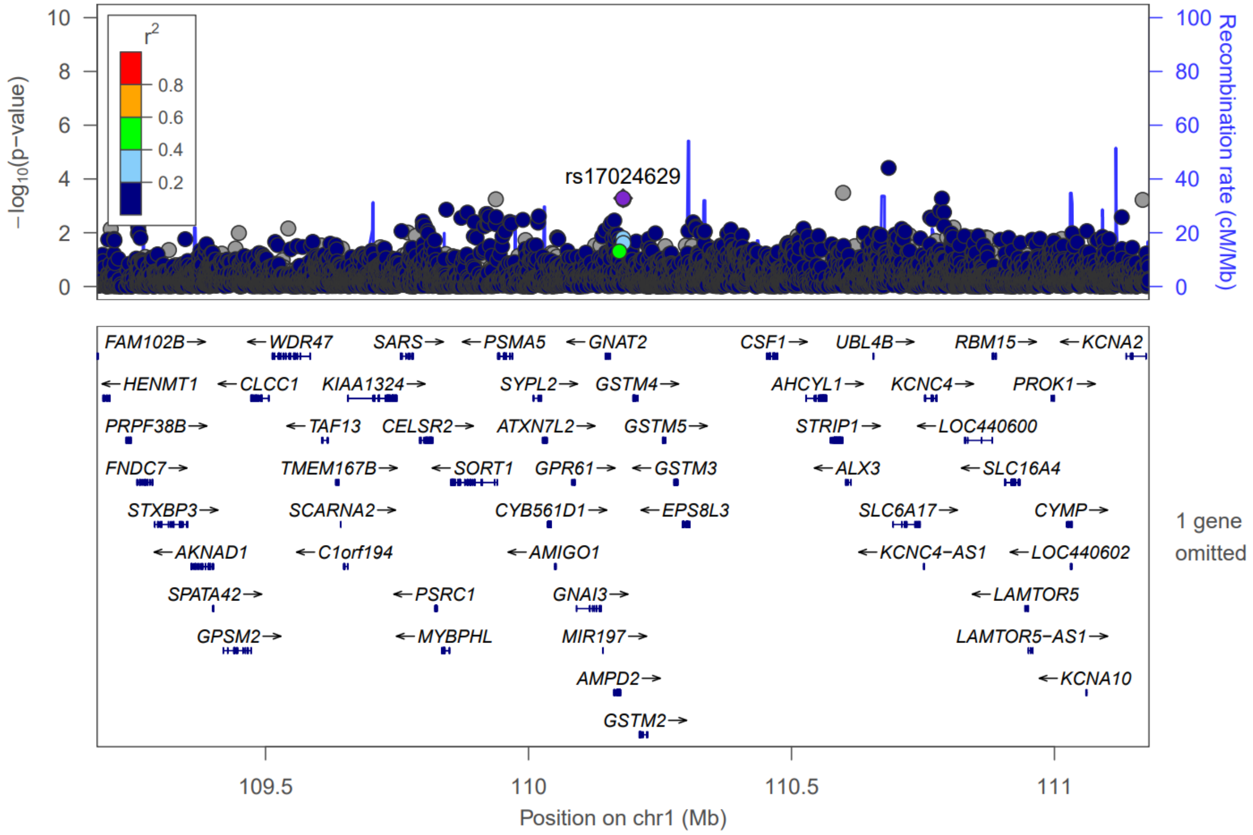

Supplementary Fig 2f

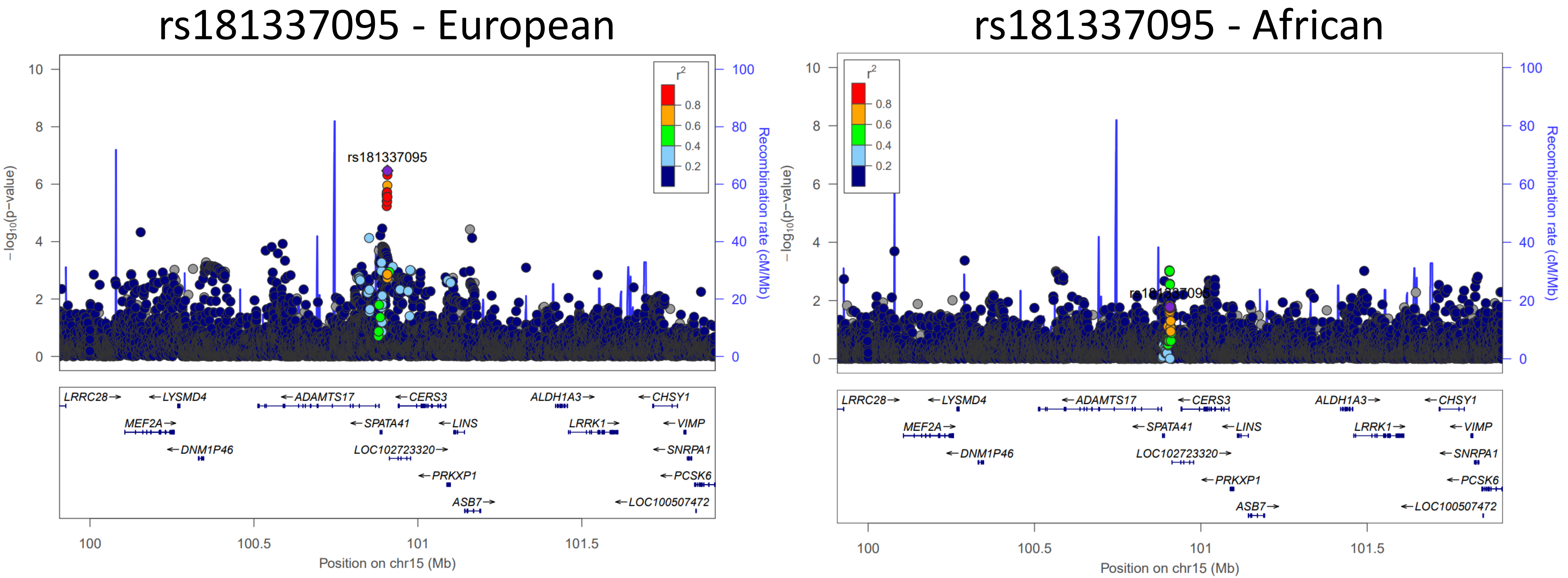

Supplementary Fig 2g

rs67931591 - European

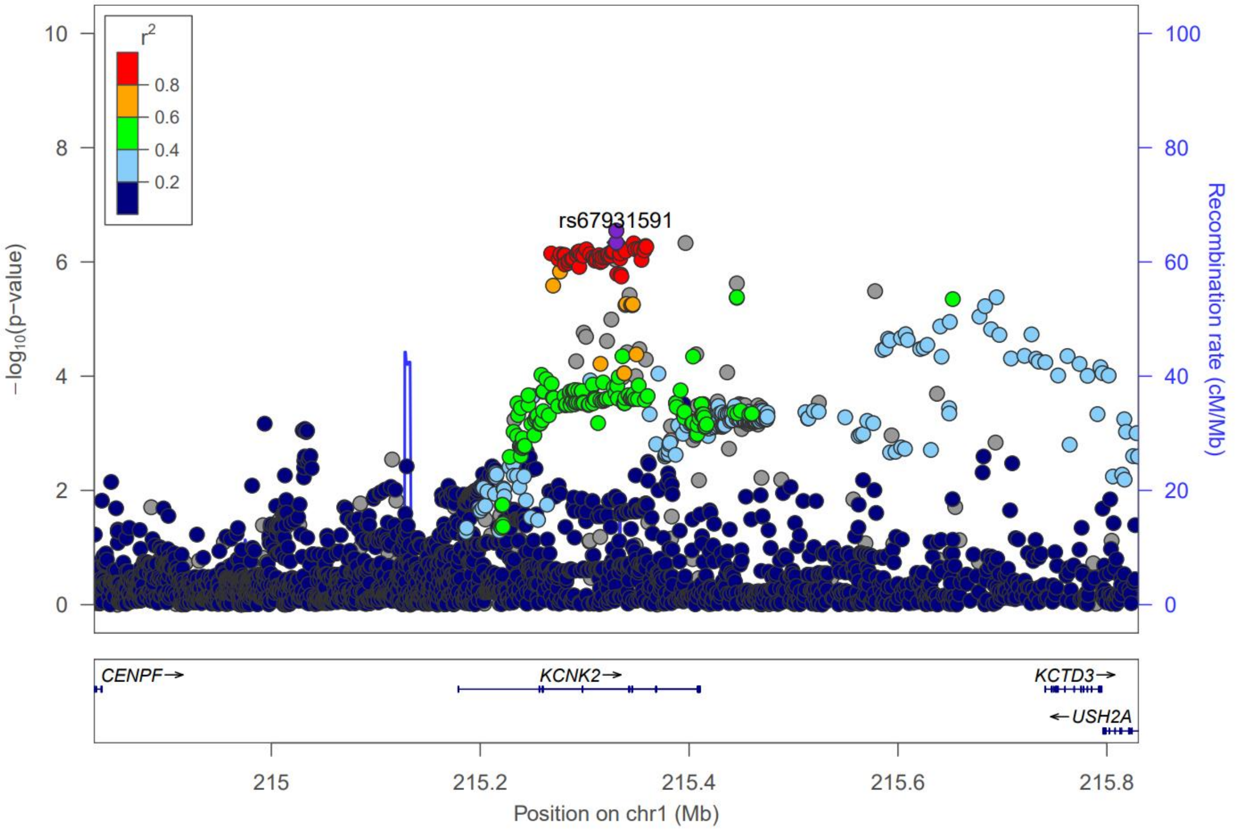

rs67931591 - African

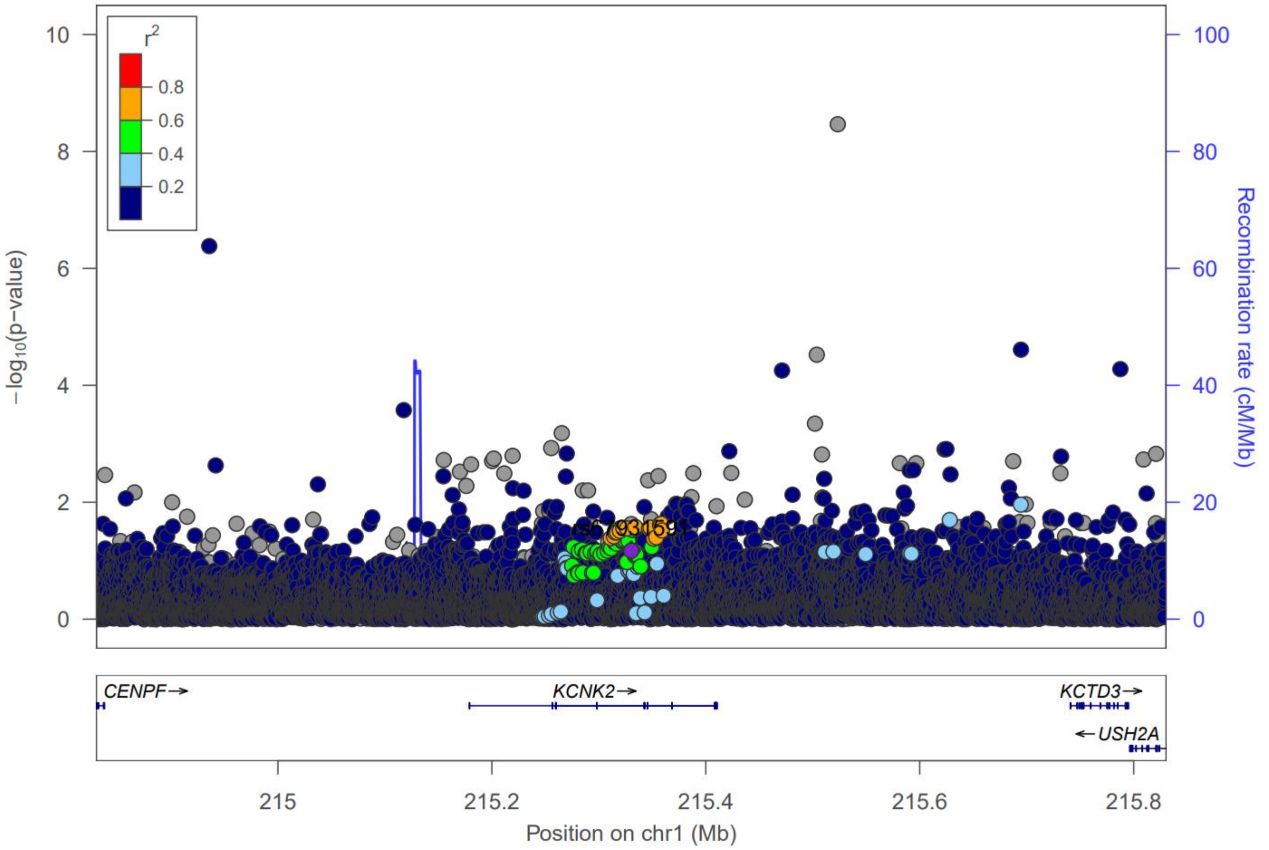

Supplementary Fig 2h

rs1637365 - European

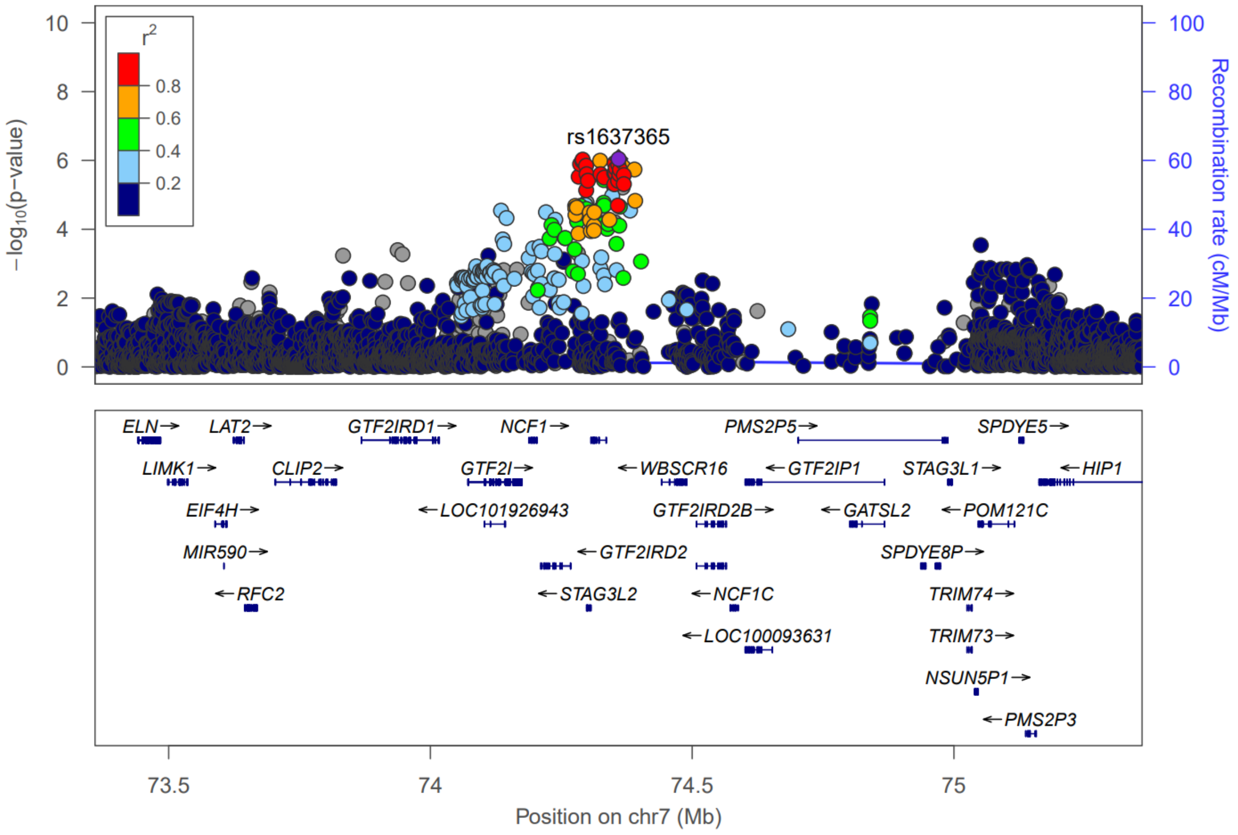

rs1637365 - African

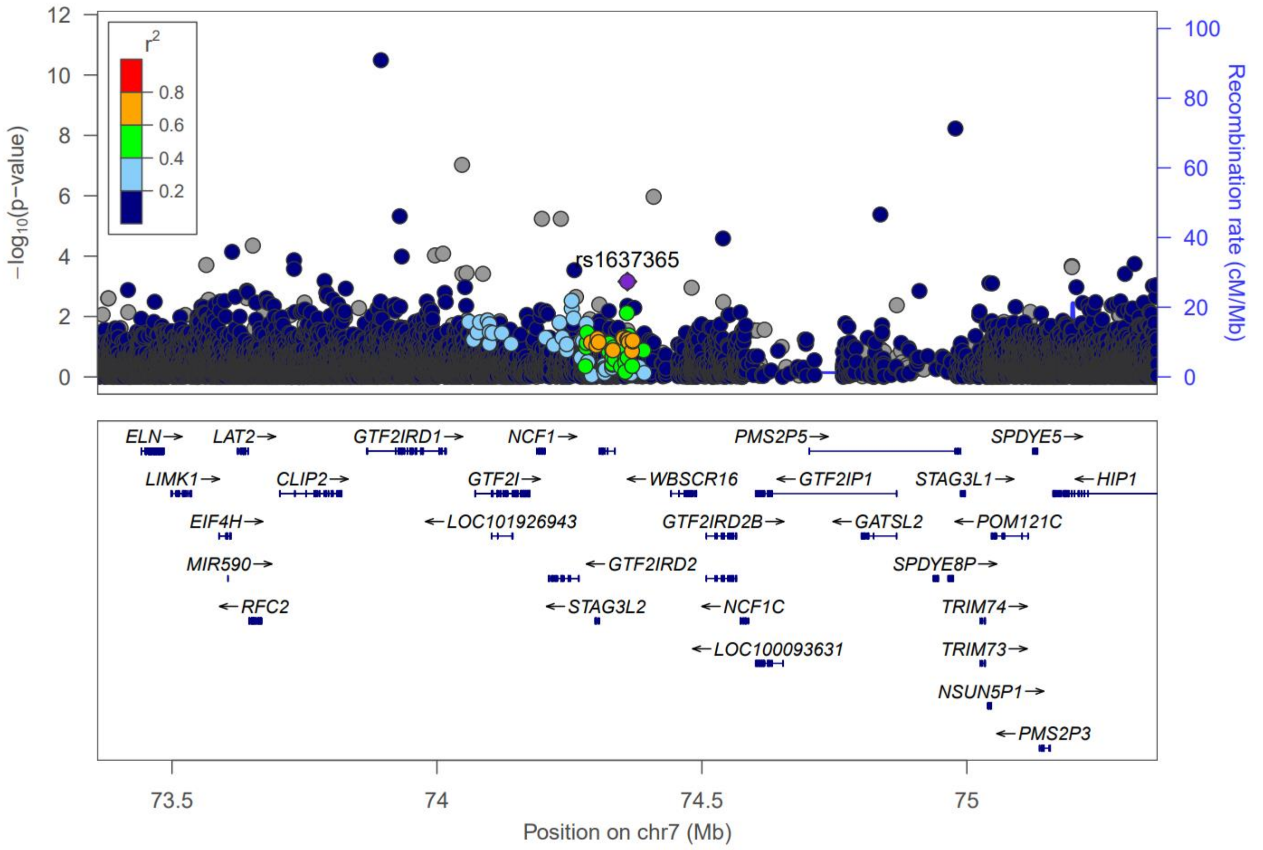

Supplementary Fig 2: LocusZoom Plots of Candidate Loci

(a-h) are LocusZoom plots made using the online tool at locuszoom.org. Each point is a SNP where the x-axis is the chromosomal position, and the y-axis is the $-\log_{10}(P)$ of the association between the SNP and overall breast cancer status (a-f) or ER-negative breast cancer status (g-h) [15]. The color of the point indicated the LD R^2 value between the colored SNP and the index SNP indicated by a purple diamond. Plots labeled “European” are plotted over hg19/1000 Genomes Nov 2014 European reference panel. These plots depict the association in the BCAC GWAS. Plots labeled “African” are plotted over hg19/1000 Genomes Nov 2014 African reference panel. These plots depict the association in the meta-analysis of all five African Ancestries GWAS.

Supplementary References

1. Jones AV, Tilley M, Gutteridge A, Hyde C, Nagle M, etal. GWAS of self-reported mosquito bite size, itch intensity and attractiveness to mosquitoes implicates immune-related predisposition loci. Hum Mol Genet. 2017 Apr 1;26(7):1391-1406. doi: 10.1093/hmg/ddx036. PMID: 28199695; PMCID: PMC5390679.

2. Astle WJ, Elding H, Jiang T, Allen D, Ruklisa D, etal. The Allelic Landscape of Human Blood Cell Trait Variation and Links to Common Complex Disease. Cell. 2016 Nov 17;167(5):1415-1429.e19. doi: 10.1016/j.cell.2016.10.042. PMID: 27863252; PMCID: PMC5300907.

3. Ferreira MAR, Mathur R, Vonk JM, Szwajda A, Brumpton B etal. Genetic Architectures of Childhood- and Adult-Onset Asthma Are Partly Distinct. Am J Hum Genet. 2019 Apr 4;104(4):665-684. doi: 10.1016/j.ajhg.2019.02.022. Epub 2019 Mar 28. PMID: 30929738; PMCID: PMC6451732.

4. Feitosa MF, Kraja AT, Chasman DI, Sung YJ, Winkler TW etal. Novel genetic associations for blood pressure identified via gene-alcohol interaction in up to 570K individuals across multiple ancestries. PLoS One. 2018 Jun 18;13(6):e0198166. doi: 10.1371/journal.pone.0198166. PMID: 29912962; PMCID: PMC6005576.

5. Ehret GB, Ferreira T, Chasman DI, Jackson AU, Schmidt EM, etal The genetics of blood pressure regulation and its target organs from association studies in 342,415 individuals. Nat Genet. 2016 Oct;48(10):1171-1184. doi: 10.1038/ng.3667. Epub 2016 Sep 12. PMID: 27618452; PMCID: PMC5042863.

6. Coronary Artery Disease (C4D) Genetics Consortium. A genome-wide association study in Europeans and South Asians identifies five new loci for coronary artery disease. Nat Genet. 2011 Mar 6;43(4):339-44. doi: 10.1038/ng.782. PMID: 21378988.

7. Lee JJ, Wedow R, Okbay A, Kong E, Maghzian O, etal. Gene discovery and polygenic prediction from a genome-wide association study of educational attainment in 1.1 million individuals. Nat Genet. 2018 Jul 23;50(8):1112-1121. doi: 10.1038/s41588-018-0147-3. PMID: 30038396; PMCID: PMC6393768.

8. Wu Y, Byrne EM, Zheng Z, Kemper KE, Yengo L, etal. Genome-wide association study of medication-use and associated disease in the UK Biobank. Nat Commun. 2019 Apr 23;10(1):1891. doi: 10.1038/s41467-019-09572-5. PMID: 31015401; PMCID: PMC6478889.

9. Michailidou K, Lindström S, Dennis J, Beesley J, Hui S, etal. Association analysis identifies 65 new breast cancer risk loci. Nature. 2017 Nov 2;551(7678):92-94. doi: 1 0.1038/nature24284. Epub 2017 Oct 23. PMID: 29059683; PMCID: PMC5798588.

10. Okada Y, Kamatani Y, Takahashi A, Matsuda K, Hosono N, etal. A genome-wide association study in 19 633 Japanese subjects identified LHX3-QSOX2 and IGF1 as adult height loci. Hum Mol Genet. 2010 Jun 1;19(11):2303-12. doi: 10.1093/hmg/ddq091. Epub 2010 Feb 26. PMID: 20189936.

11. Rüeger S, McDaid A, Kutalik Z. Evaluation and application of summary statistic imputation to discover new height-associated loci. PLoS Genet. 2018 May 21;14(5):e1007371. doi: 10.1371/journal.pgen.1007371. PMID: 29782485; PMCID: PMC5983877.

12. Kichaev G, Bhatia G, Loh PR, Gazal S, Burch K, Freund MK, Schoech A, Pasaniuc B, Price AL. Leveraging Polygenic Functional Enrichment to Improve GWAS Power. Am J Hum Genet. 2019 Jan 3;104(1):65-75. doi: 10.1016/j.ajhg.2018.11.008. Epub 2018 Dec 27. PMID: 30595370; PMCID: PMC6323418.

13. Wuttke M, Li Y, Li M, Sieber KB, Feitosa MF, Gorski M, Tin A, etal. A catalog of genetic loci associated with kidney function from analyses of a million individuals. Nat Genet. 2019 Jun;51(6):957-972. doi: 10.1038/s41588-019-0407-x. Epub 2019 May 31. PMID: 31152163; PMCID: PMC6698888.

14. Kichaev G, Bhatia G, Loh PR, Gazal S, Burch K, Freund MK, Schoech A, Pasaniuc B, Price AL. Leveraging Polygenic Functional Enrichment to Improve GWAS Power. Am J Hum Genet. 2019 Jan 3;104(1):65-75. doi: 10.1016/j.ajhg.2018.11.008. Epub 2018 Dec 27. PMID: 30595370; PMCID: PMC6323418.

15. Pruim RJ, Welch RP, Sanna S, Teslovich TM, Chines PS, Gliedt TP, et al. LocusZoom : Regional visualization of genome wide association scan results. Bioinformatics. 2011;27(13):2336 2337. pmid:20634204
